# Whole Proteome Analysis of Mouse Lymph Nodes in Cutaneous Anthrax

**DOI:** 10.1371/journal.pone.0110873

**Published:** 2014-10-20

**Authors:** Taissia G. Popova, Virginia Espina, Weidong Zhou, Claudius Mueller, Lance Liotta, Serguei G. Popov

**Affiliations:** School of Systems Biology, George Mason University, Manassas, Virginia, United States of America; Loyola University Medical Center, United States of America

## Abstract

This study aimed to characterize a soluble proteome of popliteal lymph nodes during lymphadenitis induced by intradermal injection of *Bacillus anthracis* Sterne spores in mice using tandem LC-MS/MS and reverse-phase protein microarray with antibodies specific to epitopes of phosphorylated proteins. More than 380 proteins were detected in the normal intra-nodal lymph, while the infectious process resulted in the profound changes in the protein abundances and appearance of 297 unique proteins. These proteins belong to an array of processes reflecting response to wounding, inflammation and perturbations of hemostasis, innate immune response, coagulation and fibrinolysis, regulation of body fluid levels and vascular disturbance among others. Comparison of lymph and serum revealed 83 common proteins. Also, using 71 antibodies specific to total and phosphorylated forms of proteins we carried initial characterization of circulating lymph phosphoproteome which brought additional information regarding signaling pathways operating in the lymphatics. The results demonstrate that the proteome of intra-nodal lymph serves as a sensitive sentinel of the processes occurring within the lymph nodes during infection. The acute innate response of the lymph nodes to anthrax is accompanied by cellular damage and inflammation with a large number of up- and down-regulated proteins many of which are distinct from those detected in serum. MS data are available via ProteomeXchange with identifier PXD001342.

## Introduction

Lymphoid organs and tissues of the host play key roles in the protection of the host from infections and spread of tumors. The peripheral pre-nodal lymph is formed in the interstitial space around the capillary beds as a result of a tissue fluid filtration process driven by the hydrostatic pressure in the arterial end of capillaries. The lymphatic capillaries merge into progressively bigger vessels that transport the pre-nodal lymph to nodes disseminated throughout the body. Draining lymph nodes (LNs) collect and filter lymphatic fluid-carried antigens from every parenchymal organ and tissue. The lymph then proceeds to the high endothelial venules in the nodal sinus, and ultimately becomes returned to the blood circulation via the subclavian vein [1].

Lymph contains a large number of lymphocytes, macrophages, and many plasma proteins including those synthesized and secreted by tissue cells. Historically, characterization of lymph has been elusive due to the difficulty of collecting samples from the lymphatics. Until recently the dominating notion was that the proteomic profile of pre-nodal afferent lymph mostly overlapped with that of plasma, since the former was considered to be an ultrafiltrate of the latter. This notion was challenged by a few comparative analyses of lymph and plasma, which reported the presence of specific proteins in one fluid but not the other [2]–[5]. However, information regarding the protein content of lymph remains largely incomplete, and relatively little is known about the extent to which the lymph proteome compares with that of plasma [3], [6]–[8].

Recent analyses of lymph collected from sites of sterile or pathogen-induced inflammation indicate the dynamic nature of the lymph protein content reflecting the presence of several tissue specific proteins [2]–[6], [9]–[15]. It was also found that lymph contains a soluble peptidome/degradome in the amounts higher than in serum [15]. These results led to the notion that lymph directly circulating in each parenchymal organ collects products derived from their metabolic/catabolic activity, forming an enriched lymph proteome reflecting the ongoing extracellular and intracellular processes in the host [16]. From this standpoint, lymph within the microenvironment of the LN is expected to contain unique proteomic information regarding LN responses to different pathologic conditions, including the infectious disease.

An early inflammatory response in LNs (lymphadenitis, LA) takes place in the pathogenesis of many infectious diseases, including but not limited to plague, anthrax, tularemia and tuberculosis. It is aimed at eliminating the pathogen. However, some pathogens are able to cause extensive damage of LNs. Treatment of bacterial LA represents a significant medical challenge because antibiotics penetrate the LN poorly and do not target the bacterial toxins and other pathogenic factors. However, understanding the mechanism of LA for the development of effective treatments has been hampered by a number of limitations, especially in small-rodent models. There is very limited information about the proteomic content of the LN microenvironment during LA [14], [17]–[19].

In anthrax, the lymphatic system serves as the conduit by which germinating *B. anthracis* spores are delivered by macrophages to the sentinel LNs within hours after exposure [20]. However, no specific treatment or prophylaxis of anthrax LA is available to complement the effect of commonly administered antibiotics. The complex molecular processes of how anthrax LA develops in the LN and contributes to remote organ dysfunction are not well understood. The specific protein environment of LN making it a niche for fast bacterial multiplication has not been characterized. It remains elusive as to which bacterial pathogenic factors are responsible for LA and which host mediators are involved. As the first step to address this knowledge gap in this study we revealed aberrations caused by anthrax infection in the lymph proteome in murine model of cutaneous infection using a label-free semi-quantitative mass spectrometry-based approach, and compared the results with the corresponding data on serum obtained in our previous study [21]. We demonstrate that the soluble protein fraction of LNs (intra-nodal lymph) contains a wealth of disease-specific information distinct from that in serum. Also, using specific antibodies and the reverse-phase microarray technique (RPMA) [22]–[29], we for the first time identify a number of phosphoproteins present in the intra-nodal lymph. Bioinformatic analysis of these data revealed novel information on the processes operating in the specific environment of lymphatics and their implication to the mechanism of anthrax pathogenicity.

## Materials and Methods

### Reagents and antibodies

Antibodies against total and phosphorylated forms of the following proteins used for reverse phase protein microarrays were from Cell Signaling Technology (Beverly, MA) unless otherwise noted and used at the dilutions indicated: 1∶50 for Chk1 (Ser345), Ras-GRF1 (Ser916), Stat5 (Tyr694), Caspase-7 cleaved (Asp198), p38 MAP Kinase (Thr180/Tyr182), caspase-3 cleaved (Asp175), e-NOS (Ser113), e-NOS (Ser1177), NF-kappaB p65 (Ser536), p70 S6 kinase (Ser371), p70 S6 kinase (Thr389), Ship1 (Tyr1020); 1∶100 for Bad, BLM, CD68, Chk2(Ser33/Ser35), LC3B, Lck (Tyr505) (Life Technologies, NY, USA), myeloperoxidase (Abcam), HSP27 (Ser82), Stat1, Stat3 (Tyr705), Stat3 (Ser727), CREB (Ser 133), AKT, AKT (Ser473), Bad, p53 (Ser15), Caspase-9 cleaved (Asp330), Stat6 (Tyr641), e-NOS, XIAP, Puma, FLIP; 1∶200 for ERK1/2, p38 MAP Kinase, PTEN (Ser380), Sta5 (3H7), PARP cleaved (D214), ERK1/2 (Thr202/Tyr204), Beclin 1, FADD (Ser194), Sumo1, S6 Ribosomal protein (Ser235/Ser236), Stat6; 1∶250 for Src (Tyr416), Src (Tyr527), GSK-3 alpha/beta (Ser21/9); 1∶500 for SAPK/JNK, PTEN, Bcl-xL, Survivin, SAPK/JNK(Thr183/Tyr185), HSP90, ATF (Thr69/Thr71), pIKBa; 1∶750 for Jak1 (Tyr1022); 1∶1000 for CAMKII, Caspase-8 (EMD Millipore, MA, USA), TLR9, INPP4 (Abcam), Stat1 (Tyr701), Stat3, Bax, Bim, S6 Ribosomal protein (Ser240/Ser244), Ship1 (Tyr1020); 1∶2000 for IL-10 (Abcam, MA, USA), Sumo2/3, Proteosome 20S; 1∶4000 for HMGA1. Other reagents were from Sigma-Aldrich (St Louis, MO).

### Animal challenge, extraction of proteins from LNs, and histopathological analysis of LNs

All animal procedures were approved by the George Mason University Institutional Animal Care and Use Committee (Protocol #284). All surgery was performed after carbon dioxide asphyxiation, and all efforts were made to minimize suffering. Male 6- to 8-week-old DBA/2J mice (Jackson Labs) received food and water *ad libitum* and were challenged with *B. anthracis* Sterne spores (4×10^6^ spores in 20 µl of PBS, intradermally into each hind footpad) on day 0. The strain is fully toxigenic but strongly attenuated due to the lack of a polypeptide capsule. Survival of animals was monitored for 4 days. Thirty min before euthanasia the animals were anesthetized with ketamine/xylazine and injected into foot pads with 20 µl of 1% tracer dye Evans Blue in PBS. Groups of mice were euthanized at daily intervals on days 1 to 3 post challenge. The popliteal LNs (two per animal at each time point) were surgically removed. One LN from each mouse was put into 10% neutral buffered formalin solution for histological evaluation, and another was used for soluble protein extraction using the following procedure. The LNs were trimmed of the surrounding tissue, dissected into multiple pieces with a razor blade, suspended in 100 µl of PBS containing protease inhibitors (Pierce) and finally spun at 10,000 g for 5 min to pellet tissue debris and bacteria. The supernatants containing extracted proteins were used for MS and RPMA analyses as described below. The pellet was re-suspended in PBS and used to determine a bacterial load by plating different dilutions of suspensions onto LB agar plates. The plates were incubated at 37°C overnight. The number of colonies grown reflected the presence of spores and vegetative bacterial cells. To determine the number of heat-resistant un-germinated spores the samples were incubated at 65°C for 30 min before plating.

After fixing in formalin, the tissues were embedded in paraffin, the paraffin blocks were sliced into 8 µm sections, and mounted onto glass slides for standard hematoxylene/eosine (H&E) staining and further microscopic evaluation. To analyze the presence of bacteria within the LNs, the slides were subjected to the procedure of antigen retrieval by incubating them for 20 min in citrate buffer (15 mM citric acid, pH 6.0) at 95°C. The slides were then stained with rabbit anti-*B. anthracis* immune serum (dilution 1∶100) followed by a fluorescein Alexa 488-labelled secondary goat anti-rabbit IgG antibody. Fluorescence was detected at 495/520 nm using Olympus BX51 microscope. The anti-*B.anthracis* serum was obtained from rabbits immunized with spores of the Sterne strain and was shown by us to recognize a vegetative form of the bacterium.

To detect the presence of neutrophils, sections after antigen retrieval were incubated in 3% hydrogen peroxide in methanol for 5 min to inhibit peroxidase activity, blocked with Dako Protein block (Dako) for 5 min, and then incubated with a primary anti-myeloperoxidase antibody (Ab9535 from AbCam, dilution 1∶50) for 30 min, followed by Dako anti-rabbit EnVision+ HRP-Labelled Polymer (Dako). Colorimetric detection was completed with diaminobenzidine for 5 min, and slides were counterstained with H&E.

### Mass spectrometry (MS) data acquisition and analysis

Groups of 4 mice (from the total of 20 challenged at day 0) were used at each time point post challenge (days 1 to 3). Only 2 mice survived at day 4, and one of them was also included in the analysis. Additionally, 4 naïve mice were used as controls. The soluble protein samples from individual mice corresponding to a particular time point were pooled, dried with SpeedVac, reconstituted in 8 M urea, reduced by 10 mM DTT for 30 min, alkylated by 50 mM iodoacetamide for 30 min, and digested by trypsin at 37°C overnight. Tryptic peptides were further purified by Zip-Tip (Millipore) and analyzed by LC-MS/MS using a linear ion-trap mass spectrometer (LTQ, Orbitrap). After sample injection, the column was washed for 5 min with mobile phase A (0.4% acetic acid) and peptides eluted using a linear gradient of 0% mobile phase B (0.4% acetic acid, 80% acetonitrile) to 50% mobile phase B in 30 min at 250 nl/min, then to 100% mobile phase B for an additional 5 min. The LTQ mass spectrometer was operated in a data-dependent mode in which each full MS scan was followed by five MS/MS scans where the five most abundant molecular ions were dynamically selected for collision-induced dissociation using a normalized collision energy of 35%.

Tandem mass spectra were collected by Xcalibur 2.0.2 and searched against the NCBI mouse protein database using SEQUEST (Bioworks 3.3.1 software from ThermoFisher) using tryptic cleavage constraints. Mass tolerance for precursor ions was 5 ppm and mass tolerance for fragment ions was 0.25 Da. SEQUEST filter criteria were: Xcorr *vs.* charge 1.9, 2.2, 3.5 for 1+, 2+, 3+ ions; maximum probability of randomized identification of peptide <0.01. The results were then evaluated manually and submitted to the ProteomeXchange Consortium (http://proteomecentral.proteomexchange.org) via the PRIDE partner repository with the dataset identifier PXD001342. Protein identifications and number of identifying spectra (peptide hits) for each sample were exported using >99% confidence limit for protein identification with peptides from a given protein identified at least in two independent samples obtained from naive mice or during infectious process. It needs to be noted that this MS-based proteomic approach identifies the protein-derived peptides and assigns them to the corresponding full-length proteins, but does not provide direct information on their proteolytic status. Therefore the MS list reflects presence of the full-length and fragmented proteins (peptidome).

The proteins identified at the particular day post challenge from LNs of naïve (day 0) and infected mice (days 1 to 4) were compiled in a list, and the total number of spectral hits per time point for each protein was normalized by a number of MS experiments. For a particular protein, this rank served as a crude measure for comparing changes in protein abundance similar to the approach used by Faca et al. (2008) for analysis of plasma proteins [30]. The authors found that the number of spectral hits for a given protein correlated significantly with its plasma concentration (R2 = 0.84). We previously evaluated a subset of 6 proteins from serum exhibiting profound shifts in the number of spectral hits upon infection [21]. The spectral hit trends for tested proteins correlated with 72±9 (CI) to 100% of western blot data with *α* = 0.05.

In order to increase reliability of protein identifications, as a preliminary condition, we excluded from consideration the proteins with single spectral hits which were unique among all tested samples. The protein was considered to be up- or down-regulated by infection based on the average number of hits in infected mice per day of infection in comparison with the number of hits corresponding tonaïve mice.

For annotation analysis, GI protein accession numbers were uploaded into the DAVID (Database for Annotation, Visualization and Integrated Discovery) informatics tool (DAVID Bioinformatics Resources 6.7 [31]). For GO Term (Gene Ontology) analysis we studied the Biological Process categories using the GO FAT default settings. Use of GO FAT generated more informative results than any specific GO term level. At these settings the program uses a subset of GO terms depleted of the broadest terms (primarily from the top 5 levels of the tree) to avoid overshadowing of the more specific terms (term specificity defined on the basis of the number of child terms in the hierarchy; see DAVID website). For functional annotation searches we set the following parameters: threshold count 3, EASE score (enrichment probability) 0.1; medium stringency for functional annotation clusters. For KEGG pathway searches the parameters were: threshold count 5 (minimal count of proteins mapped to the pathway ≥5), enrichment probability ≤0.05 (strong enrichment).

Enrichment values (for GO terms), enrichment scores (for annotation clusters), and statistical determinants (for *p* values and Benjamini coefficients) are those calculated by DAVID software. The Group Enrichment Score is a geometric mean (in -lg scale) of member's Fisher exact test *p* values in a corresponding annotation cluster, where each member’s *p* value reflects the probability of enrichment for a particular gene in a given gene list. The Benjamini coefficients are Benjamini-Hochberg-corrected *p* values adjusted for multiple comparisons to lower the family-wise false discovery rate and thus are more conservative than Fisher exact *p* values.

### RPMA analysis

Soluble protein samples from individual mice were analyzed separately in groups of three mice per time point. Three mice were used as uninfected controls. Each sample was mixed with the equal volume of 2x SDS-PAGE loading buffer supplemented with 12 mM DTT, 2x cocktail of protease inhibitors (Pierce), phosphatase inhibitors (100 mM sodium fluoride, 0.4 mM sodium vanadate), 4 mM EDTA, and finally boiled for 10 min before printing onto microarray slides. Three nl of each sample were arrayed by direct contact printing onto nitrocellulose slides (Whatman, MA) using a high-resolution 2470 arrayer (Aushon Biosystems, Billerica, MA). Samples were printed as duplicates of the four-point serial dilution curves to ensure the linear detection range for the antibody concentrations used. Slides were stored with desiccant (Drierite, W. A. Hammond, Xenia, OH, USA) at −20°C before analysis with antibodies. To estimate the total protein amount, selected slides were stained with Sypro Ruby Protein Blot Stain (Molecular Probes, Eugene, OR) and visualized on a Fluorchem imaging system (Alpha Innotech, San Leandro, CA) equipped with a Cy3 filter. Slides were stained with specific antibodies on an automated slide stainer (Dako, Carpinteria, CA) using a biotin-linked peroxidase-catalyzed signal amplification. The arrayed slides were placed into 1X Re-Blot solution (Chemicon, Temecula, CA) for 15 min, washed two times for 5 min each in PBS, placed into I-Block solution (Applied Biosystems, Foster City, CA) in PBS/0.1% Tween-20 for at least 2 h, and then immunostained using an automatic slide stainer (Autostainer, Dako Cytomation, Carpinteria, CA) using manufacturer-supplied reagents. Briefly, the slides were incubated for 5 min with hydrogen peroxide, rinsed with high-salt Tris-buffered saline (CSA Buffer, Dako) supplemented with 0.1% Tween-20, blocked with avidin block solution for 10 min, rinsed with CSA buffer, and then incubated with biotin block solution for 10 min. After another CSA buffer rinse, 5 min incubation with Protein Block solution was followed by air-drying. The slides were then incubated with either a specific primary antibody diluted in Dako Antibody Diluent or, as a control, with only DAKO Antibody Diluent for 30 min. Prior to use on the RPMA, every antibody underwent extensive validation for specificity (e.g., single band on western blot, peptide competition, ligand induction for phospho-specific reagents). The slides were then washed with CSA buffer and incubated with a secondary biotinylated goat anti-rabbit IgG H+L antibody (1∶10000) (Vector Labs, Burlingame, CA) for 15 min. For amplification purposes, the slides were washed with CSA buffer and incubated with streptavidin-horseradish peroxidase (HRP) for 15 min, followed by a CSA buffer rinse. Slides were then incubated for 5 min in diaminobenzidine (DAB) chromogen diluted in Dako DAB diluent, washed in deionized water and imaged using UMAX 2100XL flatbed scanner (UMAX, Dallas, TX) using the following settings: white balance 255, black 0, middle tone 1.37, 600 dpi, 14 bit.

Spot intensity was analyzed by Image Quant v5.2 software (Molecular Dynamics). Data reduction was performed with RPMA Analysis Suite (http://capmm.gmu.edu/rpma-analysis-suite). To normalize data between samples, the relative intensity value for each endpoint for each spot was divided by the relative intensity value for the total protein. The 95% confidence intervals (CI) for a given protein were calculated using the *t*-test for 3 independent samples at each time point. An average CI for all RPMA data as a characteristic of its variability between different proteins was found to be in the range from 7.0 to 9.8% (*p* 0.95).

## Results

### MS analysis of the soluble LN proteome in naïve and *B. anthracis*-challenged mice

**Figure 1:**
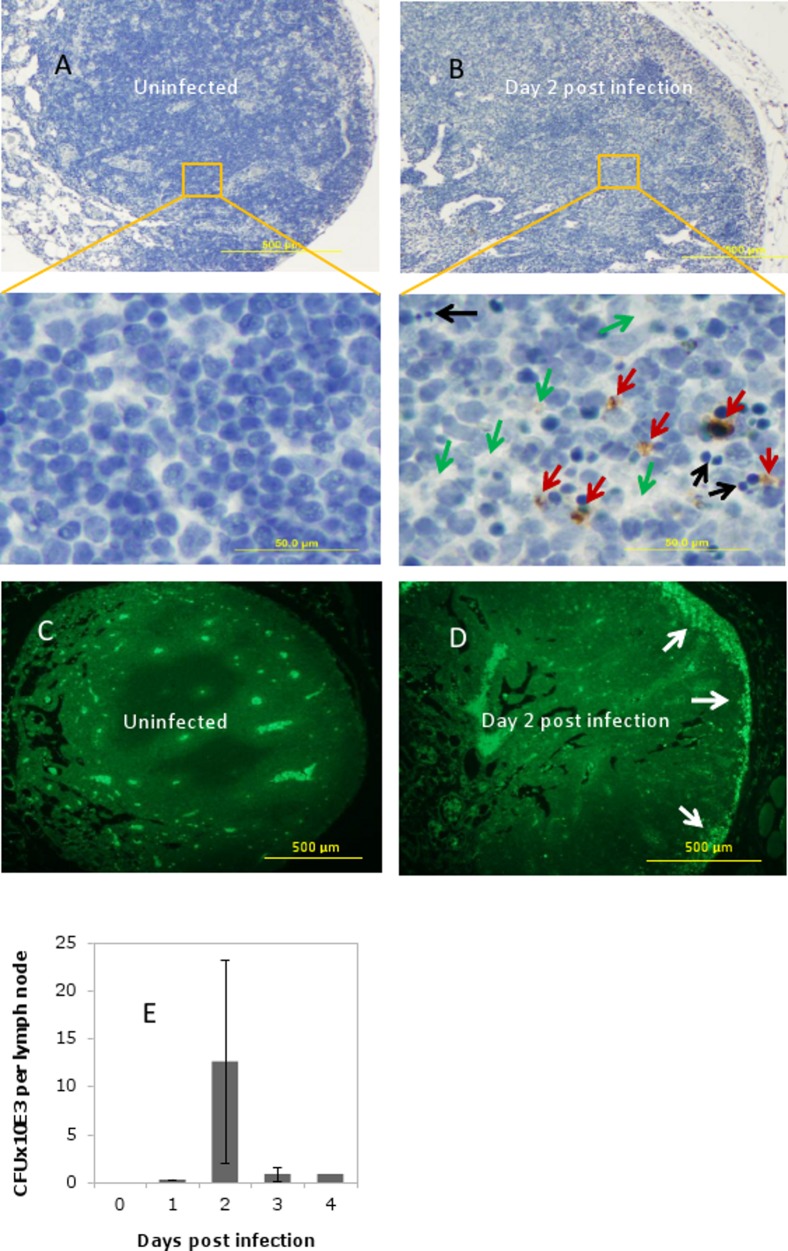
Analyses of popliteal LNs in *B. anthracis-*infected mice two days post challenge *vs.* uninfected mice. (A, B) H&E-stained sections of formalin-fixed LN tissue from uninfected (A) and infected (B) animals. Top panels show global views. Bottom panels show expanded views of regions identified by squares and demonstrate changes in the histopathology of cortical LN region. Tissue edema (green arrows) and numerous pyknotic cells (black arrows) are visible. Infiltrating neutrophils immunostained brown for myeloperoxidase (red arrows) are present. (C, D) Sections of formalin-fixed LN tissue from infected (D) and uninfected (C) mice immunostaned with rabbit anti-*B. anthracis* serum followed by secondary fluorescently-labelled antibody show accumulation of a large number of green-fluorescent bacteria (arrows) in the subcapsular region of LN of infected mouse. Fluorescence was detected at 495/520 nm using Olympus BX51 microscope. No increased subcapsular staining was found in control animals. The pictures represent typical observation obtained from ≥3 infected mice. (E) Bacterial counts in LNs of infected mice after plating of the homogenized tissue onto LB agar. Error bars represent SD of mean (*n* = 3 for days 0 to 3; *n* = 1 for day 4).

To characterize proteome of lymph during anthrax infection in comparison with naïve mice we carried out two independent challenge experiments using 20 animals. Additionally, four naïve mice served as uninfected controls. Animal were injected with 4 million of *B. anthracis* Sterne spores into each hind footpad and were observed for four days. In this model the infectious anthrax spores from the site of infection become delivered to the popliteal LNs and give rise to the vegetative bacteria which ultimately disseminate to other organs. The infection material in LNs on day 1 post infection contained heat-resistant spores in the amount of 153+/−128 (SD) colony-forming units (cfu) per organ determined by seeding the tissue homogenates onto LB-agar plates after incubation at 65°C for 30 min. The spore count was reduced to 86+/−61(SD) cfu on the day 2 post infection. The peak of disease judged by the strong inflammatory response in footpads and onset of mortality took place at days 2 and 3 post infection. The animals which did not die after day 3 demonstrated a reduced footpad swelling and decreased bacterial counts indicating their recovery from infection. Histological assessment of LN tissue stained with hematoxylene/eosine (H&E) in infected mice in comparison with unchallenged controls revealed tissue edema, infiltration by inflammatory neutrophils, and dying cells with pyknotic appearance (Fig. 1A, B). The bacterial clusters were visible in the subcapsular region of LNs (Fig. 1C, D) at day 2 post infection, coinciding with massive bacterial proliferation (Fig. 1E).

The popliteal and inguinal LNs of four control naïve mice were visualized by injection of 1% Evans Blue dye into the hind footpads. After 30 min mice were euthanized, LNs were surgically removed and put on ice. The soluble content of each LN was extracted with PBS containing protease inhibitors after mincing the LN tissue with a razor blade into multiple pieces. The tissue debris was removed by centrifugation and the supernatants were processed for MS analysis as described in Materials and Methods. The LC-MS/MS experiments using four independent samples from different mice detected peptides from 302, 366, 376, and 386 proteins. At the next step, in order to increase reliability of protein identifications, as a self-imposed restriction we excluded from consideration 160 proteins with a single peptide spectral hit which were unique among all four samples. The rest of the 380 proteins identified in one or more samples at least twice were used to generate a list of naïve lymph proteins including the protein identity, the average total number of spectral hits per sample (AV), standard deviations (SD) among samples as well as the 99% confidence intervals (CI) (Table S1). When ranked based on the expected relative variability (CI/AV), the data show that about 250 proteins are expected to be present in every LC-MS/MS run with 99% confidence. The most variable spectral appearance (CI/AV>1) was mainly found in the case of 120 rare proteins with two or less spectral hits. The soluble content of naïve LNs was dominated by peptides derived from serum albumin, with the next most abundant proteins being creatine kinase M-type, parvalbumin alpha, fatty acid synthase, serotransferrin precursor, fatty acid-binding protein (adipocyte), and glyceraldehyde-3-phosphate dehydrogenase. These results defined a background soluble proteomic LN content for a comparative analysis with anthrax infection.

**Figure 2:**
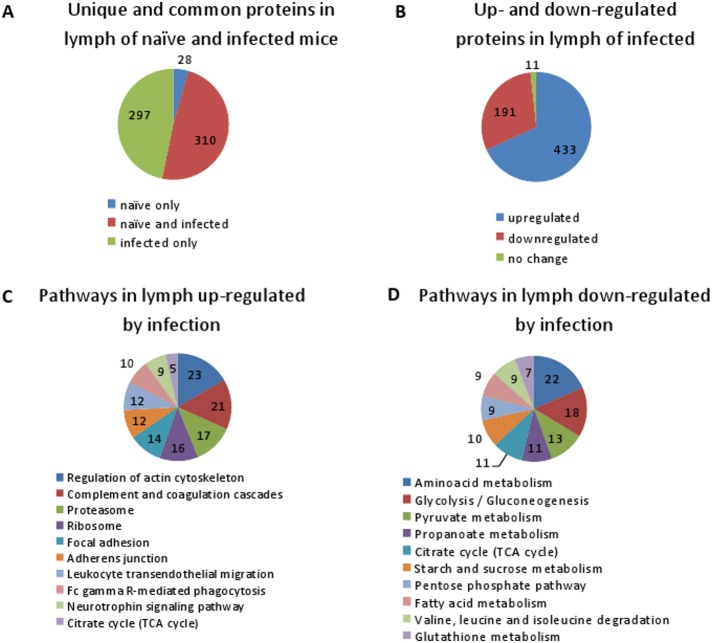
Pie charts of the protein content (A, B) and top-scoring processes (C, D) in LNs of naïve and *B. anthracis*-infected mice. (A) LN soluble proteome of spore-challenged mice contains a large number of proteins which appeared in LNs of during infection but were undetectable in naïve mice. (B) Infection up- or down-regulated a majority of LN proteins. Only 11 proteins showed no change in abundance. (C, D) KEGG analysis revealed cellular pathways relevant to LN proteins which were up-regulated (C) or down-regulated (D) by infection. Figures in the charts indicate the number of proteins in a particular category or pathway.

During the infectious process the popliteal LNs from individual mice were surgically removed at daily intervals and their content extracted as described above. The extracts from different mice corresponding to the same time point were pooled and analyzed by LC-MS/MS. Overall the MS identifications in control and infected mice at four time points resulted in 760 different proteins. The total list of proteins was sorted according to the average number of hits for a particular protein per day of infection, and the proteins demonstrating unique peptide hits were excluded from further consideration. This procedure resulted in the final list of 635 proteins. Among these, 310 proteins belonged to both naïve and infected mice while 28 proteins were found in the naive mice only. The infectious process induced 297 unique proteins. From the final list, 433 proteins were up-regulated and 191 proteins down-regulated in the infected animals in comparison with uninfected controls (Fig. 2 and Table S2).

### DAVID Functional Annotation analysis

We analyzed the lists of up- and down-regulated proteins using Database for Annotation, Visualization and Integrated Discovery (DAVID). The software algorithm measures relationships among the annotation terms based on the degree of commonality in gene content between two annotations to classify the groups of similar annotation. The more common genes annotations share, the higher chance they will be grouped together. Each annotation group (cluster) is assigned a Group Enrichment Score as the geometric mean (in -log scale) of members’ *p* values which is used to rank their biological significance. Thus, the top-ranked annotation groups most likely have consistent lower *p* values for their annotation members. Grouping genes based on functional similarity can systematically enhance biological interpretation of large lists of genes derived from high throughput studies.

The DAVID Functional Classification tool generates a gene-to-gene similarity matrix-based shared functional annotation using over 75,000 terms reflecting biological processes from 14 functional annotation sources. From the list of up-regulated proteins the software identified 424 matching entrees in its database and categorized them into enriched processes reflecting relative contextual abundance of the proteins in the analyzed list relative to the genome-wide gene list used by DAVID as a background. In total, 208 biological processes from GO FAT database were identified. Table 1 shows some of the processes with the highest enrichment associated with the up-regulated proteins after inspection and removal of the redundant processes as well as the GO terms with the statistical reliability <95%.

**Table 1:** DAVID-based analysis of enriched processes for proteins up-regulated in infection.

	GO term for enriched process	Count*	Fold Enrichment**
1	GO:0006957∼complement activation, alternative pathway	5	19.2
2	GO:0050818∼regulation of coagulation	6	11.5
3	GO:0032956∼regulation of actin cytoskeleton organization	16	10.2
4	GO:0042743∼hydrogen peroxide metabolic process	5	9.6
5	GO:0007599∼hemostasis	17	9.2
6	GO:0002526∼acute inflammatory response	19	9
7	GO:0043254∼regulation of protein complex assembly	14	8.3
8	GO:0031099∼regeneration	5	8.3
9	GO:0006418∼tRNA aminoacylation for protein translation	9	7.5
10	GO:0006956∼complement activation	7	7.5
11	GO:0050878∼regulation of body fluid levels	17	7.3
12	GO:0034097∼response to cytokine stimulus	6	7.2
13	GO:0042311∼vasodilation	5	7.1
14	GO:0006879∼cellular iron ion homeostasis	5	6.6
15	GO:0044087∼regulation of cellular component biogenesis	15	6.5
16	GO:0065004∼protein-DNA complex assembly	12	6.1
17	GO:0006007∼glucose catabolic process	8	5.9
18	GO:0051494∼negative regulation of cytoskeleton organization	7	5.6
19	GO:0050880∼regulation of blood vessel size	7	5.6
20	GO:0034622∼cellular macromolecular complex assembly	31	5.5
21	GO:0003018∼vascular process in circulatory system	7	5.4
22	GO:0000302∼response to reactive oxygen species	5	5.3
23	GO:0033043∼regulation of organelle organization	21	5.2
24	GO:0006959∼humoral immune response	7	5
25	GO:0006323∼DNA packaging	12	4.6
26	GO:0009611∼response to wounding	41	4.5
27	GO:0006954∼inflammatory response	24	4.1
28	GO:0045087∼innate immune response	11	3.9
29	GO:0051604∼protein maturation	9	3.6
30	GO:0006461∼protein complex assembly	20	3.4
31	GO:0051129∼negative regulation of cellular component organization	8	3.3
32	GO:0010564∼regulation of cell cycle process	6	3.2
33	GO:0006399∼tRNA metabolic process	9	3.1
34	GO:0006979∼response to oxidative stress	7	3.1
35	GO:0009719∼response to endogenous stimulus	14	2.9
36	GO:0005996∼monosaccharide metabolic process	11	2.2
37	GO:0019725∼cellular homeostasis	20	2.2
38	GO:0010033∼response to organic substance	28	2.1
39	GO:0042592∼homeostatic process	28	1.8
40	GO:0006508∼proteolysis	38	1.4

*Count refers to a number of genes identified in a corresponding term. Terms with less than 5 genes identified were excluded from analysis.

**Enrichment value calculated by DAVID is a measure of probability (in –log scale) for members of a particular annotation cluster to be grouped together (for further explanation see Materials and Methods).

We then used the clustering algorithm to categorize these processes into functionally related groups. The software generated 68 clusters. The selected non-redundant ones are shown in Table S3. The highest enrichment score of >10 was assigned to the cluster of GO terms including the response to wounding, hemostasis, coagulation, regulation of body fluid levels, inflammatory response, and defense response. Relevant to these terms is the clustered group with the score of 2.7 including regulation of coagulation, acute inflammatory response, complement activation (alternative and classical pathways), innate immune response, humoral immune response, protein maturation by peptide bond cleavage, and leukocyte mediated immunity. Vascular disturbance is reflected by the group including regulation of blood vessel size, vascular process in circulatory system, vasodilation, and circulatory system processes (score 1.7). All groups of processes mentioned above bear close relevance to the pathogenic features described in anthrax infection previously [32].

Highly enriched are also three groups of processes comprising more than 30 proteins (scores 9.0, 6.4, and 6.1) including broad terms of cellular macromolecular complex assembly, protein complex assembly, cellular macromolecular complex subunit organization, nucleosome and chromatin assembly, and regulation of actin cytoskeleton organization. The response to cytokine, hormone, and peptide hormone stimuli group encompasses about 30 proteins with the score of 2.4. Cellular and ion homeostatic processes cluster is also abundant (28 proteins). The proteolysis cluster includes 38 terms consistent with the prominent role of proteases in anthrax pathology [32]–[38]. At the level of metabolism, enrichment takes place for the monosaccharide and glucose catabolic processes (score of 2.6) indicating the importance of glycolysis in the hypoxic environment of lymphatics.

The 188 proteins down-regulated during infection were grouped into 28 clusters. Representative examples are shown in Table S4. The processes almost exclusively belong to cell metabolism of carbohydrates, alcohols, lipids, glycolysis, energy derivation by oxidation of organic compounds and aerobic respiration, catabolism of tricarboxylic and other organic acids. The glycolytic processes appear to be a subject of both up- and down-regulation through different sets of genes. The notable metabolic changes also include reduced production of nucleotides and ATP, cytoskeletal processes as well as regulation of cellular homeostasis.

The Kyoto Encyclopedia of Genes and Genomes (KEGG) pathway analysis is widely used to map proteins onto known pathways from the KEGG database. We used the lists of up- and down-regulated proteins to generate KEGG pathway maps with a stringent *p* value ≥0.05 and a minimal number of proteins mapped to the pathway ≥5 in order to focus on the most prominent pathways. The up-regulated proteins were mapped to 15 pathways. The majority of the proteins belong to the pathways reflecting closely related processes involved in anti-microbial defenses (complement and coagulation cascades, leukocyte transendothelial migration, regulation of actin cytoskeleton, Fc gamma R-mediated phagocytosis, antigen processing and presentation) (Fig. 2 and Table S5). Two pathways including cytoplasmic ribosome and proteasome structural proteins are consistent with the cell damage, while two pathways of focal adhesion and adherens junction signaling indicate perturbations in the cell-cell and cell-matrix interactions. Cellular metabolism pathways included Krebbs’ cycle, glycolysis, amino acids, and fatty acids.

**Figure 3:**
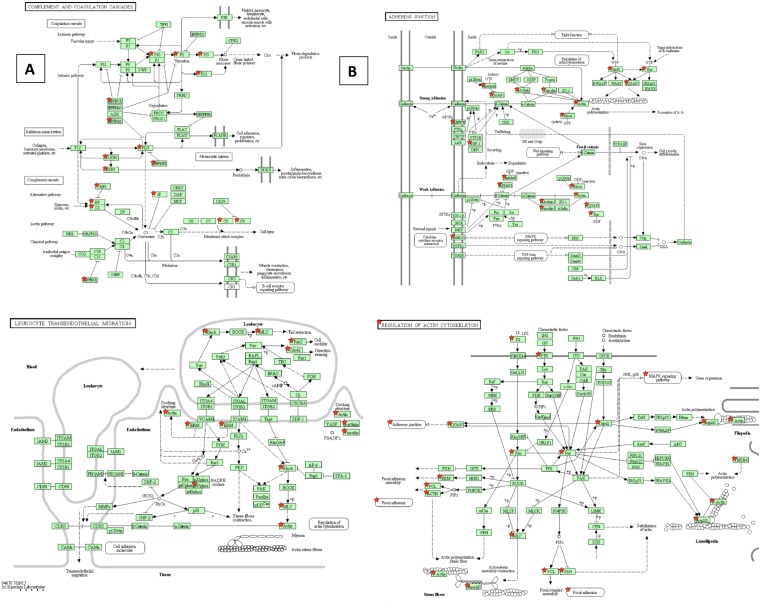
KEGG maps illustrating selected pathways relevant to the soluble protein content of LNs in *B. anthracis*-infected mice. Lists of proteins up- or down-regulated in LNs during infection (Table S2) were used as an input for KEGG software, which contains a database of pathway maps reflecting different biological processes. Red stars indicate the LN proteins mapped to the corresponding pathway using the following parameters: Fisher exact *p* value for enrichment probability ≤0.05 (strongly enriched), and a minimal number of proteins mapped to the pathway ≥5.

The down-regulated pathways were dominated by metabolic processes indicating a broad shutdown of main cellular functions belonging to glycolysis, citrate cycle, metabolism of pyruvate, propanoate, fatty acids, amino acids, purines and degradation of xenobiotics (Fig. 2 and Table S6). Several genes involved in control of glycogenesis and lipid metabolism were also mapped to the insulin and peroxisome proliferator-activated receptors (PPAR) signaling pathways. The latter is relevant to regulating fundamental aspects of cellular activation, proliferation, and differentiation [39]. PPAR-gamma is a negative regulator of neutrophil migration. It not only stimulates transcription of certain target genes involved in adipocyte differentiation and glucose metabolism but also downregulates activity of key proinflammatory transcription factors, including NF-kappaB and signal transducer and activator of transcription 6 (STAT6). Diagrams of the most prominent pathways indicating proteins from the LN proteome of infected mice mapped to the pathways as a result of KEGG analysis are shown in Fig. 3.

We previously analyzed the naïve mouse serum proteome using the same MS experimental technique and strain of mice as in the current study [21]. To determine if serum proteins can be found in the soluble LN proteome of naïve or spore-challenged mice, we compared the serum and lymph MS data. The serum list contained 224 proteins selected from the total list based on the average frequency of >1 peptide hit per sample in order to exclude from consideration the low-abundance proteins (Table S7). Side-by-side comparison with the lymph data from Table S2 containing proteins from naïve and spore-challenged mice resulted in the identification of 83 common entries (Table 2).

**Table 2:** Proteins from naïve and *B. anthracis-*infected mice common between serum and lymph ranked according to their abundance in lymph during infection.

	Protein	GI number	Average spectral hits		
			Naïve serum*	Naïve lymph**	Infected lymph***
1	albumin	148673375	457.5	214.5	375.75
2	transferrin	20330802	166	55	78.625
3	serine (or cysteine) proteinase inhibitor,clade A, member 3K	15079234	51	24.5	53.625
4	parvalbumin	31980767	1.5	57	47.5
5	complement component 3	28175786	177.5	16.5	33.375
6	hemopexin	18044757	28	21	26.25
7	serine (or cysteine) proteinase inhibitor,clade A, member 1b	76881807	19	12.5	18.25
8	apolipoprotein A-I	2145141	82	8.5	17.75
9	plasminogen	200403	8	3	17.625
10	hemoglobin, beta adult major chain	31982300	311	11.5	16.5
11	vitamin D-binding protein	51172612	28	7.5	13
12	adenylate kinase 1	10946936	2.5	21	11.875
13	haptoglobin	8850219	8	0	9.75
14	alpha-2-HS-glycoprotein	7304875	30	6	9.625
15	alpha-1-antitrypsin 1–1 isoform 1 precursor	6678079	1.5	4	9.375
16	murinoglobulin 1	31982171	16	12	8.5
17	esterase 1	553910	17.5	4	7.25
18	coagulation factor II	6753798	16.5	0	6
19	complement factor B	218156291	8	1.5	5.5
20	inter alpha-trypsin inhibitor,heavy chain 4	226531047	13.5	1.5	5.5
21	hemoglobin alpha 1 chain	145301578	215	0.5	5.25
22	apolipoprotein E	20381029	24	2	5.125
23	CAP, adenylate cyclase-associated protein 1	157951604	13.5	0	4
24	fatty acid binding protein 4, adipocyte	14149635	3	0	3.875
25	glyoxalase 1	148676704	1.5	2	3.625
26	coactosin-like 1	19482160	2	1.5	3.125
27	serine (or cysteine) proteinase inhibitor, clade C (antithrombin), member 1	18252782	27	1	2.625
28	hypothetical protein LOC71775	21313642	7.5	0	2.625
29	peroxiredoxin 5 precursor	6755114	5	0	2.5
30	Rho, GDP dissociation inhibitor (GDI) beta	33563236	2	0	2.5
31	kininogen 1	12963497	16.5	0	2.375
32	serum amyloid A 1	6677843	2	0.5	2.25
33	histidine-rich glycoprotein	11066003	14	0	2
34	gelsolin	28916693	14	0	1.875
35	peptidylprolyl isomerase A	6679439	2.5	0	1.5
36	cell division cycle 42 homolog	6753364	1.5	0	1.5
37	serine (or cysteine) proteinase inhibitor,clade A, member 6	6680856	11.5	0	1.375
38	UMP-CMP kinase	165377065	1	6	1.375
39	vitronectin	6755987	10	0	1.25
40	actin related protein 2/3complex, subunit 3	9790141	4	0	1.25
41	peroxiredoxin 2	148747558	34	0.5	1.25
42	kininogen 2	41235784	5	0.5	1.25
43	inter-alpha trypsin inhibitor,heavy chain 2	226874935	1.5	0	1.125
44	paraoxonase 1	15215219	17	0	1.125
45	myosin light chain, regulatory B-like	71037403	2.5	1	1.125
46	destrin	9790219	5	0	1
47	selenoprotein P precursor	74271806	1.5	0	1
48	glutathione peroxidase 1	84871986	7.5	0.5	1
49	serine (or cysteine) proteinaseinhibitor, clade A, member 3G	86476056	8.5	0	0.875
50	complement component 9	15375312	7	0	0.75
51	phosphatidylethanolaminebinding protein 1	84794552	2	1	0.75
52	alpha 1 microglobulin/bikunin	6680684	0.5	0.5	0.625
53	serum amyloid P-component	226958497	6.5	0	0.625
54	hemoglobin, beta adult minor chain	17647499	200	0.5	0.625
55	myoglobin	21359820	15.5	0	0.625
56	acid phosphatase 1, soluble	31542070	1	0.5	0.625
57	fibrinogen, alpha polypeptide	33563252	7	0	0.625
58	nucleoside-diphosphate kinase 1	37700232	6.5	0	0.625
59	carboxypeptidase N,polypeptide 2 homolog	147904569	8	0	0.625
60	peroxiredoxin 1	6754976	1.5	0.5	0.5
61	alpha-2-glycoprotein 1, zinc	148687277	7.5	0	0.5
62	SH3 domain bindingglutamic acid-richprotein-like 3	18017602	1.5	0	0.5
63	acylphosphatase 2, muscle type	27229219	1.5	0.5	0.5
64	beta-2-microglobulin	31981890	7	0	0.5
65	cofilin 1, non-muscle	6680924	22.5	0.5	0.375
66	calponin 2	6680952	3.5	0	0.375
67	cysteine and glycine-richprotein 1	6681069	1	0.5	0.375
68	alpha-crystallin B chain	6753530	1.5	0	0.375
69	proteosome (prosome, macropain)subunit, beta type 8(large multifunctional protease 7)	158303322	3.5	0	0.375
70	thioredoxin 1	6755911	3.5	0.5	0.375
71	apolipoprotein A-II	157951676	16.5	4.5	0.375
72	lysozyme	8393739	1.5	0	0.375
73	apolipoprotein C-III	15421856	118	0.5	0.375
74	ADP-ribosylation factor 3	6680718	2.5	0	0.25
75	ADP-ribosylation factor 5	6680722	1	0	0.25
76	macrophage migration inhibitory factor	6754696	2	0	0.25
77	adipsin	7304867	1.5	0.5	0.25
78	protease, serine, 1	16716569	15.5	0.5	0.25
79	proteasome(prosome, macropain)subunit, beta type 2	148698332	4.5	0	0.25
80	superoxide dismutase 1, soluble	45597447	2	0	0.25
81	serum amyloid A 4	6755398	31.5	0	0.25
82	fatty acid bindingprotein 5, epidermal	6754450	3	0.5	0.125
83	magnesium-dependentphosphatase-1	12963663	1	0.5	0

*Average from 3 mice; **Average from 4 mice; ***Average per day for LN samples collected at days 1 to 4 post challenge (4 mice per sample at days 1 to 3, one sample at day 4).

### Phosphoprotein content of lymph proteome

Protein phosphorylation is one of the most prominent and intensively studied post-translational modifications in biological systems. Dynamic phosphorylation of proteins on serine, threonine and tyrosine residues is recognized as a key mode of regulating cell cycle, cell growth, cell differentiation and metabolism [40]. The critical role of phosphoprotein-mediated intracellular signaling is widely recognized in the cancer field [41]. Less is known about the roles of phosphoprotein signaling during pathogenesis of infectious disease. Recently, our attention was attracted to phosphoprotein signaling pathways in connection with the homeostatic disturbances caused by pathogens in the host cells [24], [42]–[44]. In addition to the commonly studied intracellular signaling phosphoproteins, it has been recently recognized that the plasma and lymph also contain circulating soluble phosphoproteins [2], [4], [46]. However, the spectrum of phosphoproteins in lymph and plasma remains poorly characterized and the mechanism of their appearance in the extracellular fluids is not understood. One of the factors contributing to this situation in small animals was a sample size and a sensitivity of the analytical procedures.

In this study we overcame these limitations by using a reverse-phase protein microarray (RPMA) [22], [26], [29]. The assay is a miniature version of the dot blot utilizing specific antibodies against proteins of interest in the samples printed onto the surface of nitrocellulose slide. To ensure specific recognition of the antigen, the antibodies underwent process of validation by western blot of cellular lysates. In our analysis we used 71 validated antibodies against total and phosphorylated forms as well as other proteins relevant to cell signaling in order to characterize changes in their levels during the course of anthrax infection. Table 3 shows the up- and down-regulated proteins grouped according to their functional properties such as transcriptional response, mitogen-activated kinase signaling, apoptosis and autophagy, lipid signaling and PI3K/AKT, tyrosine kinase signaling. The numbers represent relative protein abundances in infected *vs.* uninfected LNs after normalization to the total amount of protein.

**Table 3:** Relative abundance of phosphoproteins and related signaling proteins in lymph of infected mice in comparison with naïve mice.

Up-regulated**					Down-regulated**				
Protein	Day1*	Day2*	Day3*	Aver-ageper day	Protein	Day1*	Day2*	Day3*	Aver-ageper day
**Transcriptional response**									
Stat1(Tyr701)	2.19	3.72	6.82	4.24	Stat6(Tyr641)	0.86	0.75	0.70	0.77
Stat3(Tyr705)	3.13	4.53	4.94	4.20	Jak1(Tyr1022/Tyr1023)	1.04	0.73	0.50	0.76
Stat1	1.82	2.62	3.15	2.53	ATF2(Thr69/Thr71)	0.68	0.57	0.56	0.61
Stat3(Ser727)	1.20	1.99	2.08	1.76	HMGA1	0.70	0.39	0.18	0.42
CREB(Ser133)	1.07	1.77	1.91	1.59					
IkappaB alpha	1.33	1.58	1.54	1.48					
Stat6	1.25	1.67	1.36	1.43					
NF-kappaB p65(Ser536)	1.18	1.40	1.30	1.29					
Stat5(Tyr694)	1.07	1.38	1.33	1.26					
Stat5	1.17	1.40	1.14	1.23					
**Mitogen-activated kinases**									
S6 Ribosomal Protein (Ser240/Ser244)	1.67	3.13	2.67	2.49	p70 S6 Kinase (Thr389)	1.00	0.84	0.93	0.92
S6 Ribosomal Protein (Ser235/Ser236)	1.58	3.00	2.32	2.30	SAPK/JNK(Thr183/Tyr185)	0.48	0.28	0.28	0.35
ERK 1/2	1.50	1.96	2.10	1.85	ERK1/2(Thr202/Tyr204)	0.49	0.31	0.19	0.33
Proteosome 20S	1.36	1.90	2.08	1.78	p38 MAP Kinase (Thr180/Tyr182)	0.42	0.13	0.19	0.25
p38 MAP Kinase	1.03	1.45	1.44	1.31	HSP27(Ser82)	0.59	0.38	0.29	0.42
p70 S6 Kinase (Ser371)	1.02	1.27	1.50	1.27					
**Apoptosis, autophagy, death receptor**									
Caspase 7 cleaved (Asp198)	1.49	2.16	2.85	2.17	Bim	0.80	0.96	0.89	0.88
Survivin	1.58	2.24	2.41	2.08	XIAP	0.58	0.37	0.45	0.47
PARP cleaved (Asp214)	1.49	1.67	2.05	1.74	TNF R1	0.60	0.33	1.22	0.72
Caspase 3 cleaved (Asp175)	1.41	1.77	1.73	1.64					
Beclin1	1.35	1.66	1.76	1.59					
LC3B	1.43	1.69	1.62	1.58					
Bad	1.11	1.81	1.48	1.47					
Bax	1.20	1.50	1.34	1.34					
Caspase 9 cleaved (Asp330)	1.07	1.22	1.49	1.26					
Puma	1.19	1.32	1.24	1.25					
FADD(Ser194)	0.95	1.16	1.36	1.16					
Bcl-xL	0.94	1.44	1.04	1.14					
Caspase 8	1.15	1.21	0.94	1.10					
**Lipid signaling/PI3K/AKT**									
AKT	1.26	1.83	1.80	1.63	Akt(Ser473)	0.99	0.92	0.40	0.77
SHP1(Tyr1020)	1.58	1.75	1.86	1.73	GSK3 alpha/beta (Ser21/Ser9)	1.05	0.95	0.80	0.93
INPP4	1.74	2.39	2.32	2.15					
PTEN	1.23	1.73	1.75	1.57					
**Other protein kinases**									
Src(Tyr527)	1.02	1.34	0.90	1.09	Src(Tyr416)	0.65	0.58	0.34	0.52
Lck(Tyr505)	1.28	1.17	1.36	1.27					
**Other**									
Myeloperoxidase	1.20	1.57	1.51	1.42	TLR9	0.85	0.90	0.75	0.83
CD68	1.23	1.32	1.14	1.23	Ras-GRF1(Ser916)	0.72	0.74	0.44	0.63
Sumo 1	1.10	1.36	1.15	1.20	eNOS(Ser1177)	0.76	0.72	0.55	0.68
Sumo 2/3	1.10	1.25	1.51	1.29					
IL-10	1.07	1.09	0.99	1.05					

*Mean protein abundance in infected relative to uninfected mice. Protein extracts from 3 individual LNs were analyzed at each time point.

**Deviation of mean (95% confidence interval) calculated across all proteins in the Table is ≤0.098. The proteins demonstrating changes of mean less than 0.098 were: SAPK/JNK, Bad, FLIP, eNOS(Ser113), IL-10, HSP90, p53 (Ser115).

**Figure 4:**
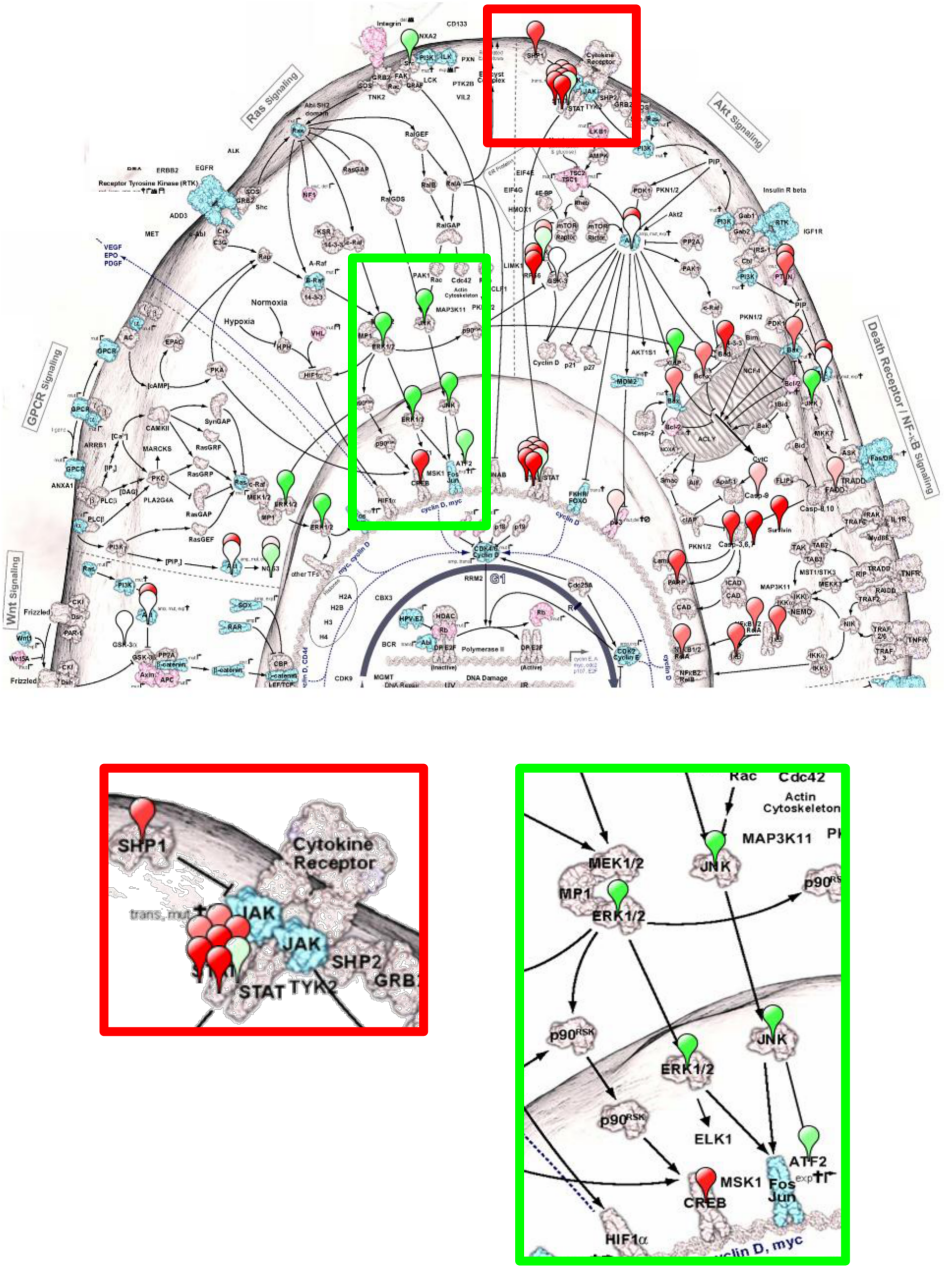
Cancer Landscape (Cscape) Protein Pathway Activation map of lymph from popliteal LN of mice infected with *B. anthracis* at day 2 post infection based on RPMA data. Increase and decrease in signaling are shown in increasing shades of red or green respectively. Each balloon pin is placed over the protein measured. Magnified view of the ERK and JNK in Ras pathway (green box) and STAT in cytokine signaling pathway (red box) are shown to reveal pathway details.

As one of the approaches to evaluate the results and reveal relevance of the tested proteins to known signaling networks, we utilized a novel pathway visualization CScape tool allowing map the activation changes reflected in the RPMA data onto a network image [47]. Figure 4 provides example of a signaling map view corresponding to the acute phase of infection at day 2 post challenge. The balloon pins are placed over the proteins measured, and the abundance levels are indicated by shades of green and red. The down-regulated protein can be mapped to TGF-beta, Wnt, GPCR, Ras and AKT signaling, while the up-regulated one belong to the death receptor, apoptosis and NF-kB pathways. Among the most up-regulated proteins are transcriptional factors including Stat1, 3 and CREB.

## Discussion

The major goal of this study was to explore the hypothesis that lymph within the microenvironment of LN contains unique proteomic information regarding the LN responses to different pathologic conditions including infectious disease. Using the murine model of cutaneous anthrax we also wanted to reconcile the proteomic data with known mechanistic features of infection by *B. anthracis*. We first characterized the soluble proteome of mouse popliteal LNs extracted from the dissected tissue in mild conditions using PBS. Several previous studies attempted characterization of lymph proteome. In one of the earlier reports Leak et al. examined the protein expression profiles of normal ovine lymph the thoracic duct lymph and identified 18 proteins [2]. Mittal et al reported a content of rodent lymph and its response to hemorrhagic shock and acute pancreatitis [6], [11]–[13]. Proteomic analysis of afferent pre-nodal lymph and matched plasma samples derived from healthy donors by Clement et al [48] revealed 253 proteins. Yuan et al [49] identified 77 mesenteric lymph proteins. One of the most comprehensive studies by Dzieciatkowska et al [50] identified a total of 477 human proteins from the 11 subjects' lymph samples. However, there are no studies on the analyses of the lymph proteins in the murine system. Here we report a list of 380 proteins obtained directly from popliteal LNs of naïve mice identified with >99% confidence (Table S1).

We carried out a side-by-side comparison the proteins found in naïve and B. anthracis-infected mice and ranked them based on the number of spectral hits (Table S2). In agreement with previous studies of human, ovine and rat lymph the soluble proteins from normal LNs contained (besides the overwhelming amount of albumin) the complement activation and blood coagulation components (fibrinogen), transporters (transferrin, apolipoproteins), protease inhibitors, acute phase response (haptoglobin, serum amyloid A1) as well as macroglobulins, products derived from the extracellular matrix and cellular catabolism and an array of other proteins. Remarkably, the infectious process resulted in the profound changes of the protein abundances and the appearance of a large number of proteins undetectable in the samples from naïve mice. These results demonstrate that the soluble LN proteins are sensitive indicators of the processes within the LNs during infection. Previous analysis of the lymph indicated the presence of processed peptides derived from cellular cytosolic and organelle proteins, extracellular proteins, and plasma membrane-associated proteins [15]. The proteome/peptidome of the lymph was shown to contain not only proteins derived from plasma proteins, but also proteins and peptides derived from the metabolism/catabolism of parenchymal organs, connective tissue remodeling, and secretion products derived from cells circulating in the lymph. The lymphatic endothelium was also shown to contribute to an array of proteins and cytokines in the lymph [16], [48].

Analyses of the data using DAVID functional annotation revealed the highly enriched proteins among the ones up-regulated in infected mice. These proteins form the top-scoring clusters of processes relevant to response to wounding, hemostasis/coagulation as well as regulation of body fluids followed by inflammatory responses and changes in cellular macromolecular complexes, including the cytoskeletal perturbations (Table 1 and Table S3). Among the most up-regulated proteins the proteins such as complement component 3, clade-A serine proteinase inhibitor, apolipoprotein A1, plasminogen and haptoglobin are known modulators of the innate immune response. The results also indicate that the acute innate response of the LNs to anthrax infection is accompanied by cellular damage and proinflammatory activity similar to the biological activity of intestinal (mesenteric) lymph after major trauma or episodes of shock [50]–[53]. Mesenteric lymph is strongly implicated in post-injury multiple organ failure (MOF) where gut-derived factors present in lymph serve as the triggers that initiate systemic inflammation and tissue injury. Acute lung injury and circulatory shock are typical features of MOF, followed by renal and hepatic dysfunction [51]. These clinical manifestations are characteristic of the pre-mortal condition seen in anthrax patients [54], thus implicating the bioactivity of lymph as a contributor to anthrax pathogenesis.

The damage is evident in the increased levels of normally intracellular proteins such as lactate dehydrogenases, actin, creatine kinases and other mitochondrial proteins, fatty acid-binding protein 4, and hemoglobin. Although the presence of hemoglobin might indicate a certain level of sample contamination from the lymph node blood vessels upon dissection of tissue, it can be ruled out based on the increased levels of this protein during infection. According to [50], a significant increase of hemolysis products such as hemoglobin may represent a common factor resulting from multiple pathways such as oxidative stress, nitric oxide (NO) depletion, and platelet activation and aggregation. These processes may aggravate ongoing inflammation to produce vascular damage and lung injury and also drive damage in other susceptible organs, such as the kidneys. The hemorrhagic process in LNs is also supported by changes in the levels of hemopexin and haptoglobin. The latter is an acute phase glycoprotein that binds free hemoglobin with high avidity during hemolysis, protecting organs from iron-generated reactive oxygen species. In addition to sequestration of hemoglobin, haptoglobin is an alpha-2-glycoprotein with anti-inflammatory properties [55]. It was undetectable in the naive lymph but appeared in the infected one, consistent with its common upregulation in septic conditions, in response to inflammation, tissue damage, etc. Induction of hemopexin may reflect scavenging of heme released during cell damage. A significant increase in the relative abundance of actin and related proteins detected in a highly enriched group of regulation of actin cytoskeleton organization (Table 1) is consistent with the presence of hemorrhagic shock previously reported in rodent mesenteric lymph [13].

LNs are normally hypoxic (O_2_ pressure is about 70 mm Hg) and therefore likely represent one of several anatomical locations where anaerobic induction of pathogenic factors like classical anthrax lethal and edema toxins takes place [32], [56]–[58]. The toxin-mediated coagulopathy, vascular and epithelial disturbances involving signaling through mitogen-activated pathways are well established [32]. In addition, *B. anthracis* is able to produce an array of “accessory” pathogenic factors. Our previous data, including the analysis of blood proteome in anthrax-infected mice, indicate degradation of extracellular matrix and tissue damage due to proteolytic enzymes and hemolytic phospholipases [33]–[38], [58]–[61]. In this study we detected induction of a large number of proteinase inhibitors, perhaps in response to expression of bacterial or endogenous proteases involved in coagulation, fibrinolysis, complement activation, and inflammation.

We have recently demonstrated that the pore-forming toxin, anthrolysin O, secreted by bacteria in microaerobic growth conditions permeabilizes and kills the host cells in the synergistic interaction with the bacterial metabolite, succinate, in the process inducing host cell oxidative stress [59]. According to DAVID analysis, the proteome of infected LNs shows characteristic features associated with oxidative stress. Response to reactive oxygen species involves increased levels of ceruloplasmin; peroxiredoxins 1, 2, 5, 6; apolipoprotein E; superoxide dismutase; thioredoxin, carbonic anhydrase, and ATX1 (antioxidant protein 1) (although the glutathione system does not display substantial changes).

The down-regulated processes during infection (Tables S2 and S4) draw a picture of a widespread shutdown of cellular function evident in the decreased cellular metabolism of glucose, other monosaccharides, nitrogen compounds, and lipids along with the decreased aerobic respiration, suppressed malate dehydrogenases and other tricarboxylic acid cycle enzymes.

The above results of DAVID-based functional analysis are supported by the KEGG pathway mapping which assigned the up-regulated proteins to different processes in innate immune response including regulation of actin cytoskeleton and Fc receptor-mediated phagocytosis, complement and coagulation cascades, proteasome, ribosome, cell-cell adhesion and leukocyte migration (Table S5). Some of the pathways are illustrated by Fig. 3. The complement and coagulation cascades (Fig. 3A) indicate activation of both extrinsic and alternative mechanisms as a result of factors such as vascular injury and kallikrein-kinin system. Adherence junction cascades (Fig. 3B) demonstrate interconnections with cadherin function, cytokine responses, and cell cytoskeleton. The latter connects to the major innate immune cascades of leukocyte transmigration (Fig. 3C), cell-cell junctions, mitogen-activated kinases and chemotactic factors (Fig. 3D). The down-regulated proteins belong mainly to metabolic pathways (Tables S5, S6).

Lymph is usually referred to as the simple plasma filtrate and considered a necessary means of recycling excessive interstitial fluid [49], [62]. However, recent studies demonstrate that both tissue injury and shock correlate strongly with production of bioactive lymph. In addition to classic serum proteins, the physiologic response to trauma is associated with appearance of markers of hemolysis, extracellular matrix and general tissue damage, acute phase components, and cytokines [50], [52]–[53], [63]. As a result, lymph is considered as a candidate contributor to the development of systemic inflammation and multiple organ failure [15], [48], [50], [64].

Using modern proteomic approaches to characterize lymph, Interewicz et al [3] provided evidence for the existence of profound differences along with similarities between paired human lymph and plasma samples. Clement et al [48] revealed 144 human proteins common between the plasma and lymph, represented mainly by complement activation and blood coagulation components, transporters and protease inhibitors. In contrast, the enriched proteome of human plasma (37 proteins) consisted of soluble molecules of the coagulation system and cell-cell signaling factors. Yuan et al [49] reported that rat mesenteric lymph contained an array of proteins that differentiated it from the plasma. The most differentially expressed proteins in mesenteric lymph were gamma-fibrinogen, protease inhibitors, and proteins related to lipid transport/metabolism.

In this study we also wanted to determine the extent of commonality between serum and soluble content of LNs in naive and its dynamics in infected mice. Our data revealed peptides from 83 proteins common between serum and lymph in general agreement with previous data. Thus, the majority of the proteins found in lymph and serum appear to be unique to these body fluids. We found that the levels of common proteins in serum and lymph did not show a direct correlation, although in both cases the most abundant proteins included albumin, hemoglobin, complement component 3, transferrin, clade A serine proteinase inhibitor, apolipoproteins, alpha-2-HS-glycoprotein, hemopexin, vitamin D-binding protein, and adenylate kinase 1.

Levels of lymph proteins from the common list were sensitive to infection. Among the most up-regulated common proteins in lymph besides albumin were clade A serine proteinase inhibitors, transferrin, complement components, plasminogen, haptoglobin, apolipoprotein A-1, thrombin, hemopexin, hemoglobin, inter alpha- trypsin inhibitor and alpha 1. Thirty nine common proteins were absent in the naïve lymph and appeared only during infection. Among these with the highest level of expression were the proteins indicating cellular damage (haptoglobin, myoglobin), activation of proteolysis, coagulation and fibrinolysis (thrombin, fibrinogen, kininogen 1, proteinase inhibitors), oxidative stress (peroxiredoxin 5), and activation of complement. Our data cannot answer a question whether the appearance of new proteins is a result of increased exchange between serum and lymph (for example, due to tissue and vascular damage in LNs) or other mechanisms. On the other hand, 16 common proteins in Table 2 demonstrated a reduced abundance in lymph upon infection, indicating a considerable degree of independence among processes controlling the content of a particular protein in lymph *vs.* serum.

The circulating phosphoproteins have a potential to bear a wealth of information on the health status of the tissues and have recently been suggested as potential biomarkers [4], [45]. However, no data is available regarding their presence in normal lymph as well as during anthrax infection. We previously carried out the phosphoprotein analyses in serum using MS using titanium dioxide enrichment [44], [46]. The complexity of serum protein mixture and the high background level of the most abundant proteins make MS-based phosphoprotein identification and localization of phosphorylation sites a challenge. In this study we demonstrate that the RPMA assay using specific antibodies is suitable for reliable quantitative detection of signaling phosphoproteins in a small amount of material extracted from LNs. In our initial characterization of the LN proteome we chose to detect the proteins belonging to diverse groups representing critical cellular transduction pathways in anticipation that these pathways will reflect the specific features of infection within the LNs. Figure 3 illustrates some of the lymph phosphoproteins mapped to the cellular signaling pathways form which they likely originate.

Mitogen-activated protein kinase kinases are specific targets proteolytically inactivated by anthrax lethal toxin [56]. This inactivation results in the downregulation of phosphorylation of the Erk1/2, JNK and p38 signaling kinases and their downstream targets. The soluble phosphoproteome closely reflects this effect (Table 3). The levels of phosphorylated JNK (Thr183, Tyr185), ERK1/2 (Thr202, Tyr204), p38 (Thr180, Tyr182), and HSP27 (Ser82) are strongly reduced, although the total forms of these proteins are up-regulated. One of the important functional consequences of altered MAPK signaling is the induction of apoptosis/autophagy [42], [65], [66]. In agreement with this, all of the 18 apoptosis-related proteins tested in our experiments showed the abundance altered by infection. The phosphoproteins from LNs corresponding to the survival PTEN/PI3K/Akt pathway are also down-regulated as reported previously in cell culture and mice [43]. Among Src family kinases regulating growth and differentiation, the level of Src (Tyr416) activated form is reduced.

Profound changes are detectable at the level of transcriptional factors. The increased level of cAMP-responsive CREB was previously detected in the edema toxin-treated macrophages [67] and the epithelial cells infected with a non-toxinogenic *B. anthracis* [43]. CREB along with NF-kappaB presumably counterbalance the apoptotic effect of lethal toxin and delay cell death [32], [43].

The STAT family transcriptional factors participate in the JAK-STAT pleiotropic cascades allowing the cells to transduce signals for a large number of hormones, growth factors, and cytokines. One of the STATs’ most important functions is to regulate T cell differentiation [68]. Our data show activation of STAT1 (Tyr701) and STAT3 (Tyr705, Ser727) in contrast to STAT5 (Tyr694) and STAT6 (Tyr641) which might reflect up-regulation of Th17, Th1 and Treg and down-regulation of Th2 cell subsets, in line with the previous findings showing that anthrax infection manipulates with the cytokine and T cell responses [32].

In summary, we report the first description of the soluble proteome from the LNs of mice before and after cutaneous challenge with *B. anthracis* Sterne spores. We demonstrate the lymphatic system serves as a sensitive sentinel of infection responding to it with a large number of up- and down-regulated proteins many of which are distinct from those detected in serum. These proteins belong to an array of processes reflecting response to wounding, inflammation and perturbations of hemostasis, innate immune response, coagulation and fibrinolysis, regulation of body fluid levels and vascular disturbance among others. All of these processes bear close relevance to the pathogenic features described in anthrax infection previously. Also, we carried out initial characterization of circulating lymph phosphoproteome which brought additional information regarding signaling pathways operating in lymphatics. However, our model employs an attenuated Sterne strain (pXO2^−^) devoid of the poly-γ-D-glutamic acid capsule and therefore does not reflect the effects of this virulence factor on the proteomic changes in lymphatics. In our upcoming research we plan to address this topic using our experimental approach while focusing on deeper exploration of the specific host responses to different pathogenic factors of *B. anthracis*.

## Supporting Information

Table S1 **LN Proteins from Naïve Mice Ranked on the Number of Spectral Hits.**
(DOCX)

Table S2 **Proteins identified in the soluble LN content of B. anthracis-challenged mice in comparison with uninfected mice.**
(DOCX)

Table S3 **Top-Scoring Clusters of the GO Terms Corresponding to the Proteins Upregulated in Infection.**
(DOCX)

Table S4 **Top-Scoring Clusters of the GO Terms Corresponding to the Proteins Down-regulated in Infection.**
(DOCX)

Table S5 **KEGG processes identified for lymph proteins up-regulated by infection.**
(DOCX)

Table S6 **KEGG processes identified for lymph proteins down-regulated by infection.**
(DOCX)

Table S7 **Proteins from naïve mice serum.**
(DOCX)
