# Supplementary material for: Whole Proteome Analysis of Mouse Lymph Nodes in Cutaneous Anthrax
Source: PLoS One. 2014 Oct 20;9(10):e110873. doi: 10.1371/journal.pone.0110873 (PMC4203832; doi:10.1371/journal.pone.0110873)
Supplement: Table S1 — LN Proteins from Naïve Mice Ranked on the Number of Spectral Hits. (DOCX) [file pone.0110873.s001.docx]

**Table S1. LN Proteins from Naïve Mice Ranked on the Number of Spectral Hits**

|  | Reference | GI number | AV* | SD** | CI*** | CI/AV |
| --- | --- | --- | --- | --- | --- | --- |
| 1 | serum albumin precursor | 163310765 | 268.5 | 41.83 | 53.87 | 0.20 |
| 2 | creatine kinase M-type | 6671762 | 211.75 | 31.07 | 40.02 | 0.19 |
| 3 | parvalbumin alpha | 31980767 | 94 | 14.83 | 19.10 | 0.20 |
| 4 | fatty acid synthase | 93102409 | 86.5 | 26.10 | 33.61 | 0.39 |
| 5 | serotransferrin precursor | 20330802 | 53 | 10.55 | 13.59 | 0.26 |
| 6 | fatty acid-binding protein, adipocyte | 14149635 | 52.5 | 12.48 | 16.07 | 0.31 |
| 7 | glyceraldehyde-3-phosphate dehydrogenase | 6679937 | 48.25 | 4.35 | 5.60 | 0.12 |
| 8 | PREDICTED: pyruvate kinase, muscle isoform X4 | 568960825 | 43 | 4.76 | 6.13 | 0.14 |
| 9 | fructose-bisphosphate aldolase A isoform 1 precursor | 293597567 | 42 | 9.70 | 12.49 | 0.30 |
| 10 | PREDICTED: alpha-enolase isoform X2 | 568930540 | 40 | 9.70 | 12.49 | 0.31 |
| 11 | glycogen phosphorylase, muscle form | 6755256 | 39.75 | 9.81 | 12.64 | 0.32 |
| 12 | carbonic anhydrase 3 | 31982861 | 33 | 3.37 | 4.34 | 0.13 |
| 13 | PREDICTED: beta-enolase isoform X1 | 568971525 | 31.75 | 7.54 | 9.72 | 0.31 |
| 14 | transketolase | 6678359 | 29 | 3.56 | 4.58 | 0.16 |
| 15 | L-lactate dehydrogenase A chain isoform 2 | 257743039 | 24.5 | 3.11 | 4.00 | 0.16 |
| 16 | aldolase 1 A retrogene 1 | 312922382 | 23.25 | 7.41 | 9.54 | 0.41 |
| 17 | triosephosphate isomerase | 226958349 | 22.25 | 3.40 | 4.38 | 0.20 |
| 18 | alpha-2-macroglobulin precursor | 110347469 | 21.25 | 2.06 | 2.66 | 0.12 |
| 19 | hemopexin precursor | 160358829 | 21 | 8.04 | 10.36 | 0.49 |
| 20 | serine protease inhibitor A3K precursor | 148747546 | 20.5 | 5.26 | 6.77 | 0.33 |
| 21 | ATP-citrate synthase isoform 2 | 29293809 | 20 | 7.75 | 9.98 | 0.50 |
| 22 | phosphoglycerate kinase 1 | 70778976 | 19.75 | 1.71 | 2.20 | 0.11 |
| 23 | adenylate kinase isoenzyme 1 isoform 2 | 311771688 | 18.75 | 2.99 | 3.85 | 0.21 |
| 24 | glycerol-3-phosphate dehydrogenase | 6753966 | 18 | 2.16 | 2.78 | 0.15 |
| 25 | alpha-1-antitrypsin 1-2 precursor | 76881807 | 18 | 4.97 | 6.40 | 0.36 |
| 26 | phosphoglucomutase-2 | 227330633 | 17.5 | 2.38 | 3.07 | 0.18 |
| 27 | annexin A2 | 6996913 | 17.25 | 0.96 | 1.23 | 0.07 |
| 28 | hemoglobin subunit alpha | 145301578 | 17.25 | 2.63 | 3.39 | 0.20 |
| 29 | hemoglobin, beta adult t chain | 31982300 | 17 | 3.83 | 4.93 | 0.29 |
| 30 | complement C3 precursor | 126518317 | 16 | 2.31 | 2.97 | 0.19 |
| 31 | phosphoglycerate mutase 2 | 9256624 | 16 | 6.06 | 7.80 | 0.49 |
| 32 | annexin A6 isoform b | 158966670 | 15.25 | 1.71 | 2.20 | 0.14 |
| 33 | four and a half LIM domains protein 1 isoform 2 | 116517336 | 15.25 | 5.12 | 6.60 | 0.43 |
| 34 | tubulin beta-4B chain | 22165384 | 14.75 | 2.22 | 2.86 | 0.19 |
| 35 | heat shock cognate 71 kDa protein | 31981690 | 14.75 | 3.77 | 4.86 | 0.33 |
| 36 | glucose-6-phosphate isomerase | 254553458 | 13.25 | 2.99 | 3.85 | 0.29 |
| 37 | annexin A5 | 6753060 | 11.75 | 1.71 | 2.20 | 0.19 |
| 38 | alpha-2-HS-glycoprotein isoform 1 precursor | 7304875 | 11.25 | 0.96 | 1.23 | 0.11 |
| 39 | actin, alpha cardiac muscle 1 | 14192922 | 11 | 2.16 | 2.78 | 0.25 |
| 40 | glycogen phosphorylase, brain form | 24418919 | 10.5 | 3.70 | 4.76 | 0.45 |
| 41 | elongation factor 2 | 33859482 | 10.25 | 2.36 | 3.04 | 0.30 |
| 42 | myoglobin | 21359820 | 10.25 | 4.03 | 5.19 | 0.51 |
| 43 | PREDICTED: 6-phosphofructokinase, muscle type isoform X1 | 568991689 | 9.75 | 2.36 | 3.04 | 0.31 |
| 44 | malate dehydrogenase, cytoplasmic | 254540027 | 9.25 | 1.26 | 1.62 | 0.18 |
| 45 | actin, cytoplasmic 1 | 6671509 | 9.25 | 2.22 | 2.86 | 0.31 |
| 46 | transitional endoplasmic reticulum ATPase | 225543319 | 9 | 1.83 | 2.35 | 0.26 |
| 47 | tubulin alpha-4A chain | 6678467 | 9 | 2.58 | 3.33 | 0.37 |
| 48 | vitamin D-binding protein precursor | 51172612 | 9 | 2.58 | 3.33 | 0.37 |
| 49 | malate dehydrogenase, mitochondrial precursor | 31982186 | 9 | 2.94 | 3.79 | 0.42 |
| 50 | serine protease inhibitor A3C precursor | 6680586 | 8.75 | 1.26 | 1.62 | 0.19 |
| 51 | plastin-2 | 31543113 | 8.5 | 2.65 | 3.41 | 0.40 |
| 52 | murinoglobulin-1 precursor | 31982171 | 8.5 | 7.14 | 9.20 | 1.08 |
| 53 | cofilin-1 | 6680924 | 8.25 | 0.96 | 1.23 | 0.15 |
| 54 | superoxide dismutase | 45597447 | 7.75 | 3.10 | 3.99 | 0.51 |
| 55 | hemoglobin subunit beta-2 | 17647499 | 7.5 | 0.58 | 0.74 | 0.10 |
| 56 | aspartate aminotransferase, cytoplasmic | 160298209 | 7.25 | 1.26 | 1.62 | 0.22 |
| 57 | ubiquitin-like modifier-activating enzyme 1 isoform 1 | 444189294 | 7.25 | 1.71 | 2.20 | 0.30 |
| 58 | apolipoprotein A-I preproprotein | 160333304 | 7.25 | 2.22 | 2.86 | 0.39 |
| 59 | aldose reductase | 160707894 | 7 | 2.71 | 3.49 | 0.50 |
| 60 | acyl-CoA-binding protein isoform 2 | 6681137 | 6.75 | 0.50 | 0.64 | 0.10 |
| 61 | 14-3-3 protein zeta/delta isoform 1 | 6756041 | 6.75 | 0.50 | 0.64 | 0.10 |
| 62 | adenylosuccinate synthetase isozyme 1 | 6671519 | 6.75 | 1.71 | 2.20 | 0.33 |
| 63 | protein DJ-1 | 55741460 | 6.75 | 1.71 | 2.20 | 0.33 |
| 64 | phosphatidylethanolamine-binding protein 1 | 84794552 | 6.5 | 0.58 | 0.74 | 0.11 |
| 65 | PREDICTED: acetyl-CoA carboxylase 1 isoform X3 | 568971082 | 6.5 | 4.43 | 5.71 | 0.88 |
| 66 | PREDICTED: peptidyl-prolyl cis-trans isomerase A-like | 568952044 | 6.25 | 0.50 | 0.64 | 0.10 |
| 67 | 14-3-3 protein gamma | 31543976 | 6.25 | 0.96 | 1.23 | 0.20 |
| 68 | PREDICTED: heat shock protein HSP 90-alpha isoform X1 | 568978837 | 6.25 | 1.26 | 1.62 | 0.26 |
| 69 | myosin heavy chain IIa | 205830428 | 6 | 4.16 | 5.36 | 0.89 |
| 70 | aconitate hydratase, mitochondrial precursor | 18079339 | 5.75 | 2.99 | 3.85 | 0.67 |
| 71 | myosin-9 | 114326446 | 5.75 | 6.02 | 7.75 | 1.35 |
| 72 | peroxiredoxin-5, mitochondrial precursor | 6755114 | 5.5 | 0.58 | 0.74 | 0.14 |
| 73 | myosin-4 | 67189167 | 5.5 | 2.08 | 2.68 | 0.49 |
| 74 | glutathione S-transferase Mu 5 | 6754086 | 5.5 | 9.04 | 11.64 | 2.12 |
| 75 | hemoglobin subunit beta-1 | 498752597 | 5.25 | 0.96 | 1.23 | 0.23 |
| 76 | heat shock protein HSP 90-beta | 40556608 | 5.25 | 2.22 | 2.86 | 0.54 |
| 77 | myosin light chain 1/3, skeletal muscle isoform isoform 1f | 29789016 | 5.25 | 5.19 | 6.68 | 1.27 |
| 78 | peroxiredoxin-1 | 6754976 | 5.25 | 5.38 | 6.93 | 1.32 |
| 79 | 14-3-3 protein epsilon | 226874906 | 5 | 0.82 | 1.05 | 0.21 |
| 80 | NADP-dependent malic enzyme isoform 1 | 162139827 | 4.75 | 0.50 | 0.64 | 0.14 |
| 81 | carboxylesterase 1C precursor | 247269929 | 4.75 | 0.50 | 0.64 | 0.14 |
| 82 | elongation factor 1-alpha 2 | 6681273 | 4.75 | 0.96 | 1.23 | 0.26 |
| 83 | PREDICTED: gelsolin isoform X1 | 568913585 | 4.75 | 0.96 | 1.23 | 0.26 |
| 84 | lactoylglutathione lyase | 165932331 | 4.75 | 1.50 | 1.93 | 0.41 |
| 85 | fatty acid-binding protein, heart | 6753810 | 4.75 | 1.71 | 2.20 | 0.46 |
| 86 | rab GDP dissociation inhibitor beta | 116089273 | 4.75 | 1.71 | 2.20 | 0.46 |
| 87 | isocitrate dehydrogenase | 162417975 | 4.75 | 1.71 | 2.20 | 0.46 |
| 88 | profilin-1 | 6755040 | 4.5 | 0.58 | 0.74 | 0.17 |
| 89 | PREDICTED: pyruvate kinase, muscle isoform X2 | 568960821 | 4.5 | 1.00 | 1.29 | 0.29 |
| 90 | carboxylesterase 1B precursor | 124487013 | 4.5 | 1.29 | 1.66 | 0.37 |
| 91 | myosin-3 | 153792649 | 4.5 | 2.38 | 3.07 | 0.68 |
| 92 | glutamine synthetase | 31982332 | 4.25 | 0.96 | 1.23 | 0.29 |
| 93 | annexin A1 | 124517663 | 4.25 | 0.96 | 1.23 | 0.29 |
| 94 | selenium-binding protein 2 | 9507079 | 4.25 | 1.26 | 1.62 | 0.38 |
| 95 | transgelin-2 | 30519911 | 4.25 | 2.63 | 3.39 | 0.80 |
| 96 | moesin | 70778915 | 4.25 | 2.87 | 3.70 | 0.87 |
| 97 | PREDICTED: alpha-enolase isoform X1 | 568930538 | 4.25 | 5.06 | 6.51 | 1.53 |
| 98 | apolipoprotein A-IV precursor | 110347473 | 4 | 0.82 | 1.05 | 0.26 |
| 99 | PREDICTED: myosin light polypeptide 6-like, partial | 568958440 | 4 | 1.15 | 1.49 | 0.37 |
| 100 | alpha-1-antitrypsin 1-4 precursor | 6678085 | 4 | 1.41 | 1.82 | 0.46 |
| 101 | radixin isoform a | 157277948 | 3.75 | 0.50 | 0.64 | 0.17 |
| 102 | ribonuclease inhibitor isoform b | 285402659 | 3.75 | 0.50 | 0.64 | 0.17 |
| 103 | guanine nucleotide-binding protein subunit beta-2-like 1 | 6680047 | 3.75 | 0.96 | 1.23 | 0.33 |
| 104 | myosin regulatory light chain 2, skeletal muscle isoform | 7949078 | 3.75 | 0.96 | 1.23 | 0.33 |
| 105 | keratin, type II cytoskeletal 1 | 126116585 | 3.75 | 2.06 | 2.66 | 0.71 |
| 106 | coronin-1A | 6753492 | 3.75 | 2.22 | 2.86 | 0.76 |
| 107 | alpha-2-macroglobulin-P precursor | 148277039 | 3.75 | 6.18 | 7.97 | 2.12 |
| 108 | fibrinogen, alpha polypeptide isoform 1 precursor | 167555029 | 3.5 | 0.58 | 0.74 | 0.21 |
| 109 | PREDICTED: ceruloplasmin isoform X1 | 568920738 | 3.5 | 0.58 | 0.74 | 0.21 |
| 110 | peroxiredoxin-2 | 148747558 | 3.5 | 1.00 | 1.29 | 0.37 |
| 111 | rho GDP-dissociation inhibitor 1 | 31982030 | 3.5 | 1.29 | 1.66 | 0.48 |
| 112 | 6-phosphogluconate dehydrogenase, decarboxylating | 124486895 | 3.5 | 1.73 | 2.23 | 0.64 |
| 113 | PREDICTED: 14-3-3 protein theta-like | 568928017 | 3.25 | 0.96 | 1.23 | 0.38 |
| 114 | PREDICTED: heterogeneous nuclear ribonucleoprotein K isoform X7 | 568983058 | 3.25 | 0.96 | 1.23 | 0.38 |
| 115 | gamma-synuclein | 6755592 | 3.25 | 1.26 | 1.62 | 0.50 |
| 116 | actin-related protein 3 | 23956222 | 3.25 | 2.06 | 2.66 | 0.82 |
| 117 | adenylyl cyclase-associated protein 1 | 157951604 | 3.25 | 2.06 | 2.66 | 0.82 |
| 118 | PREDICTED: AMP deaminase 1 isoform X1 | 568922624 | 3.25 | 2.36 | 3.04 | 0.94 |
| 119 | tubulin beta-5 chain | 7106439 | 3 | 0.82 | 1.05 | 0.35 |
| 120 | lumican precursor | 160333372 | 3 | 0.82 | 1.05 | 0.35 |
| 121 | cofilin-2 | 6671746 | 3 | 1.15 | 1.49 | 0.50 |
| 122 | 14-3-3 protein beta/alpha | 31543974 | 3 | 1.41 | 1.82 | 0.61 |
| 123 | fibrinogen beta chain precursor | 33859809 | 3 | 1.83 | 2.35 | 0.78 |
| 124 | histone H1.3 | 254588110 | 3 | 2.16 | 2.78 | 0.93 |
| 125 | apolipoprotein A-II precursor | 157951676 | 3 | 4.69 | 6.04 | 2.01 |
| 126 | alpha-1B-glycoprotein precursor | 124486702 | 3 | 0.00 |  |  |
| 127 | peroxiredoxin-6 | 6671549 | 2.75 | 0.50 | 0.64 | 0.23 |
| 128 | calmodulin | 6680834 | 2.75 | 0.50 | 0.64 | 0.23 |
| 129 | antithrombin-III precursor | 18252782 | 2.75 | 0.50 | 0.64 | 0.23 |
| 130 | fibrinogen gamma chain precursor | 19527078 | 2.75 | 0.50 | 0.64 | 0.23 |
| 131 | adenosylhomocysteinase | 262263372 | 2.75 | 0.50 | 0.64 | 0.23 |
| 132 | PREDICTED: glutathione S-transferase Mu 7 isoform X1 | 568923826 | 2.75 | 0.50 | 0.64 | 0.23 |
| 133 | protease, serine, 1 precursor | 16716569 | 2.75 | 0.96 | 1.23 | 0.45 |
| 134 | keratin, type II cytoskeletal 75 | 29789317 | 2.75 | 0.96 | 1.23 | 0.45 |
| 135 | tubulin alpha-1B chain | 34740335 | 2.75 | 1.26 | 1.62 | 0.59 |
| 136 | plasminogen precursor | 257471003 | 2.75 | 1.26 | 1.62 | 0.59 |
| 137 | glycogen phosphorylase, liver form | 268836255 | 2.75 | 1.50 | 1.93 | 0.70 |
| 138 | PREDICTED: keratin, type I cytoskeletal 10 isoform X1 | 568971870 | 2.75 | 1.50 | 1.93 | 0.70 |
| 139 | WD repeat-containing protein 1 | 6755995 | 2.75 | 1.71 | 2.20 | 0.80 |
| 140 | PREDICTED: dihydropyrimidinase-related protein 2 isoform X1 | 568986628 | 2.75 | 1.71 | 2.20 | 0.80 |
| 141 | troponin C, skeletal muscle | 6678371 | 2.5 | 0.58 | 0.74 | 0.30 |
| 142 | clathrin heavy chain 1 | 51491845 | 2.5 | 0.58 | 0.74 | 0.30 |
| 143 | 6-phosphofructokinase, liver type | 31560653 | 2.5 | 1.00 | 1.29 | 0.52 |
| 144 | PREDICTED: protein S100-A11 isoform X1 | 568922148 | 2.5 | 1.00 | 1.29 | 0.52 |
| 145 | PREDICTED: acetyl-Coenzyme A carboxylase beta isoform X1 | 568936755 | 2.5 | 1.00 | 1.29 | 0.52 |
| 146 | annexin A4 | 161016799 | 2.5 | 1.29 | 1.66 | 0.67 |
| 147 | electron transfer flavoprotein subunit alpha, mitochondrial | 227500281 | 2.5 | 1.29 | 1.66 | 0.67 |
| 148 | 6-phosphogluconolactonase | 13384778 | 2.5 | 1.73 | 2.23 | 0.89 |
| 149 | acylphosphatase-2 | 27229219 | 2.5 | 1.73 | 2.23 | 0.89 |
| 150 | glycogen debranching enzyme | 124486747 | 2.5 | 1.73 | 2.23 | 0.89 |
| 151 | probable C-_U-editing enzyme APOBEC-2 | 6753098 | 2.5 | 1.91 | 2.47 | 0.99 |
| 152 | proteasome activator complex subunit 1 | 6755212 | 2.25 | 0.50 | 0.64 | 0.29 |
| 153 | heterogeneous nuclear ribonucleoprotein D0 isoform d | 116256516 | 2.25 | 0.50 | 0.64 | 0.29 |
| 154 | alpha-1-antitrypsin 1-1 isoform 2 | 357588427 | 2.25 | 0.50 | 0.64 | 0.29 |
| 155 | PREDICTED: vinculin isoform X1 | 568987488 | 2.25 | 0.50 | 0.64 | 0.29 |
| 156 | polymerase I and transcript release factor | 6679567 | 2.25 | 0.96 | 1.23 | 0.55 |
| 157 | kininogen-1 isoform 2 precursor | 12963497 | 2.25 | 1.26 | 1.62 | 0.72 |
| 158 | EH domain-containing protein 2 | 55742711 | 2.25 | 1.26 | 1.62 | 0.72 |
| 159 | nucleolin | 84875537 | 2.25 | 1.89 | 2.44 | 1.08 |
| 160 | carboxylesterase 1-like precursor | 283135142 | 2.25 | 3.86 | 4.97 | 2.21 |
| 161 | galectin-1 | 6678682 | 2 | 0.82 | 1.05 | 0.53 |
| 162 | proteasome subunit alpha type-7 | 7106389 | 2 | 0.82 | 1.05 | 0.53 |
| 163 | UTP--glucose-1-phosphate uridylyltransferase | 21314832 | 2 | 0.82 | 1.05 | 0.53 |
| 164 | histone H2A type 2-C | 30089710 | 2 | 0.82 | 1.05 | 0.53 |
| 165 | inter alpha-trypsin inhibitor, heavy chain 4 isoform 1 precursor | 226531047 | 2 | 0.82 | 1.05 | 0.53 |
| 166 | PREDICTED: keratin Kb40 isoform X1 | 568992743 | 2 | 0.82 | 1.05 | 0.53 |
| 167 | phosphoglycerate kinase 2 | 226246531 | 2 | 1.15 | 1.49 | 0.74 |
| 168 | thymosin beta-4 | 10946578 | 2 | 1.83 | 2.35 | 1.18 |
| 169 | elongation factor 1-gamma | 110625979 | 2 | 1.83 | 2.35 | 1.18 |
| 170 | cysteine and glycine-rich protein 1 | 6681069 | 2 | 2.16 | 2.78 | 1.39 |
| 171 | serine/threonine-protein phosphatase 2A catalytic subunit beta isoform | 8394024 | 2 | 0.00 |  |  |
| 172 | serine protease inhibitor A3F | 269973844 | 2 | 0.00 |  |  |
| 173 | tubulin alpha-3 chain | 6678465 | 1.75 | 0.50 | 0.64 | 0.37 |
| 174 | L-lactate dehydrogenase B chain | 6678674 | 1.75 | 0.50 | 0.64 | 0.37 |
| 175 | cathepsin B preproprotein | 6681079 | 1.75 | 0.50 | 0.64 | 0.37 |
| 176 | keratin, type II cytoskeletal 79 | 22164776 | 1.75 | 0.50 | 0.64 | 0.37 |
| 177 | PREDICTED: alpha-actinin-4 isoform X3 | 568946340 | 1.75 | 0.50 | 0.64 | 0.37 |
| 178 | PREDICTED: myc box-dependent-interacting protein 1 isoform X1 | 569003953 | 1.75 | 0.50 | 0.64 | 0.37 |
| 179 | translationally-controlled tumor protein | 6678437 | 1.75 | 0.96 | 1.23 | 0.70 |
| 180 | poly(rC)-binding protein 1 | 6754994 | 1.75 | 0.96 | 1.23 | 0.70 |
| 181 | eukaryotic translation initiation factor 5A-2 | 29243942 | 1.75 | 0.96 | 1.23 | 0.70 |
| 182 | transaldolase | 33859640 | 1.75 | 0.96 | 1.23 | 0.70 |
| 183 | tropomyosin alpha-3 chain isoform 1 | 40254525 | 1.75 | 0.96 | 1.23 | 0.70 |
| 184 | fascin | 113680348 | 1.75 | 0.96 | 1.23 | 0.70 |
| 185 | fatty acid-binding protein, epidermal isoform 1 | 6754450 | 1.75 | 1.26 | 1.62 | 0.93 |
| 186 | glucose-6-phosphate 1-dehydrogenase X | 6996917 | 1.75 | 1.26 | 1.62 | 0.93 |
| 187 | coactosin-like protein | 19482160 | 1.75 | 1.26 | 1.62 | 0.93 |
| 188 | annexin A3 | 160707925 | 1.75 | 1.26 | 1.62 | 0.93 |
| 189 | hydroxyacyl-coenzyme A dehydrogenase, mitochondrial precursor | 111038118 | 1.75 | 1.50 | 1.93 | 1.10 |
| 190 | cytosolic 10-formyltetrahydrofolate dehydrogenase | 27532959 | 1.75 | 1.71 | 2.20 | 1.26 |
| 191 | PREDICTED: high mobility group protein B1-like | 407262920 | 1.75 | 1.71 | 2.20 | 1.26 |
| 192 | PREDICTED: pyruvate kinase PKLR isoform X2 | 568922076 | 1.75 | 1.71 | 2.20 | 1.26 |
| 193 | alcohol dehydrogenase 1 | 6724311 | 1.5 | 0.58 | 0.74 | 0.50 |
| 194 | keratin, type II cytoskeletal 71 | 9910294 | 1.5 | 0.58 | 0.74 | 0.50 |
| 195 | tubulin beta-3 chain | 12963615 | 1.5 | 0.58 | 0.74 | 0.50 |
| 196 | protein SET isoform 1 | 13591862 | 1.5 | 0.58 | 0.74 | 0.50 |
| 197 | obg-like ATPase 1 isoform a | 21313144 | 1.5 | 0.58 | 0.74 | 0.50 |
| 198 | glial fibrillary acidic protein isoform 2 | 84000448 | 1.5 | 0.58 | 0.74 | 0.50 |
| 199 | phosphoglycerate mutase 1 | 114326546 | 1.5 | 0.58 | 0.74 | 0.50 |
| 200 | filamin-C | 124487139 | 1.5 | 0.58 | 0.74 | 0.50 |
| 201 | PREDICTED: glutathione S-transferase P 2-like | 149247503 | 1.5 | 0.58 | 0.74 | 0.50 |
| 202 | fumarylacetoacetase | 240120112 | 1.5 | 0.58 | 0.74 | 0.50 |
| 203 | 78 kDa glucose-regulated protein precursor | 254540166 | 1.5 | 0.58 | 0.74 | 0.50 |
| 204 | PREDICTED: gamma-enolase isoform X3 | 568940593 | 1.5 | 0.58 | 0.74 | 0.50 |
| 205 | PREDICTED: myosin-14 isoform X2 | 568948526 | 1.5 | 0.58 | 0.74 | 0.50 |
| 206 | PREDICTED: ester hydrolase C11orf54 homolog isoform X2 | 568960050 | 1.5 | 0.58 | 0.74 | 0.50 |
| 207 | PREDICTED: myosin-10 isoform X1 | 568976394 | 1.5 | 0.58 | 0.74 | 0.50 |
| 208 | cell division control protein 42 homolog isoform 1 precursor | 6753364 | 1.5 | 1.00 | 1.29 | 0.86 |
| 209 | PREDICTED: meiotic recombination protein SPO11 isoform X1 | 568917233 | 1.5 | 1.00 | 1.29 | 0.86 |
| 210 | peptidyl-prolyl cis-trans isomerase A | 6679439 | 1.5 | 1.00 | 1.29 | 0.86 |
| 211 | PREDICTED: sarcoplasmic/endoplasmic reticulum calcium ATPase 1 isoform X1 | 568949470 | 1.5 | 1.00 | 1.29 | 0.86 |
| 212 | glutathione S-transferase Mu 1 | 6754084 | 1.5 | 1.29 | 1.66 | 1.11 |
| 213 | chloride intracellular channel protein 1 | 15617203 | 1.5 | 1.29 | 1.66 | 1.11 |
| 214 | beta-actin-like protein 2 | 30425250 | 1.5 | 1.29 | 1.66 | 1.11 |
| 215 | cytosolic non-specific dipeptidase | 31981273 | 1.5 | 1.29 | 1.66 | 1.11 |
| 216 | heterogeneous nuclear ribonucleoproteins A2/B1 isoform 2 | 32880197 | 1.5 | 1.29 | 1.66 | 1.11 |
| 217 | leukocyte elastase inhibitor A | 114158675 | 1.5 | 1.29 | 1.66 | 1.11 |
| 218 | PREDICTED: LIM domain-binding protein 3 isoform X2 | 568987690 | 1.5 | 1.29 | 1.66 | 1.11 |
| 219 | keratin, type II cytoskeletal 2 epidermal | 124487419 | 1.5 | 1.73 | 2.23 | 1.49 |
| 220 | cytochrome c, somatic | 6681095 | 1.25 | 0.50 | 0.64 | 0.52 |
| 221 | enoyl-CoA delta isomerase 1, mitochondrial precursor | 31981810 | 1.25 | 0.50 | 0.64 | 0.52 |
| 222 | histidine triad nucleotide-binding protein 1 | 33468857 | 1.25 | 0.50 | 0.64 | 0.52 |
| 223 | uncharacterized protein LOC75471 | 124487358 | 1.25 | 0.50 | 0.64 | 0.52 |
| 224 | polyubiquitin-C | 157671923 | 1.25 | 0.50 | 0.64 | 0.52 |
| 225 | annexin A11 | 160707921 | 1.25 | 0.50 | 0.64 | 0.52 |
| 226 | nucleosome assembly protein 1-like 1 isoform 1 precursor | 226443026 | 1.25 | 0.50 | 0.64 | 0.52 |
| 227 | PREDICTED: acidic leucine-rich nuclear phosphoprotein 32 family member B isoform X1 | 568927278 | 1.25 | 0.50 | 0.64 | 0.52 |
| 228 | PREDICTED: rho GDP-dissociation inhibitor 2 isoform X1 | 568940382 | 1.25 | 0.50 | 0.64 | 0.52 |
| 229 | indolethylamine N-methyltransferase | 6678281 | 1.25 | 0.96 | 1.23 | 0.99 |
| 230 | thyroid hormone-inducible hepatic protein | 6678345 | 1.25 | 0.96 | 1.23 | 0.99 |
| 231 | medium-chain specific acyl-CoA dehydrogenase, mitochondrial precursor | 6680618 | 1.25 | 0.96 | 1.23 | 0.99 |
| 232 | copper transport protein ATOX1 | 6753136 | 1.25 | 0.96 | 1.23 | 0.99 |
| 233 | heterogeneous nuclear ribonucleoprotein A/B isoform 2 | 6754222 | 1.25 | 0.96 | 1.23 | 0.99 |
| 234 | sarcoplasmic/endoplasmic reticulum calcium ATPase 2 isoform b | 6806903 | 1.25 | 0.96 | 1.23 | 0.99 |
| 235 | 60S ribosomal protein L7a | 7305443 | 1.25 | 0.96 | 1.23 | 0.99 |
| 236 | glutaredoxin-1 | 31981458 | 1.25 | 0.96 | 1.23 | 0.99 |
| 237 | PREDICTED: 60S ribosomal protein L30-like | 82890078 | 1.25 | 0.96 | 1.23 | 0.99 |
| 238 | elongation factor 1-alpha 1 | 126032329 | 1.25 | 0.96 | 1.23 | 0.99 |
| 239 | low molecular weight phosphotyrosine protein phosphatase isoform 1 | 159032062 | 1.25 | 0.96 | 1.23 | 0.99 |
| 240 | rho-related GTP-binding protein RhoC precursor | 160415213 | 1.25 | 0.96 | 1.23 | 0.99 |
| 241 | UMP-CMP kinase | 165377065 | 1.25 | 0.96 | 1.23 | 0.99 |
| 242 | myosin-binding protein C, fast-type | 268370244 | 1.25 | 0.96 | 1.23 | 0.99 |
| 243 | PREDICTED: protein NDRG2 isoform X1 | 568987854 | 1.25 | 0.96 | 1.23 | 0.99 |
| 244 | high mobility group protein B2 | 6680229 | 1.25 | 1.26 | 1.62 | 1.30 |
| 245 | carbonyl reductase | 113680352 | 1.25 | 1.26 | 1.62 | 1.30 |
| 246 | PREDICTED: nucleoside diphosphate kinase B-like | 568939339 | 1.25 | 1.50 | 1.93 | 1.55 |
| 247 | 40S ribosomal protein SA | 224994260 | 1.25 | 1.89 | 2.44 | 1.95 |
| 248 | glutathione S-transferase Mu 2 | 6680121 | 1 | 0.82 | 1.05 | 1.05 |
| 249 | SH3 domain-binding glutamic acid-rich-like protein | 9910548 | 1 | 0.82 | 1.05 | 1.05 |
| 250 | creatine kinase S-type, mitochondrial precursor | 38259206 | 1 | 0.82 | 1.05 | 1.05 |
| 251 | D-3-phosphoglycerate dehydrogenase | 52353955 | 1 | 0.82 | 1.05 | 1.05 |
| 252 | AHNAK nucleoprotein isoform 1 | 61743961 | 1 | 0.82 | 1.05 | 1.05 |
| 253 | myosin-1 | 82524274 | 1 | 0.82 | 1.05 | 1.05 |
| 254 | 60S ribosomal protein L18 | 83699424 | 1 | 0.82 | 1.05 | 1.05 |
| 255 | thioredoxin reductase 1, cytoplasmic isoform 1 | 110224447 | 1 | 0.82 | 1.05 | 1.05 |
| 256 | heat shock 70 kDa protein 4 | 112293266 | 1 | 0.82 | 1.05 | 1.05 |
| 257 | fructose-1,6-bisphosphatase isozyme 2 | 122937183 | 1 | 0.82 | 1.05 | 1.05 |
| 258 | T-complex protein 1 subunit theta | 126723461 | 1 | 0.82 | 1.05 | 1.05 |
| 259 | 40S ribosomal protein S16 | 158966704 | 1 | 0.82 | 1.05 | 1.05 |
| 260 | bifunctional purine biosynthesis protein PURH | 227908823 | 1 | 0.82 | 1.05 | 1.05 |
| 261 | serpin B6 isoform a | 255759941 | 1 | 0.82 | 1.05 | 1.05 |
| 262 | PREDICTED: biliverdin reductase A isoform X2 | 568915053 | 1 | 0.82 | 1.05 | 1.05 |
| 263 | PREDICTED: 40S ribosomal protein S8-like | 568920033 | 1 | 0.82 | 1.05 | 1.05 |
| 264 | PREDICTED: talin-1 isoform X2 | 568926384 | 1 | 0.82 | 1.05 | 1.05 |
| 265 | PREDICTED: small ubiquitin-related modifier 2-like | 568933472 | 1 | 0.82 | 1.05 | 1.05 |
| 266 | PREDICTED: prostaglandin E synthase 3-like | 568943642 | 1 | 0.82 | 1.05 | 1.05 |
| 267 | glutathione S-transferase Mu 3 | 33468899 | 1 | 1.15 | 1.49 | 1.49 |
| 268 | 60S acidic ribosomal protein P2 | 83745120 | 1 | 1.15 | 1.49 | 1.49 |
| 269 | protein disulfide-isomerase A3 precursor | 112293264 | 1 | 1.15 | 1.49 | 1.49 |
| 270 | perilipin-1 | 164698408 | 1 | 1.15 | 1.49 | 1.49 |
| 271 | glycerol-3-phosphate dehydrogenase 1-like protein | 257467604 | 1 | 1.15 | 1.49 | 1.49 |
| 272 | 60S ribosomal protein L22 isoform b | 459683845 | 1 | 1.15 | 1.49 | 1.49 |
| 273 | histone H4 | 21361209 | 1 | 1.41 | 1.82 | 1.82 |
| 274 | electron transfer flavoprotein subunit beta | 38142460 | 1 | 1.41 | 1.82 | 1.82 |
| 275 | actin-related protein 2/3 complex subunit 1B | 160837788 | 1 | 1.41 | 1.82 | 1.82 |
| 276 | PREDICTED: nardilysin isoform X1 | 568928884 | 1 | 1.53 | 1.97 | 1.97 |
| 277 | protein AMBP precursor | 6680684 | 1 | 0.00 |  |  |
| 278 | ADP-ribosylation factor 5 | 6680722 | 1 | 0.00 |  |  |
| 279 | thioredoxin | 6755911 | 1 | 0.00 |  |  |
| 280 | tubulin alpha-8 chain | 8394493 | 1 | 0.00 |  |  |
| 281 | 1,4-alpha-glucan-branching enzyme | 17975508 | 1 | 0.00 |  |  |
| 282 | dihydropteridine reductase | 21312520 | 1 | 0.00 |  |  |
| 283 | osteoclast-stimulating factor 1 | 22267440 | 1 | 0.00 |  |  |
| 284 | alcohol dehydrogenase class-3 | 31982511 | 1 | 0.00 |  |  |
| 285 | ras-related C3 botulinum toxin substrate 1 precursor | 45592934 | 1 | 0.00 |  |  |
| 286 | PREDICTED: eukaryotic initiation factor 4A-III-like | 51712358 | 1 | 0.00 |  |  |
| 287 | glutathione reductase, mitochondrial precursor | 160298213 | 1 | 0.00 |  |  |
| 288 | nascent polypeptide-associated complex subunit alpha isoform a | 163965357 | 1 | 0.00 |  |  |
| 289 | histidine-rich glycoprotein precursor | 226958456 | 1 | 0.00 |  |  |
| 290 | PREDICTED: protein S100-A10 isoform X1 | 568922146 | 1 | 0.00 |  |  |
| 291 | PREDICTED: heterogeneous nuclear ribonucleoprotein H isoform X6 | 568975120 | 1 | 0.00 |  |  |
| 292 | PREDICTED: 40S ribosomal protein S10 isoform X2 | 569001407 | 1 | 0.00 |  |  |
| 293 | F-actin-capping protein subunit alpha-2 | 6671672 | 0.75 | 0.50 | 0.64 | 0.86 |
| 294 | 40S ribosomal protein S18 | 6755368 | 0.75 | 0.50 | 0.64 | 0.86 |
| 295 | PDZ and LIM domain protein 3 | 7948997 | 0.75 | 0.50 | 0.64 | 0.86 |
| 296 | leucine-rich alpha-2-glycoprotein precursor | 16418335 | 0.75 | 0.50 | 0.64 | 0.86 |
| 297 | heterogeneous nuclear ribonucleoprotein F | 19527048 | 0.75 | 0.50 | 0.64 | 0.86 |
| 298 | 40S ribosomal protein S28 | 21426821 | 0.75 | 0.50 | 0.64 | 0.86 |
| 299 | NAD(P)H-hydrate epimerase precursor | 21553309 | 0.75 | 0.50 | 0.64 | 0.86 |
| 300 | myosin regulatory light chain 12B | 21728376 | 0.75 | 0.50 | 0.64 | 0.86 |
| 301 | trans-1,2-dihydrobenzene-1,2-diol dehydrogenase | 27229131 | 0.75 | 0.50 | 0.64 | 0.86 |
| 302 | inorganic pyrophosphatase | 27754065 | 0.75 | 0.50 | 0.64 | 0.86 |
| 303 | enoyl-CoA hydratase, mitochondrial precursor | 29789289 | 0.75 | 0.50 | 0.64 | 0.86 |
| 304 | beta-2-microglobulin precursor | 31981890 | 0.75 | 0.50 | 0.64 | 0.86 |
| 305 | PREDICTED: 40S ribosomal protein S23-like | 94363969 | 0.75 | 0.50 | 0.64 | 0.86 |
| 306 | kelch-like protein 41 | 124487329 | 0.75 | 0.50 | 0.64 | 0.86 |
| 307 | cellular nucleic acid-binding protein isoform 3 | 157909784 | 0.75 | 0.50 | 0.64 | 0.86 |
| 308 | carbonic anhydrase 2 | 157951596 | 0.75 | 0.50 | 0.64 | 0.86 |
| 309 | heat shock protein beta-1 | 158937312 | 0.75 | 0.50 | 0.64 | 0.86 |
| 310 | serine/threonine-protein phosphatase 2A activator | 254587947 | 0.75 | 0.50 | 0.64 | 0.86 |
| 311 | succinyl-CoA ligase | 255958286 | 0.75 | 0.50 | 0.64 | 0.86 |
| 312 | PREDICTED: GMP reductase 1 isoform X1 | 568982539 | 0.75 | 0.50 | 0.64 | 0.86 |
| 313 | corticosteroid-binding globulin precursor | 6680856 | 0.75 | 0.96 | 1.23 | 1.64 |
| 314 | aldehyde dehydrogenase, mitochondrial precursor | 6753036 | 0.75 | 0.96 | 1.23 | 1.64 |
| 315 | maleylacetoacetate isomerase isoform 1 | 6754092 | 0.75 | 0.96 | 1.23 | 1.64 |
| 316 | endoplasmin precursor | 6755863 | 0.75 | 0.96 | 1.23 | 1.64 |
| 317 | purine nucleoside phosphorylase | 7305395 | 0.75 | 0.96 | 1.23 | 1.64 |
| 318 | actin-related protein 2/3 complex subunit 3 | 9790141 | 0.75 | 0.96 | 1.23 | 1.64 |
| 319 | eukaryotic initiation factor 4A-I isoform 1 | 21450625 | 0.75 | 0.96 | 1.23 | 1.64 |
| 320 | polyadenylate-binding protein 1 | 31560656 | 0.75 | 0.96 | 1.23 | 1.64 |
| 321 | long-chain specific acyl-CoA dehydrogenase, mitochondrial precursor | 31982520 | 0.75 | 0.96 | 1.23 | 1.64 |
| 322 | acidic leucine-rich nuclear phosphoprotein 32 family member A | 40254600 | 0.75 | 0.96 | 1.23 | 1.64 |
| 323 | glutathione peroxidase 1 | 84871986 | 0.75 | 0.96 | 1.23 | 1.64 |
| 324 | filamin-A | 125347376 | 0.75 | 0.96 | 1.23 | 1.64 |
| 325 | xaa-Pro dipeptidase | 170650724 | 0.75 | 0.96 | 1.23 | 1.64 |
| 326 | 40S ribosomal protein S5 | 254675270 | 0.75 | 0.96 | 1.23 | 1.64 |
| 327 | delta-aminolevulinic acid dehydratase | 451172117 | 0.75 | 0.96 | 1.23 | 1.64 |
| 328 | PREDICTED: septin-2 isoform X1 | 568908352 | 0.75 | 0.96 | 1.23 | 1.64 |
| 329 | PREDICTED: IQ motif containing GTPase activating protein 1 isoform X2 | 568948007 | 0.75 | 0.96 | 1.23 | 1.64 |
| 330 | PREDICTED: keratin, type II cytoskeletal 7 isoform X1 | 568991050 | 0.75 | 0.96 | 1.23 | 1.64 |
| 331 | PREDICTED: protein MEMO1 isoform X1 | 569002017 | 0.75 | 0.96 | 1.23 | 1.64 |
| 332 | PREDICTED: O-acetyl-ADP-ribose deacetylase MACROD1 isoform X1 | 569006163 | 0.75 | 0.96 | 1.23 | 1.64 |
| 333 | glutamate dehydrogenase 1, mitochondrial precursor | 6680027 | 0.5 | 0.58 | 0.74 | 1.49 |
| 334 | ADP-ribosylation factor 1 | 6680716 | 0.5 | 0.58 | 0.74 | 1.49 |
| 335 | proteasome subunit alpha type-6 | 6755198 | 0.5 | 0.58 | 0.74 | 1.49 |
| 336 | proteasome subunit beta type-3 | 6755202 | 0.5 | 0.58 | 0.74 | 1.49 |
| 337 | serine/arginine-rich splicing factor 2 | 6755478 | 0.5 | 0.58 | 0.74 | 1.49 |
| 338 | transgelin | 6755714 | 0.5 | 0.58 | 0.74 | 1.49 |
| 339 | vitronectin precursor | 6755987 | 0.5 | 0.58 | 0.74 | 1.49 |
| 340 | peptidyl-prolyl cis-trans isomerase FKBP3 | 7305061 | 0.5 | 0.58 | 0.74 | 1.49 |
| 341 | 60S acidic ribosomal protein P1 | 9256519 | 0.5 | 0.58 | 0.74 | 1.49 |
| 342 | 60S ribosomal protein L11 | 13385408 | 0.5 | 0.58 | 0.74 | 1.49 |
| 343 | histone H1.4 | 13430890 | 0.5 | 0.58 | 0.74 | 1.49 |
| 344 | isocitrate dehydrogenase | 18250284 | 0.5 | 0.58 | 0.74 | 1.49 |
| 345 | glycogen | 31560022 | 0.5 | 0.58 | 0.74 | 1.49 |
| 346 | elongation factor 1-beta | 31980922 | 0.5 | 0.58 | 0.74 | 1.49 |
| 347 | dihydrolipoyl dehydrogenase, mitochondrial precursor | 31982856 | 0.5 | 0.58 | 0.74 | 1.49 |
| 348 | myosin light polypeptide 6 | 33620739 | 0.5 | 0.58 | 0.74 | 1.49 |
| 349 | rab GDP dissociation inhibitor alpha | 33859560 | 0.5 | 0.58 | 0.74 | 1.49 |
| 350 | adenylate kinase 2, mitochondrial isoform b | 34328230 | 0.5 | 0.58 | 0.74 | 1.49 |
| 351 | E3 ubiquitin-protein ligase NEDD4 | 56699423 | 0.5 | 0.58 | 0.74 | 1.49 |
| 352 | parathymosin | 62460366 | 0.5 | 0.58 | 0.74 | 1.49 |
| 353 | adenylate kinase 2, mitochondrial isoform b | 109627652 | 0.5 | 0.58 | 0.74 | 1.49 |
| 354 | complement factor I precursor | 110347406 | 0.5 | 0.58 | 0.74 | 1.49 |
| 355 | selenoprotein P precursor | 110735408 | 0.5 | 0.58 | 0.74 | 1.49 |
| 356 | AP-2 complex subunit alpha-1 isoform b | 116256510 | 0.5 | 0.58 | 0.74 | 1.49 |
| 357 | proteasome subunit alpha type-2 | 134031994 | 0.5 | 0.58 | 0.74 | 1.49 |
| 358 | heterogeneous nuclear ribonucleoprotein U | 160333923 | 0.5 | 0.58 | 0.74 | 1.49 |
| 359 | aspartyl aminopeptidase isoform a | 161016822 | 0.5 | 0.58 | 0.74 | 1.49 |
| 360 | catechol O-methyltransferase | 161484634 | 0.5 | 0.58 | 0.74 | 1.49 |
| 361 | liver carboxylesterase 1 precursor | 162287349 | 0.5 | 0.58 | 0.74 | 1.49 |
| 362 | serum amyloid P-component precursor | 226958497 | 0.5 | 0.58 | 0.74 | 1.49 |
| 363 | citrate synthase-like protein | 269973935 | 0.5 | 0.58 | 0.74 | 1.49 |
| 364 | PREDICTED: 40S ribosomal protein S8-like | 568910331 | 0.5 | 0.58 | 0.74 | 1.49 |
| 365 | PREDICTED: acetyl-coenzyme A synthetase, cytoplasmic isoform X1 | 568917969 | 0.5 | 0.58 | 0.74 | 1.49 |
| 366 | PREDICTED: protein S100-A13 isoform X1 | 568922150 | 0.5 | 0.58 | 0.74 | 1.49 |
| 367 | PREDICTED: four and a half LIM domains protein 3 isoform X1 | 568928422 | 0.5 | 0.58 | 0.74 | 1.49 |
| 368 | PREDICTED: UPF0587 protein C1orf123 homolog isoform X2 | 568929845 | 0.5 | 0.58 | 0.74 | 1.49 |
| 369 | PREDICTED: ubiquitin carboxyl-terminal hydrolase 5 isoform X1 | 568941433 | 0.5 | 0.58 | 0.74 | 1.49 |
| 370 | PREDICTED: heterogeneous nuclear ribonucleoprotein L isoform X2 | 568944894 | 0.5 | 0.58 | 0.74 | 1.49 |
| 371 | PREDICTED: nucleosome assembly protein 1-like 4 isoform X3 | 568952682 | 0.5 | 0.58 | 0.74 | 1.49 |
| 372 | PREDICTED: platelet-activating factor acetylhydrolase IB subunit beta isoform X1 | 568958990 | 0.5 | 0.58 | 0.74 | 1.49 |
| 373 | PREDICTED: septin-7 isoform X1 | 568959211 | 0.5 | 0.58 | 0.74 | 1.49 |
| 374 | PREDICTED: proteasome subunit alpha type-6 isoform X1 | 568979680 | 0.5 | 0.58 | 0.74 | 1.49 |
| 375 | PREDICTED: carboxypeptidase N subunit 2 isoform X1 | 568996281 | 0.5 | 0.58 | 0.74 | 1.49 |
| 376 | destrin | 9790219 | 0.5 | 0.58 | 0.74 | 1.49 |
| 377 | ubiquitin-conjugating enzyme E2 N | 18017605 | 0.5 | 0.58 | 0.74 | 1.49 |
| 378 | BTB/POZ domain-containing protein KCTD12 | 123701966 | 0.5 | 0.58 | 0.74 | 1.49 |
| 379 | PREDICTED: nucleosome assembly protein 1-like 4 isoform X4 | 568952684 | 0.5 | 0.58 | 0.74 | 1.49 |
| 380 | protein phosphatase 1 regulatory subunit 7 | 12963569 | 0.25 | 0.50 | 0.64 | 2.58 |

*Arithmetic average number of spectral hits;

** Standard deviation of average number of spectral hits, *n*=4;

***99% confidence interval of average number of spectral hits
