# Supplementary material for: Whole Proteome Analysis of Mouse Lymph Nodes in Cutaneous Anthrax
Source: PLoS One. 2014 Oct 20;9(10):e110873. doi: 10.1371/journal.pone.0110873 (PMC4203832; doi:10.1371/journal.pone.0110873)
Supplement: Table S2 — Proteins identified in the soluble LN content of B. anthracis-challenged mice in comparison with uninfected mice. (DOCX) [file pone.0110873.s002.docx]

**Table S2.** **Proteins identified in the soluble LN content of *B. anthracis*-challenged mice in comparison with uninfected mice**

|  | **Protein** | **GI number** | **Spectral hits/sample** | | |
| --- | --- | --- | --- | --- | --- |
|  |  |  | **Control*** | **Infected**** | **Increase** |
| 1 | albumin | 163310765 | 214.50 | 375.75 | 161.25 |
| 2 | serine (or cysteine) proteinase inhibitor, clade A, member 3K | 148747546 | 24.50 | 53.63 | 29.13 |
| 3 | transferrin | 20330802 | 55.00 | 78.63 | 23.63 |
| 4 | complement component 3 | 28175786 | 16.50 | 33.38 | 16.88 |
| 5 | plasminogen | 257471003 | 3.00 | 17.63 | 14.63 |
| 6 | S100 calcium binding protein A8 (calgranulin A) | 7305453 | 0.00 | 12.50 | 12.50 |
| 7 | haptoglobin | 8850219 | 0.00 | 9.75 | 9.75 |
| 8 | pregnancy zone protein | 110347469 | 18.50 | 28.25 | 9.75 |
| 9 | myosin, heavy polypeptide 9, non-muscle isoform 1 | 114326446 | 3.50 | 13.00 | 9.50 |
| 10 | apolipoprotein A-I | 160333304 | 8.50 | 17.75 | 9.25 |
| 11 | actin, alpha 2, smooth muscle, aorta | 6671507 | 9.00 | 17.88 | 8.88 |
| 12 | actin, beta, cytoplasmic | 6671509 | 7.50 | 16.13 | 8.63 |
| 13 | ceruloplasmin isoform b | 110347564 | 2.50 | 10.38 | 7.88 |
| 14 | lymphocyte cytosolic protein 1 | 31543113 | 8.50 | 15.50 | 7.00 |
| 15 | tubulin, alpha 1a | 6678465 | 2.50 | 9.13 | 6.63 |
| 16 | coronin, actin binding protein 1A | 6753492 | 6.00 | 12.63 | 6.63 |
| 17 | transgelin 2 | 30519911 | 3.50 | 9.88 | 6.38 |
| 18 | annexin A1 | 124517663 | 4.50 | 10.88 | 6.38 |
| 19 | filamin, alpha | 125347376 | 0.00 | 6.25 | 6.25 |
| 20 | coagulation factor II (thrombin) | 6753798 | 0.00 | 6.00 | 6.00 |
| 21 | serine (or cysteine) proteinase inhibitor, clade A, member 1b | 76881807 | 12.50 | 18.25 | 5.75 |
| 22 | group specific component | 51172612 | 7.50 | 13.00 | 5.50 |
| 23 | serine (or cysteine) proteinase inhibitor, clade A, member 1a | 6678079 | 4.00 | 9.38 | 5.38 |
| 24 | hemopexin | 23956086 | 21.00 | 26.25 | 5.25 |
| 25 | hemoglobin, beta adult major chain | 31982300 | 11.50 | 16.50 | 5.00 |
| 26 | hemoglobin alpha 1 chain | 145301578 | 0.50 | 5.25 | 4.75 |
| 27 | heat shock protein 8 | 31981690 | 11.00 | 15.75 | 4.75 |
| 28 | eukaryotic translation elongation factor 1 alpha 1 | 126032329 | 14.00 | 18.75 | 4.75 |
| 29 | S100 calcium binding protein A9 (calgranulin B) | 6677837 | 0.00 | 4.50 | 4.50 |
| 30 | heat shock protein 1, beta | 40556608 | 5.50 | 10.00 | 4.50 |
| 31 | moesin | 70778915 | 5.50 | 10.00 | 4.50 |
| 32 | complement component factor H | 109627652 | 0.50 | 4.88 | 4.38 |
| 33 | CAP, adenylate cyclase-associated protein 1 | 157951604 | 0.00 | 4.00 | 4.00 |
| 34 | talin 1 | 227116327 | 1.00 | 5.00 | 4.00 |
| 35 | complement factor B | 218156289 | 1.50 | 5.50 | 4.00 |
| 36 | inter alpha-trypsin inhibitor, heavy chain 4 | 226531047 | 1.50 | 5.50 | 4.00 |
| 37 | histone 1, H2af | 30061379 | 4.00 | 8.00 | 4.00 |
| 38 | fatty acid binding protein 4, adipocyte | 14149635 | 0.00 | 3.88 | 3.88 |
| 39 | signal transducer and activator of transcription 1 | 114326482 | 39.00 | 42.88 | 3.88 |
| 40 | tropomyosin 3, gamma | 40254525 | 0.50 | 4.25 | 3.75 |
| 41 | nucleolin | 84875537 | 2.00 | 5.75 | 3.75 |
| 42 | eukaryotic translation elongation factor 1 alpha 2 | 6681273 | 5.50 | 9.13 | 3.63 |
| 43 | alpha-2-HS-glycoprotein | 7304875 | 6.00 | 9.63 | 3.63 |
| 44 | tubulin, beta 3 | 12963615 | 9.00 | 12.63 | 3.63 |
| 45 | thymosin, beta 4, X chromosome | 10946578 | 1.00 | 4.50 | 3.50 |
| 46 | eukaryotic translation elongation factor 2 | 33859482 | 9.50 | 13.00 | 3.50 |
| 47 | ubiquitin-activating enzyme E1, Chr X | 444189294 | 3.50 | 6.88 | 3.38 |
| 48 | esterase 1 | 247269929 | 4.00 | 7.25 | 3.25 |
| 49 | carbonic anhydrase 3 | 31982861 | 26.00 | 29.25 | 3.25 |
| 50 | guanine nucleotide binding protein (G protein), beta polypeptide 2 like 1 | 6680047 | 0.50 | 3.63 | 3.13 |
| 51 | apolipoprotein E | 163644329 | 2.00 | 5.13 | 3.13 |
| 52 | tubulin, beta | 21746161 | 3.00 | 6.13 | 3.13 |
| 53 | histone 1, H4b | 30061405 | 0.00 | 3.00 | 3.00 |
| 54 | heterogeneous nuclear ribonucleoprotein A/B isoform 2 | 6754222 | 0.00 | 2.88 | 2.88 |
| 55 | heat shock protein 1, alpha | 6754254 | 0.50 | 3.38 | 2.88 |
| 56 | leukotriene A4 hydrolase | 116734870 | 5.00 | 7.88 | 2.88 |
| 57 | IQ motif containing GTPase activating protein 1 | 242332572 | 0.50 | 3.25 | 2.75 |
| 58 | hypothetical protein LOC71775 | 21313642 | 0.00 | 2.63 | 2.63 |
| 59 | ARP3 actin-related protein 3 homolog | 23956222 | 1.50 | 4.13 | 2.63 |
| 60 | actinin, alpha 1 | 61097906 | 3.50 | 6.13 | 2.63 |
| 61 | peroxiredoxin 5 precursor | 6755114 | 0.00 | 2.50 | 2.50 |
| 62 | ribosomal protein S3 | 6755372 | 0.00 | 2.50 | 2.50 |
| 63 | chloride intracellular channel 1 | 15617203 | 0.00 | 2.50 | 2.50 |
| 64 | Rho, GDP dissociation inhibitor (GDI) beta | 33563236 | 0.00 | 2.50 | 2.50 |
| 65 | keratin 76 | 85701680 | 1.00 | 3.50 | 2.50 |
| 66 | fascin homolog 1, actin bundling protein | 113680348 | 1.50 | 4.00 | 2.50 |
| 67 | kininogen 1 | 12963497 | 0.00 | 2.38 | 2.38 |
| 68 | leucine-rich alpha-2-glycoprotein | 16418335 | 0.00 | 2.38 | 2.38 |
| 69 | phosphoglycerate mutase 1 | 114326546 | 2.50 | 4.88 | 2.38 |
| 70 | histone 2, H2ab | 119433657 | 5.00 | 7.38 | 2.38 |
| 71 | fibrinogen, gamma polypeptide | 19527078 | 1.00 | 3.25 | 2.25 |
| 72 | proteasome activator subunit 2 isoform 1 | 20137004 | 0.00 | 2.13 | 2.13 |
| 73 | heat shock 70kD protein 5 (glucose-regulated protein) | 254540166 | 0.50 | 2.63 | 2.13 |
| 74 | histidine-rich glycoprotein | 226958456 | 0.00 | 2.00 | 2.00 |
| 75 | histone 1, H2bh | 30061387 | 0.00 | 2.00 | 2.00 |
| 76 | apolipoprotein H | 160358825 | 0.00 | 2.00 | 2.00 |
| 77 | tumor rejection antigen gp96 | 6755863 | 0.00 | 1.88 | 1.88 |
| 78 | gelsolin | 28916693 | 0.00 | 1.88 | 1.88 |
| 79 | Rho GDP dissociation inhibitor (GDI) alpha | 31982030 | 0.00 | 1.88 | 1.88 |
| 80 | SAM domain- and HD domain-containing protein 1 | 213418055 | 2.50 | 4.38 | 1.88 |
| 81 | peroxiredoxin 6 | 6671549 | 0.00 | 1.75 | 1.75 |
| 82 | ribosomal protein S8 | 6677813 | 0.00 | 1.75 | 1.75 |
| 83 | serum amyloid A 1 | 6677843 | 0.50 | 2.25 | 1.75 |
| 84 | high mobility group box 2 | 6680229 | 0.50 | 2.25 | 1.75 |
| 85 | tubulin, alpha 1C | 6678469 | 0.00 | 1.63 | 1.63 |
| 86 | antioxidant protein 1 | 6753136 | 0.00 | 1.63 | 1.63 |
| 87 | high mobility group box 1 | 6754208 | 0.50 | 2.13 | 1.63 |
| 88 | LIM and SH3 protein 1 | 6754508 | 0.50 | 2.13 | 1.63 |
| 89 | WD repeat domain 1 | 6755995 | 0.50 | 2.13 | 1.63 |
| 90 | heterogeneous nuclear ribonucleoprotein U | 160333923 | 0.50 | 2.13 | 1.63 |
| 91 | serine (or cysteine) proteinase inhibitor, clade C (antithrombin), member 1 | 18252782 | 1.00 | 2.63 | 1.63 |
| 92 | coactosin-like 1 | 19482160 | 1.50 | 3.13 | 1.63 |
| 93 | heterogeneous nuclear ribonucleoprotein F | 19527048 | 1.50 | 3.13 | 1.63 |
| 94 | glyoxalase 1 | 165932331 | 2.00 | 3.63 | 1.63 |
| 95 | fibrinogen, B beta polypeptide | 33859809 | 2.50 | 4.13 | 1.63 |
| 96 | histone 1, H1d | 254588110 | 3.00 | 4.63 | 1.63 |
| 97 | dihydropyrimidinase-like 2 | 40254595 | 3.00 | 4.63 | 1.63 |
| 98 | peptidylprolyl isomerase A | 6679439 | 0.00 | 1.50 | 1.50 |
| 99 | cell division cycle 42 homolog | 6753364 | 0.00 | 1.50 | 1.50 |
| 100 | purine-nucleoside phosphorylase | 7305395 | 0.50 | 2.00 | 1.50 |
| 101 | stress-induced phosphoprotein 1 | 14389431 | 0.50 | 2.00 | 1.50 |
| 102 | clathrin, heavy polypeptide (Hc) | 51491845 | 1.00 | 2.50 | 1.50 |
| 103 | serine (or cysteine) proteinase inhibitor, clade A, member 6 | 6680856 | 0.00 | 1.38 | 1.38 |
| 104 | capping protein (actin filament) muscle Z-line, beta isoform b | 6753262 | 0.00 | 1.38 | 1.38 |
| 105 | chaperonin subunit 4 (delta) | 6753322 | 0.00 | 1.38 | 1.38 |
| 106 | tyrosine 3-monooxygenase/tryptophan 5-monooxygenase activation protein, zeta polypeptide | 6756041 | 0.50 | 1.88 | 1.38 |
| 107 | myosin, light polypeptide 1 | 29789016 | 0.50 | 1.88 | 1.38 |
| 108 | complement component factor i | 110347406 | 1.00 | 2.38 | 1.38 |
| 109 | glutathione S-transferase, mu 2 | 6680121 | 0.00 | 1.25 | 1.25 |
| 110 | prolyl endopeptidase | 6755152 | 0.00 | 1.25 | 1.25 |
| 111 | vitronectin | 6755987 | 0.00 | 1.25 | 1.25 |
| 112 | actin related protein 2/3 complex, subunit 3 | 9790141 | 0.00 | 1.25 | 1.25 |
| 113 | actinin alpha 4 | 11230802 | 0.00 | 1.25 | 1.25 |
| 114 | keratin 14 | 21489935 | 0.00 | 1.25 | 1.25 |
| 115 | copine I | 25141332 | 0.00 | 1.25 | 1.25 |
| 116 | NS1-associated protein 1 isoform 2 | 29788787 | 0.00 | 1.25 | 1.25 |
| 117 | UDP-N-acetylglucosamine pyrophosphorylase 1 | 30520375 | 0.00 | 1.25 | 1.25 |
| 118 | tropomyosin 4 | 47894398 | 0.00 | 1.25 | 1.25 |
| 119 | eukaryotic translation elongation factor 1 delta isoform b | 54287684 | 0.00 | 1.25 | 1.25 |
| 120 | gelsolin-like capping protein | 110227377 | 0.00 | 1.25 | 1.25 |
| 121 | apolipoprotein A-IV | 110347473 | 0.50 | 1.75 | 1.25 |
| 122 | adenine phosphoribosyl transferase | 118601013 | 1.00 | 2.25 | 1.25 |
| 123 | chaperonin subunit 5 (epsilon) | 6671702 | 0.00 | 1.13 | 1.13 |
| 124 | calreticulin | 6680836 | 0.00 | 1.13 | 1.13 |
| 125 | inter-alpha trypsin inhibitor, heavy chain 2 | 226874935 | 0.00 | 1.13 | 1.13 |
| 126 | paraoxonase 1 | 261823995 | 0.00 | 1.13 | 1.13 |
| 127 | annexin A3 | 160707925 | 0.00 | 1.13 | 1.13 |
| 128 | drebrin-like | 7304993 | 0.00 | 1.13 | 1.13 |
| 129 | enolase 2, gamma neuronal | 7305027 | 0.00 | 1.13 | 1.13 |
| 130 | phosphoglycerate mutase 2 | 9256624 | 0.00 | 1.13 | 1.13 |
| 131 | actin-related protein 2 | 22122825 | 0.00 | 1.13 | 1.13 |
| 132 | prosaposin | 225735645 | 0.00 | 1.13 | 1.13 |
| 133 | poly(A) binding protein, cytoplasmic 4 isoform 1 | 34419622 | 0.00 | 1.13 | 1.13 |
| 134 | 3-phosphoglycerate dehydrogenase | 52353955 | 0.00 | 1.13 | 1.13 |
| 135 | complement factor H-related protein B | 71361676 | 0.50 | 1.63 | 1.13 |
| 136 | histone 1, H1t | 112807207 | 1.00 | 2.13 | 1.13 |
| 137 | Sjogren syndrome antigen B | 6678143 | 0.00 | 1.00 | 1.00 |
| 138 | neutrophilic granule protein | 164519050 | 0.00 | 1.00 | 1.00 |
| 139 | orosomucoid 2 | 6754950 | 0.00 | 1.00 | 1.00 |
| 140 | proteasome (prosome, macropain) subunit, alpha type 4 | 6755196 | 0.00 | 1.00 | 1.00 |
| 141 | annexin A4 | 161016799 | 0.00 | 1.00 | 1.00 |
| 142 | destrin | 9790219 | 0.00 | 1.00 | 1.00 |
| 143 | eukaryotic translation initiation factor 4A1 | 21450625 | 0.00 | 1.00 | 1.00 |
| 144 | lactotransferrin | 31560677 | 0.00 | 1.00 | 1.00 |
| 145 | selenoprotein P precursor | 74271806 | 0.00 | 1.00 | 1.00 |
| 146 | karyopherin (importin) beta 1 | 88014720 | 0.50 | 1.50 | 1.00 |
| 147 | eukaryotic translation elongation factor 1 gamma | 110625979 | 0.50 | 1.50 | 1.00 |
| 148 | potassium channel tetramerization domain containing 12 | 123701966 | 0.50 | 1.50 | 1.00 |
| 149 | chaperonin subunit 2 (beta) | 126521835 | 1.00 | 2.00 | 1.00 |
| 150 | nucleophosmin 1 | 6679108 | 0.00 | 0.88 | 0.88 |
| 151 | chaperonin subunit 3 (gamma) | 6753320 | 0.00 | 0.88 | 0.88 |
| 152 | actin related protein 2/3 complex, subunit 5 | 224809382 | 0.00 | 0.88 | 0.88 |
| 153 | threonyl-tRNA synthetase | 27229277 | 0.00 | 0.88 | 0.88 |
| 154 | coagulation factor XIII, A1 subunit | 30578393 | 0.00 | 0.88 | 0.88 |
| 155 | ribosomal protein L4 | 30794450 | 0.00 | 0.88 | 0.88 |
| 156 | ras homolog gene family, member A | 31542143 | 0.00 | 0.88 | 0.88 |
| 157 | CNDP dipeptidase 2 (metallopeptidase M20 family) | 31981273 | 0.00 | 0.88 | 0.88 |
| 158 | heterogeneous nuclear ribonucleoprotein L | 183980004 | 0.00 | 0.88 | 0.88 |
| 159 | trifunctional enzyme subunit alpha, mitochondrial precursor | 33859811 | 0.00 | 0.88 | 0.88 |
| 160 | nascent polypeptide-associated complex alpha polypeptide | 41350312 | 0.00 | 0.88 | 0.88 |
| 161 | X-prolyl aminopeptidase (aminopeptidase P) 1, soluble | 406855427 | 0.00 | 0.88 | 0.88 |
| 162 | ribosomal protein L18 | 83699424 | 0.00 | 0.88 | 0.88 |
| 163 | serine (or cysteine) proteinase inhibitor, clade A, member 3G | 86476056 | 0.00 | 0.88 | 0.88 |
| 164 | keratin complex 2, basic, gene 17 | 124487419 | 0.50 | 1.38 | 0.88 |
| 165 | afamin | 125347464 | 1.50 | 2.38 | 0.88 |
| 166 | fetuin beta isoform 2 | 144226209 | 1.50 | 2.38 | 0.88 |
| 167 | capping protein (actin filament) muscle Z-line, alpha 2 | 6671672 | 0.00 | 0.75 | 0.75 |
| 168 | ribosomal protein S5 | 254675270 | 0.00 | 0.75 | 0.75 |
| 169 | serine (or cysteine) proteinase inhibitor, clade B, member 9 | 6678101 | 0.00 | 0.75 | 0.75 |
| 170 | peptidase D | 170650724 | 0.00 | 0.75 | 0.75 |
| 171 | granulin | 224967126 | 0.00 | 0.75 | 0.75 |
| 172 | dihydropyrimidinase-like 3 | 6681219 | 0.00 | 0.75 | 0.75 |
| 173 | chaperonin subunit 6a (zeta) | 6753324 | 0.00 | 0.75 | 0.75 |
| 174 | coronin, actin binding protein 1B | 6753494 | 0.00 | 0.75 | 0.75 |
| 175 | poly A binding protein, cytoplasmic 2 | 6754972 | 0.00 | 0.75 | 0.75 |
| 176 | ErbB3-binding protein 1 | 6755100 | 0.00 | 0.75 | 0.75 |
| 177 | tyrosine 3-monooxygenase/tryptophan 5-monooxygenase activation protein, eta polypeptide | 6756037 | 0.00 | 0.75 | 0.75 |
| 178 | nucleosome assembly protein 1-like 1 | 7657357 | 0.00 | 0.75 | 0.75 |
| 179 | alpha isoform of regulatory subunit A, protein phosphatase 2 | 8394027 | 0.00 | 0.75 | 0.75 |
| 180 | heterogeneous nuclear ribonucleoprotein H2 | 9845253 | 0.00 | 0.75 | 0.75 |
| 181 | lymphocyte specific 1 | 11225264 | 0.00 | 0.75 | 0.75 |
| 182 | actin related protein 2/3 complex, subunit 1B | 160837788 | 0.00 | 0.75 | 0.75 |
| 183 | protein phosphatase-1 regulatory subunit 7 | 12963569 | 0.00 | 0.75 | 0.75 |
| 184 | histone 1, H1e | 13430890 | 0.00 | 0.75 | 0.75 |
| 185 | complement component 9 | 15375312 | 0.00 | 0.75 | 0.75 |
| 186 | dipeptidyl peptidase III | 244791124 | 0.00 | 0.75 | 0.75 |
| 187 | histidine triad protein member 5 | 21312256 | 0.00 | 0.75 | 0.75 |
| 188 | acetyl-CoA acetyltransferase, mitochondrial | 21450129 | 0.00 | 0.75 | 0.75 |
| 189 | keratin 6L | 22164776 | 0.50 | 1.25 | 0.75 |
| 190 | valosin containing protein | 225543319 | 0.50 | 1.25 | 0.75 |
| 191 | hypothetical protein LOC238880 | 30425250 | 0.50 | 1.25 | 0.75 |
| 192 | peroxiredoxin 2 | 148747558 | 0.50 | 1.25 | 0.75 |
| 193 | eukaryotic translation elongation factor 1 beta 2 | 31980922 | 0.50 | 1.25 | 0.75 |
| 194 | chaperonin subunit 7 (eta) | 238814391 | 0.50 | 1.25 | 0.75 |
| 195 | kininogen 2 | 41235784 | 0.50 | 1.25 | 0.75 |
| 196 | prolyl 4-hydroxylase, beta polypeptide | 42415475 | 1.00 | 1.75 | 0.75 |
| 197 | KH-type splicing regulatory protein | 163954948 | 1.00 | 1.75 | 0.75 |
| 198 | serine (or cysteine) proteinase inhibitor, clade B, member 1a | 114158675 | 1.50 | 2.25 | 0.75 |
| 199 | phosphogluconate dehydrogenase | 124486895 | 2.00 | 2.75 | 0.75 |
| 200 | kallikrein B, plasma 1 | 236465805 | 0.00 | 0.63 | 0.63 |
| 201 | ATP synthase subunit alpha, mitochondrial precursor | 6680748 | 0.00 | 0.63 | 0.63 |
| 202 | epidermal growth factor receptor isoform 2 | 6681283 | 0.00 | 0.63 | 0.63 |
| 203 | guanine deaminase | 6753960 | 0.00 | 0.63 | 0.63 |
| 204 | proteasome (prosome, macropain) 28 subunit, alpha | 6755212 | 0.00 | 0.63 | 0.63 |
| 205 | RAN binding protein 1 | 153792001 | 0.00 | 0.63 | 0.63 |
| 206 | serum amyloid A 2 | 6755394 | 0.00 | 0.63 | 0.63 |
| 207 | serum amyloid P-component | 226958497 | 0.00 | 0.63 | 0.63 |
| 208 | carboxyl terminal LIM domain protein 1 | 158635992 | 0.00 | 0.63 | 0.63 |
| 209 | non-POU-domain-containing, octamer binding protein | 255958247 | 0.00 | 0.63 | 0.63 |
| 210 | heterogeneous nuclear ribonucleoprotein K | 13384620 | 0.00 | 0.63 | 0.63 |
| 211 | myoglobin | 21359820 | 0.00 | 0.63 | 0.63 |
| 212 | eosinophil-associated, ribonuclease A family, member 6 | 21426871 | 0.00 | 0.63 | 0.63 |
| 213 | histone 2, H2ac | 30089710 | 0.00 | 0.63 | 0.63 |
| 214 | vinculin | 31543942 | 0.00 | 0.63 | 0.63 |
| 215 | ribosomal protein S3a | 254553321 | 0.00 | 0.63 | 0.63 |
| 216 | proteasome (prosome, macropain) subunit, alpha type 3 | 261824000 | 0.00 | 0.63 | 0.63 |
| 217 | serine (or cysteine) proteinase inhibitor, clade A (alpha-1 antiproteinase, antitrypsin), member 10 | 31981720 | 0.00 | 0.63 | 0.63 |
| 218 | fibrinogen, alpha polypeptide | 33563252 | 0.00 | 0.63 | 0.63 |
| 219 | nucleoside-diphosphate kinase 1 | 37700232 | 0.00 | 0.63 | 0.63 |
| 220 | loss of heterozygosity, 11, chromosomal region 2, gene A homolog | 225543183 | 0.00 | 0.63 | 0.63 |
| 221 | carboxypeptidase N, polypeptide 2 homolog | 147904569 | 0.00 | 0.63 | 0.63 |
| 222 | S-adenosylhomocysteine hydrolase | 262263372 | 0.00 | 0.63 | 0.63 |
| 223 | staphylococcal nuclease domain containing 1 | 77404392 | 0.00 | 0.63 | 0.63 |
| 224 | coagulation factor X | 110625994 | 0.00 | 0.63 | 0.63 |
| 225 | actin related protein 2/3 complex, subunit 2 | 112363072 | 0.00 | 0.63 | 0.63 |
| 226 | spectrin alpha 2 | 115496850 | 0.00 | 0.63 | 0.63 |
| 227 | guanosine diphosphate (GDP) dissociation inhibitor 2 | 116089273 | 0.50 | 1.13 | 0.63 |
| 228 | protein tyrosine phosphatase, non-receptor type 6 isoform a | 118130771 | 0.50 | 1.13 | 0.63 |
| 229 | alpha-1-B glycoprotein | 124486702 | 0.50 | 1.13 | 0.63 |
| 230 | ribosomal protein L19 | 226958653 | 0.00 | 0.50 | 0.50 |
| 231 | microsomal triglyceride transfer protein | 254540223 | 0.00 | 0.50 | 0.50 |
| 232 | serine (or cysteine) proteinase inhibitor, clade A, member 3C | 6680586 | 0.00 | 0.50 | 0.50 |
| 233 | cathepsin B preproprotein | 6681079 | 0.00 | 0.50 | 0.50 |
| 234 | alcohol dehydrogenase 1 (class I) | 6724311 | 0.00 | 0.50 | 0.50 |
| 235 | annexin A5 | 6753060 | 0.00 | 0.50 | 0.50 |
| 236 | septin 2 | 6754816 | 0.00 | 0.50 | 0.50 |
| 237 | protein C receptor, endothelial | 251823822 | 0.00 | 0.50 | 0.50 |
| 238 | retinol binding protein 1, cellular | 6755300 | 0.00 | 0.50 | 0.50 |
| 239 | transgelin | 6755714 | 0.00 | 0.50 | 0.50 |
| 240 | argininosuccinate synthetase | 6996911 | 0.00 | 0.50 | 0.50 |
| 241 | actinin alpha 3 | 7304855 | 0.00 | 0.50 | 0.50 |
| 242 | alpha-2-glycoprotein 1, zinc | 160415217 | 0.00 | 0.50 | 0.50 |
| 243 | eukaryotic translation initiation factor 4A2 | 176865892 | 0.00 | 0.50 | 0.50 |
| 244 | ubiquitin A-52 residue ribosomal protein fusion product 1 | 9845265 | 0.00 | 0.50 | 0.50 |
| 245 | thioredoxin-like 5 | 13386060 | 0.00 | 0.50 | 0.50 |
| 246 | SH3 domain binding glutamic acid-rich protein-like 3 | 18017602 | 0.00 | 0.50 | 0.50 |
| 247 | farnesyl diphosphate synthetase | 19882207 | 0.00 | 0.50 | 0.50 |
| 248 | glyoxalase domain containing 4 | 255003777 | 0.00 | 0.50 | 0.50 |
| 249 | eosinophil-associated, ribonuclease A family, member 10 | 21426867 | 0.00 | 0.50 | 0.50 |
| 250 | DEAD (Asp-Glu-Ala-Asp) box polypeptide 3, Y-linked | 25141235 | 0.00 | 0.50 | 0.50 |
| 251 | copine III | 25141335 | 0.00 | 0.50 | 0.50 |
| 252 | asparaginyl-tRNA synthetase | 219275596 | 0.00 | 0.50 | 0.50 |
| 253 | tyrosine 3-monooxygenase/tryptophan 5-monooxygenase activation protein, beta polypeptide | 31543974 | 0.00 | 0.50 | 0.50 |
| 254 | long-chain-fatty-acid--CoA ligase 1 | 31560705 | 0.00 | 0.50 | 0.50 |
| 255 | ribonuclease/angiogenin inhibitor 1 | 31981748 | 0.00 | 0.50 | 0.50 |
| 256 | beta-2-microglobulin | 31981890 | 0.00 | 0.50 | 0.50 |
| 257 | tyrosine 3-monooxygenase/tryptophan 5-monooxygenase activation protein, epsilon polypeptide | 226874906 | 0.00 | 0.50 | 0.50 |
| 258 | alanyl-tRNA synthetase | 34610207 | 0.00 | 0.50 | 0.50 |
| 259 | ribosomal protein, large P2 | 83745120 | 0.50 | 1.00 | 0.50 |
| 260 | glutathione peroxidase 1 | 84871986 | 0.50 | 1.00 | 0.50 |
| 261 | thioredoxin reductase 1 isoform 2 | 110224442 | 0.50 | 1.00 | 0.50 |
| 262 | t-complex protein 1 | 110625624 | 0.50 | 1.00 | 0.50 |
| 263 | protein C | 347543804 | 0.50 | 1.00 | 0.50 |
| 264 | carboxylesterase 3 | 117553604 | 0.50 | 1.00 | 0.50 |
| 265 | sorcin isoform 1 | 124430537 | 0.50 | 1.00 | 0.50 |
| 266 | talin 2 | 163310736 | 1.00 | 1.50 | 0.50 |
| 267 | liver carboxylesterase N-like | 124487013 | 1.50 | 2.00 | 0.50 |
| 268 | solute carrier family 30 (zinc transporter), member 9 | 125660458 | 1.50 | 2.00 | 0.50 |
| 269 | lectin, galactose binding, soluble 1 | 6678682 | 0.00 | 0.38 | 0.38 |
| 270 | lysophospholipase 1 | 6678760 | 0.00 | 0.38 | 0.38 |
| 271 | pyruvate carboxylase | 251823978 | 0.00 | 0.38 | 0.38 |
| 272 | serine (or cysteine) proteinase inhibitor, clade F, member 2 | 6679383 | 0.00 | 0.38 | 0.38 |
| 273 | myotrophin | 6679961 | 0.00 | 0.38 | 0.38 |
| 274 | H3 histone, family 3B | 6680161 | 0.00 | 0.38 | 0.38 |
| 275 | calponin 2 | 6680952 | 0.00 | 0.38 | 0.38 |
| 276 | alpha-crystallin B chain | 6753530 | 0.00 | 0.38 | 0.38 |
| 277 | fibulin 1 | 168693628 | 0.00 | 0.38 | 0.38 |
| 278 | proteasome (prosome, macropain) 26S subunit, non-ATPase, 7 | 6754724 | 0.00 | 0.38 | 0.38 |
| 279 | proteosome (prosome, macropain) subunit, beta type 8 (large multifunctional protease 7) | 158303322 | 0.00 | 0.38 | 0.38 |
| 280 | phosphoenolpyruvate carboxykinase, cytosolic | 7110683 | 0.00 | 0.38 | 0.38 |
| 281 | proteasome (prosome, macropain) subunit, beta type 1 | 7242197 | 0.00 | 0.38 | 0.38 |
| 282 | cellular nucleic acid binding protein | 7304969 | 0.00 | 0.38 | 0.38 |
| 283 | lactate dehydrogenase C | 7305229 | 0.00 | 0.38 | 0.38 |
| 284 | metallothionein 1 | 7305285 | 0.00 | 0.38 | 0.38 |
| 285 | myosin, heavy polypeptide 11, smooth muscle | 7305295 | 0.00 | 0.38 | 0.38 |
| 286 | proteasome (prosome, macropain) subunit, beta type 10 | 247300942 | 0.00 | 0.38 | 0.38 |
| 287 | cell division cycle 37 homolog | 7949018 | 0.00 | 0.38 | 0.38 |
| 288 | lysozyme | 8393739 | 0.00 | 0.38 | 0.38 |
| 289 | coatomer protein complex, subunit gamma 2 | 8567340 | 0.00 | 0.38 | 0.38 |
| 290 | eukaryotic translation initiation factor 3, subunit 2 (beta) | 9055370 | 0.00 | 0.38 | 0.38 |
| 291 | phosphofructokinase, platelet | 9790051 | 0.00 | 0.38 | 0.38 |
| 292 | HLA-B-associated transcript 1A | 9790069 | 0.00 | 0.38 | 0.38 |
| 293 | protein arginine N-methyltransferase 1 | 9790109 | 0.00 | 0.38 | 0.38 |
| 294 | minichromosome maintenance complex component 7 | 10242373 | 0.00 | 0.38 | 0.38 |
| 295 | creatine kinase, brain | 10946574 | 0.00 | 0.38 | 0.38 |
| 296 | ribosomal protein L23 | 12584986 | 0.00 | 0.38 | 0.38 |
| 297 | 6-phosphogluconolactonase | 13384778 | 0.00 | 0.38 | 0.38 |
| 298 | protein Z, vitamin K-dependent plasma glycoprotein | 13385306 | 0.00 | 0.38 | 0.38 |
| 299 | small nuclear ribonucleoprotein D3 | 13385598 | 0.00 | 0.38 | 0.38 |
| 300 | acidic nuclear phosphoprotein 32 family, member B | 18700032 | 0.00 | 0.38 | 0.38 |
| 301 | endoplasmic reticulum protein ERp29 precursor | 19526463 | 0.00 | 0.38 | 0.38 |
| 302 | suppression of tumorigenicity 13 | 19526912 | 0.00 | 0.38 | 0.38 |
| 303 | arginyl-tRNA synthetase | 262118273 | 0.00 | 0.38 | 0.38 |
| 304 | poly (ADP-ribose) polymerase family, member 1 | 20806109 | 0.00 | 0.38 | 0.38 |
| 305 | keratin 5 | 20911031 | 0.00 | 0.38 | 0.38 |
| 306 | actin related protein 2/3 complex, subunit 5-like | 21312654 | 0.00 | 0.38 | 0.38 |
| 307 | hypothetical protein LOC223601 | 21450053 | 0.00 | 0.38 | 0.38 |
| 308 | cysteine sulfinic acid decarboxylase | 21450351 | 0.00 | 0.38 | 0.38 |
| 309 | methionine adenosyltransferase II, alpha | 21704144 | 0.00 | 0.38 | 0.38 |
| 310 | osteoclast stimulating factor 1 | 22267440 | 0.00 | 0.38 | 0.38 |
| 311 | Nice-4 protein homolog isoform 2 | 260166709 | 0.00 | 0.38 | 0.38 |
| 312 | integrin beta 4 binding protein | 27501448 | 0.00 | 0.38 | 0.38 |
| 313 | ribulose-5-phosphate-3-epimerase | 27532955 | 0.00 | 0.38 | 0.38 |
| 314 | abhydrolase domain containing 14b | 27753960 | 0.00 | 0.38 | 0.38 |
| 315 | coatomer protein complex, subunit beta 2 (beta prime) | 29789080 | 0.00 | 0.38 | 0.38 |
| 316 | histone 2, H3b | 30061347 | 0.00 | 0.38 | 0.38 |
| 317 | hepatoma-derived growth factor | 188497724 | 0.00 | 0.38 | 0.38 |
| 318 | protein phosphatase 2A, regulatory subunit B (PR 53) | 254587947 | 0.00 | 0.38 | 0.38 |
| 319 | seryl-aminoacyl-tRNA synthetase | 33468931 | 0.00 | 0.38 | 0.38 |
| 320 | ribosomal protein S9-like | 33504483 | 0.00 | 0.38 | 0.38 |
| 321 | keratin 84 | 225903444 | 0.00 | 0.38 | 0.38 |
| 322 | lipocalin 2 | 34328049 | 0.00 | 0.38 | 0.38 |
| 323 | RNA binding motif protein 3 | 37497112 | 0.00 | 0.38 | 0.38 |
| 324 | acidic (leucine-rich) nuclear phosphoprotein 32 family, member A | 40254600 | 0.00 | 0.38 | 0.38 |
| 325 | aminopeptidase-like 1 | 47523981 | 0.00 | 0.38 | 0.38 |
| 326 | PREDICTED: hypothetical protein | 51772073 | 0.00 | 0.38 | 0.38 |
| 327 | proteasome 26S non-ATPase subunit 1 | 74315975 | 0.00 | 0.38 | 0.38 |
| 328 | exportin 1, CRM1 homolog | 78190507 | 0.00 | 0.38 | 0.38 |
| 329 | villin 2 | 83921618 | 0.00 | 0.38 | 0.38 |
| 330 | heterogeneous nuclear ribonucleoprotein A1 isoform b | 85060507 | 0.00 | 0.38 | 0.38 |
| 331 | C-reactive protein, petaxin related | 162138926 | 0.00 | 0.38 | 0.38 |
| 332 | phosphoribosylglycinamide formyltransferase | 93102415 | 0.00 | 0.38 | 0.38 |
| 333 | PREDICTED: similar to yeast ribosomal protein S28 homologue | 94363969 | 0.50 | 0.88 | 0.38 |
| 334 | hypoxanthine guanine phosphoribosyl transferase 1 | 96975138 | 0.50 | 0.88 | 0.38 |
| 335 | angiotensinogen | 113461998 | 0.50 | 0.88 | 0.38 |
| 336 | hydroxysteroid dehydrogenase-5, delta<5>-3-beta | 113680667 | 0.50 | 0.88 | 0.38 |
| 337 | eukaryotic translation initiation factor 5 | 124430541 | 0.50 | 0.88 | 0.38 |
| 338 | keratin complex 2, basic, gene 1 | 126116585 | 0.50 | 0.88 | 0.38 |
| 339 | ribosomal protein L12 | 160333553 | 0.00 | 0.25 | 0.25 |
| 340 | ribosomal protein L22 | 6677775 | 0.00 | 0.25 | 0.25 |
| 341 | proteasome (prosome, macropain) 26S subunit, ATPase 3 | 228008337 | 0.00 | 0.25 | 0.25 |
| 342 | RAB7, member RAS oncogene family | 148747526 | 0.00 | 0.25 | 0.25 |
| 343 | RAS-related C3 botulinum substrate 2 | 6679601 | 0.00 | 0.25 | 0.25 |
| 344 | interferon gamma inducible protein 47 | 6680359 | 0.00 | 0.25 | 0.25 |
| 345 | inositol polyphosphate-1-phosphatase | 170650609 | 0.00 | 0.25 | 0.25 |
| 346 | ADP-ribosylation factor 3 | 6680718 | 0.00 | 0.25 | 0.25 |
| 347 | ADP-ribosylation factor 5 | 6680722 | 0.00 | 0.25 | 0.25 |
| 348 | eosinophil-associated, ribonuclease A family, member 2 | 6681251 | 0.00 | 0.25 | 0.25 |
| 349 | annexin A7 | 160707956 | 0.00 | 0.25 | 0.25 |
| 350 | serine (or cysteine) proteinase inhibitor, clade G, member 1 | 163914390 | 0.00 | 0.25 | 0.25 |
| 351 | serine (or cysteine) proteinase inhibitor, clade H, member 1 | 161353502 | 0.00 | 0.25 | 0.25 |
| 352 | chitinase 3-like 3 | 254281348 | 0.00 | 0.25 | 0.25 |
| 353 | DEAD/H (Asp-Glu-Ala-Asp/His) box polypeptide 3, X-linked | 6753620 | 0.00 | 0.25 | 0.25 |
| 354 | macrophage migration inhibitory factor | 6754696 | 0.00 | 0.25 | 0.25 |
| 355 | serum amyloid A 4 | 6755398 | 0.00 | 0.25 | 0.25 |
| 356 | sepiapterin reductase | 160333789 | 0.00 | 0.25 | 0.25 |
| 357 | translin | 6755899 | 0.00 | 0.25 | 0.25 |
| 358 | keratin 17 | 7106335 | 0.00 | 0.25 | 0.25 |
| 359 | tubulin, beta 5 | 7106439 | 0.00 | 0.25 | 0.25 |
| 360 | protease (prosome, macropain) 26S subunit, ATPase 5 | 7110703 | 0.00 | 0.25 | 0.25 |
| 361 | chloride intracellular channel 4 (mitochondrial) | 7304963 | 0.00 | 0.25 | 0.25 |
| 362 | leukemia inhibitory factor receptor | 7305235 | 0.00 | 0.25 | 0.25 |
| 363 | cytidine 5'-triphosphate synthase | 172072613 | 0.00 | 0.25 | 0.25 |
| 364 | ribosomal protein, large, P1 | 9256519 | 0.00 | 0.25 | 0.25 |
| 365 | SERPINE1 mRNA binding protein 1 | 165932375 | 0.00 | 0.25 | 0.25 |
| 366 | basic leucine zipper and W2 domains 1 | 13385296 | 0.00 | 0.25 | 0.25 |
| 367 | glucose-6-phosphate dehydrogenase 2 | 227330582 | 0.00 | 0.25 | 0.25 |
| 368 | serine (or cysteine) proteinase inhibitor, clade B, member 6b | 15826844 | 0.00 | 0.25 | 0.25 |
| 369 | pyruvate dehydrogenase E1 component subunit beta | 18152793 | 0.00 | 0.25 | 0.25 |
| 370 | hypothetical protein LOC70984 | 19526926 | 0.00 | 0.25 | 0.25 |
| 371 | RANBP4 | 19745156 | 0.00 | 0.25 | 0.25 |
| 372 | deoxyuridine triphosphatase | 21281687 | 0.00 | 0.25 | 0.25 |
| 373 | N-acetylneuraminate pyruvate lyase | 21311855 | 0.00 | 0.25 | 0.25 |
| 374 | histone 1, H1b | 21426893 | 0.00 | 0.25 | 0.25 |
| 375 | very long-chain specific acyl-CoA dehydrogenase | 23956084 | 0.00 | 0.25 | 0.25 |
| 376 | UNC-112 related protein 2 | 24418903 | 0.00 | 0.25 | 0.25 |
| 377 | brain-specific angiogenesis inhibitor 2 | 27414503 | 0.00 | 0.25 | 0.25 |
| 378 | proteasome 26S ATPase subunit 6 | 27754103 | 0.00 | 0.25 | 0.25 |
| 379 | aspartyl-tRNA synthetase | 211065507 | 0.00 | 0.25 | 0.25 |
| 380 | RAN binding protein 5 | 29789199 | 0.00 | 0.25 | 0.25 |
| 381 | eukaryotic translation initiation factor 3, subunit 9 | 29789343 | 0.00 | 0.25 | 0.25 |
| 382 | bleomycin hydrolase | 30519997 | 0.00 | 0.25 | 0.25 |
| 383 | coronin, actin binding protein 1C | 31542413 | 0.00 | 0.25 | 0.25 |
| 384 | enoyl Coenzyme A hydratase domain containing 1 | 31542451 | 0.00 | 0.25 | 0.25 |
| 385 | ELAV (embryonic lethal, abnormal vision, Drosophila)-like 1 (Hu antigen R) | 31542602 | 0.00 | 0.25 | 0.25 |
| 386 | latexin | 31980632 | 0.00 | 0.25 | 0.25 |
| 387 | ribosomal protein S14 | 31981100 | 0.00 | 0.25 | 0.25 |
| 388 | proteasome (prosome, macropain) subunit, beta type 2 | 227116345 | 0.00 | 0.25 | 0.25 |
| 389 | ribosomal protein L7 | 31981515 | 0.00 | 0.25 | 0.25 |
| 390 | histidyl-tRNA synthetase | 251823891 | 0.00 | 0.25 | 0.25 |
| 391 | MO25 protein |  | 0.00 | 0.25 | 0.25 |
| 392 | WSB-2 protein | 31982686 | 0.00 | 0.25 | 0.25 |
| 393 | proteasome (prosome, macropain) subunit, alpha type 1 | 33563282 | 0.00 | 0.25 | 0.25 |
| 394 | complement component 8, beta subunit | 33563297 | 0.00 | 0.25 | 0.25 |
| 395 | dynein, axonemal, heavy chain 8 | 153792273 | 0.00 | 0.25 | 0.25 |
| 396 | dosage compensation-related protein DPY30 | 226246654 | 0.00 | 0.25 | 0.25 |
| 397 | neutrophil cytosolic factor 1 | 170172553 | 0.00 | 0.25 | 0.25 |
| 398 | eukaryotic translation initiation factor 3, subunit 6 | 45476573 | 0.00 | 0.25 | 0.25 |
| 399 | methylthioadenosine phosphorylase | 45544618 | 0.00 | 0.25 | 0.25 |
| 400 | superoxide dismutase 1, soluble | 45597447 | 0.00 | 0.25 | 0.25 |
| 401 | succinate dehydrogenase | 54607098 | 0.00 | 0.25 | 0.25 |
| 402 | prefoldin 5 | 55741463 | 0.00 | 0.25 | 0.25 |
| 403 | eukaryotic translation initiation factor 4, gamma 1 isoform b | 56699434 | 0.00 | 0.25 | 0.25 |
| 404 | parathymosin | 62460366 | 0.00 | 0.25 | 0.25 |
| 405 | twinfilin 1 | 62990169 | 0.00 | 0.25 | 0.25 |
| 406 | sulfhydryl oxidase 1 isoform a precursor | 68131562 | 0.00 | 0.25 | 0.25 |
| 407 | trypsinogen 7 | 71043961 | 0.00 | 0.25 | 0.25 |
| 408 | xanthine dehydrogenase/oxidase | 77682555 | 0.00 | 0.25 | 0.25 |
| 409 |  | 82886628 | 0.00 | 0.25 | 0.25 |
| 410 | ubiquitin specific protease 14 isoform 2 | 84452155 | 0.00 | 0.25 | 0.25 |
| 411 | hypothetical protein LOC102502 | 85986577 | 0.00 | 0.25 | 0.25 |
| 412 | DEAD box polypeptide 17 isoform 4 | 93587673 | 0.00 | 0.25 | 0.25 |
| 413 | cytoplasmic aconitate hydratase | 110347487 | 0.00 | 0.25 | 0.25 |
| 414 | minichromosome maintenance deficient 5, cell division cycle 46 | 112293273 | 0.00 | 0.25 | 0.25 |
| 415 | heat shock protein 105 | 114145505 | 0.50 | 0.75 | 0.25 |
| 416 | heterogeneous nuclear ribonucleoprotein D isoform a | 116256512 | 0.50 | 0.75 | 0.25 |
| 417 | eukaryotic translation termination factor 1 | 124286826 | 0.50 | 0.75 | 0.25 |
| 418 | proteasome (prosome, macropain) subunit, alpha type 2 | 134031994 | 0.50 | 0.75 | 0.25 |
| 419 | dynein, cytoplasmic, heavy chain 1 | 134288917 | 0.50 | 0.75 | 0.25 |
| 420 | alpha 1 microglobulin/bikunin | 6680684 | 0.50 | 0.63 | 0.13 |
| 421 | calmodulin 2 | 6680832 | 0.50 | 0.63 | 0.13 |
| 422 | bridging integrator 1 isoform 1 | 6753050 | 0.50 | 0.63 | 0.13 |
| 423 | guanylate nucleotide binding protein 2 | 6753950 | 0.50 | 0.63 | 0.13 |
| 424 | proteasome (prosome, macropain) subunit, alpha type 6 | 6755198 | 0.50 | 0.63 | 0.13 |
| 425 | hemoglobin, beta adult minor chain | 17647499 | 0.50 | 0.63 | 0.13 |
| 426 | cell division cycle 10 homolog | 28173550 | 0.50 | 0.63 | 0.13 |
| 427 | eukaryotic translation initiation factor 5A2 | 29243942 | 0.50 | 0.63 | 0.13 |
| 428 | acid phosphatase 1, soluble | 31542070 | 0.50 | 0.63 | 0.13 |
| 429 | histidine triad nucleotide binding protein 1 | 33468857 | 0.50 | 0.63 | 0.13 |
| 430 | proteasome (prosome, macropain) 26S subunit, ATPase 2 | 33859604 | 0.50 | 0.63 | 0.13 |
| 431 | electron transferring flavoprotein, beta polypeptide | 38142460 | 1.00 | 1.13 | 0.13 |
| 432 | creatine kinase, mitochondrial 2 | 38259206 | 1.00 | 1.13 | 0.13 |
| 433 | myosin light chain, regulatory B-like | 71037403 | 1.00 | 1.13 | 0.13 |
| 434 | peroxiredoxin 1 | 6754976 | 0.50 | 0.50 | 0.00 |
| 435 | ubiquitin specific protease 5 (isopeptidase T) | 7305619 | 0.50 | 0.50 | 0.00 |
| 436 | SH3-binding domain glutamic acid-rich protein like | 9910548 | 0.50 | 0.50 | 0.00 |
| 437 | pigpen | 20982845 | 0.50 | 0.50 | 0.00 |
| 438 | dual specificity phosphatase 3 (vaccinia virus phosphatase VH1-related) | 21312314 | 0.50 | 0.50 | 0.00 |
| 439 | acylphosphatase 2, muscle type | 27229219 | 0.50 | 0.50 | 0.00 |
| 440 | alpha-2-macroglobulin | 148277039 | 0.50 | 0.50 | 0.00 |
| 441 | proline synthetase co-transcribed isoform b | 84872182 | 0.50 | 0.50 | 0.00 |
| 442 | glycyl-tRNA synthetase | 93102417 | 0.50 | 0.50 | 0.00 |
| 443 | heat shock protein 4 | 112293266 | 1.00 | 1.00 | 0.00 |
| 444 | adaptor protein complex AP-2, alpha 1 subunit isoform b | 116256510 | 1.00 | 1.00 | 0.00 |
| 445 | S100 calcium binding protein A13 | 347582617 | 0.50 | 0.38 | -0.13 |
| 446 | indolethylamine N-methyltransferase | 6678281 | 0.50 | 0.38 | -0.13 |
| 447 | cofilin 1, non-muscle | 6680924 | 0.50 | 0.38 | -0.13 |
| 448 | catechol-O-methyltransferase | 161484634 | 0.50 | 0.38 | -0.13 |
| 449 | cysteine and glycine-rich protein 1 | 6681069 | 0.50 | 0.38 | -0.13 |
| 450 | thioredoxin 1 | 6755911 | 0.50 | 0.38 | -0.13 |
| 451 | apolipoprotein C-III | 15421856 | 0.50 | 0.38 | -0.13 |
| 452 | ubiquitin carboxyl-terminal esterase L4 | 15809026 | 0.50 | 0.38 | -0.13 |
| 453 | myosin binding protein C, fast-type | 268370244 | 0.50 | 0.38 | -0.13 |
| 454 | tubulin, beta 2c | 22165384 | 0.50 | 0.38 | -0.13 |
| 455 | acetyl-Coenzyme A acyltransferase 2 (mitochondrial 3-oxoacyl-Coenzyme A thiolase) | 29126205 | 0.50 | 0.38 | -0.13 |
| 456 | hypothetical protein 4732456N10 | 269914154 | 0.50 | 0.38 | -0.13 |
| 457 | acidic (leucine-rich) nuclear phosphoprotein 32 family, member E | 254587996 | 0.50 | 0.38 | -0.13 |
| 458 | poly A binding protein, cytoplasmic 1 | 31560656 | 0.50 | 0.38 | -0.13 |
| 459 | inositol (myo)-1(or 4)-monophosphatase 1 | 31980942 | 1.00 | 0.88 | -0.13 |
| 460 | leucine aminopeptidase 3 | 255069715 | 1.00 | 0.88 | -0.13 |
| 461 | alcohol dehydrogenase 5 (class III), chi polypeptide | 31982511 | 1.50 | 1.38 | -0.13 |
| 462 | p47 protein | 38198665 | 1.50 | 1.38 | -0.13 |
| 463 | serine (or cysteine) proteinase inhibitor, clade F, member 1 | 117606335 | 1.50 | 1.38 | -0.13 |
| 464 | malic enzyme, supernatant | 162139827 | 0.50 | 0.25 | -0.25 |
| 465 | pyruvate dehydrogenase E1 alpha 1 | 6679261 | 0.50 | 0.25 | -0.25 |
| 466 | catalase | 157951741 | 0.50 | 0.25 | -0.25 |
| 467 | lactate dehydrogenase A | 6754524 | 0.50 | 0.25 | -0.25 |
| 468 | adipsin | 7304867 | 0.50 | 0.25 | -0.25 |
| 469 | FK506 binding protein 3 | 7305061 | 0.50 | 0.25 | -0.25 |
| 470 | small nuclear ribonucleoprotein N | 7305509 | 0.50 | 0.25 | -0.25 |
| 471 | esterase D/formylglutathione hydrolase | 13937355 | 0.50 | 0.25 | -0.25 |
| 472 | protease, serine, 1 | 16716569 | 0.50 | 0.25 | -0.25 |
| 473 | GTP-binding protein PTD004 isoform a | 21313144 | 0.50 | 0.25 | -0.25 |
| 474 | biliverdin reductase B (flavin reductase (NADPH)) | 21450325 | 0.50 | 0.25 | -0.25 |
| 475 | apolipoprotein A-I binding protein | 21553309 | 0.50 | 0.25 | -0.25 |
| 476 | annexin A6 | 31981302 | 0.50 | 0.25 | -0.25 |
| 477 | electron transferring flavoprotein, alpha polypeptide | 227500281 | 0.50 | 0.25 | -0.25 |
| 478 | bifunctional epoxide hydrolase 2 isoform a | 31982393 | 0.50 | 0.25 | -0.25 |
| 479 | calpastatin | 33563246 | 0.50 | 0.25 | -0.25 |
| 480 | transaldolase 1 | 33859640 | 1.00 | 0.75 | -0.25 |
| 481 | platelet-activating factor acetylhydrolase, isoform 1b, alpha2 subunit | 40254624 | 1.00 | 0.75 | -0.25 |
| 482 | phosphatidylethanolamine binding protein 1 | 84794552 | 1.00 | 0.75 | -0.25 |
| 483 | nudix (nucleoside diphosphate linked moiety X)-type motif 2 | 86198335 | 1.00 | 0.75 | -0.25 |
| 484 | glycosylphosphatidylinositol specific phospholipase D1 | 111378397 | 1.00 | 0.75 | -0.25 |
| 485 | keratin complex 1, acidic, gene 10 | 112983636 | 2.00 | 1.75 | -0.25 |
| 486 | amylo-1,6-glucosidase, 4-alpha-glucanotransferase | 124486747 | 2.00 | 1.75 | -0.25 |
| 487 | radixin | 157277948 | 0.50 | 0.13 | -0.38 |
| 488 | S100 calcium binding protein A10 | 6677833 | 0.50 | 0.13 | -0.38 |
| 489 | transketolase | 6678359 | 0.50 | 0.13 | -0.38 |
| 490 | myristoylated alanine rich protein kinase C substrate | 6678768 | 0.50 | 0.13 | -0.38 |
| 491 | acyl-Coenzyme A dehydrogenase, medium chain | 6680618 | 0.50 | 0.13 | -0.38 |
| 492 | fatty acid binding protein 3, muscle and heart | 6753810 | 0.50 | 0.13 | -0.38 |
| 493 | fatty acid binding protein 5, epidermal | 6754450 | 0.50 | 0.13 | -0.38 |
| 494 | annexin A2 | 6996913 | 0.50 | 0.13 | -0.38 |
| 495 | proteasome (prosome, macropain) subunit, alpha type 7 | 7106389 | 0.50 | 0.13 | -0.38 |
| 496 | cysteine and glycine-rich protein 3 | 7304987 | 0.50 | 0.13 | -0.38 |
| 497 | succinate-CoA ligase, GDP-forming, alpha subunit | 255958286 | 0.50 | 0.13 | -0.38 |
| 498 | isocitrate dehydrogenase 3 (NAD+) alpha | 18250284 | 0.50 | 0.13 | -0.38 |
| 499 | aldehyde dehydrogenase family 6, subfamily A1 | 19527258 | 0.50 | 0.13 | -0.38 |
| 500 | adenosine kinase | 19527306 | 0.50 | 0.13 | -0.38 |
| 501 | dihydrodiol dehydrogenase (dimeric) | 27229131 | 0.50 | 0.13 | -0.38 |
| 502 | myosin, heavy polypeptide 14 | 29336026 | 0.50 | 0.13 | -0.38 |
| 503 | enoyl Coenzyme A hydratase, short chain, 1, mitochondrial | 29789289 | 0.50 | 0.13 | -0.38 |
| 504 | glycogen synthase 1, muscle | 31560022 | 0.50 | 0.13 | -0.38 |
| 505 | aspartyl aminopeptidase | 161016820 | 0.50 | 0.13 | -0.38 |
| 506 | carboxymethylenebutenolidase-like (Pseudomonas) | 31712014 | 0.50 | 0.13 | -0.38 |
| 507 | dodecenoyl-Coenzyme A delta isomerase (3,2 trans-enoyl-Coenyme A isomerase) | 31981810 | 0.50 | 0.13 | -0.38 |
| 508 | dihydrolipoamide dehydrogenase | 31982856 | 0.50 | 0.13 | -0.38 |
| 509 | protein kinase, cAMP dependent regulatory, type II beta | 45598396 | 0.50 | 0.13 | -0.38 |
| 510 | neural precursor cell expressed, developmentally down-regulated gene 4 | 56699423 | 0.50 | 0.13 | -0.38 |
| 511 | phosphohistidine phosphatase | 58037409 | 1.00 | 0.63 | -0.38 |
| 512 | histocompatibility 2, Q region locus 7 | 310616733 | 1.00 | 0.63 | -0.38 |
| 513 | LIM domain binding 3 isoform c | 84875544 | 1.00 | 0.63 | -0.38 |
| 514 | zinc binding alcohol dehydrogenase, domain containing 1 | 85719320 | 1.50 | 1.13 | -0.38 |
| 515 | acetyl-Coenzyme A acetyltransferase 3 | 110625948 | 1.50 | 1.13 | -0.38 |
| 516 | protein disulfide isomerase associated 3 | 112293264 | 2.00 | 1.63 | -0.38 |
| 517 | biliverdin reductase A | 124487331 | 2.00 | 1.63 | -0.38 |
| 518 | chaperonin subunit 8 (theta) | 126723461 | 2.50 | 2.13 | -0.38 |
| 519 | calpain 1 | 6671668 | 0.50 | 0.00 | -0.50 |
| 520 | fumarylacetoacetate hydrolase | 240120112 | 0.50 | 0.00 | -0.50 |
| 521 | glutathione transferase zeta 1 (maleylacetoacetate isomerase) | 6754092 | 0.50 | 0.00 | -0.50 |
| 522 | protein kinase, cAMP dependent, catalytic, beta | 6755076 | 0.50 | 0.00 | -0.50 |
| 523 | S100 calcium binding protein A1 | 6755386 | 0.50 | 0.00 | -0.50 |
| 524 | 5',3'-nucleotidase, cytosolic | 7657031 | 0.50 | 0.00 | -0.50 |
| 525 | protein phosphatase 2a, catalytic subunit, alpha isoform | 9506983 | 0.50 | 0.00 | -0.50 |
| 526 | glutathione S-transferase, pi 1 | 10092608 | 0.50 | 0.00 | -0.50 |
| 527 | magnesium-dependent phosphatase-1 | 12963663 | 0.50 | 0.00 | -0.50 |
| 528 | small muscle protein, X-linked | | 0.50 | 0.00 | -0.50 |
| 529 | guanosine monophosphate reductase | 17975500 | 0.50 | 0.00 | -0.50 |
| 530 | RAS-related C3 botulinum substrate 3 | 18875380 | 0.50 | 0.00 | -0.50 |
| 531 | UDP-glucose pyrophosphorylase 2 | 21314832 | 0.50 | 0.00 | -0.50 |
| 532 | aldo-keto reductase family 7, member A5 (aflatoxin aldehyde reductase) | 240120054 | 0.50 | 0.00 | -0.50 |
| 533 | 3-monooxgenase/tryptophan 5-monooxygenase activation protein, gamma polypeptide | 31543976 | 0.50 | 0.00 | -0.50 |
| 534 | v-crk sarcoma virus CT10 oncogene homolog | 31559995 | 0.50 | 0.00 | -0.50 |
| 535 | phosphoglucomutase 1 | 33859686 | 0.50 | 0.00 | -0.50 |
| 536 | aminolevulinate, delta-, dehydratase | 34328485 | 0.50 | 0.00 | -0.50 |
| 537 | hypothetical protein LOC66273 | 40254393 | 0.50 | 0.00 | -0.50 |
| 538 | protein phosphatase 3, catalytic subunit, beta isoform | 45592930 | 0.50 | 0.00 | -0.50 |
| 539 | EH-domain containing 2 | 55742711 | 0.50 | 0.00 | -0.50 |
| 540 | hypothetical protein LOC433182 | 70794816 | 0.50 | 0.00 | -0.50 |
| 541 | adenylate kinase 2 isoform a | 77020262 | 0.50 | 0.00 | -0.50 |
| 542 | PREDICTED: calpain small subunit 2 | 254675215 | 0.50 | 0.00 | -0.50 |
| 543 | echinoderm microtubule associated protein like 4 | 167234433 | 0.50 | 0.00 | -0.50 |
| 544 | lipase, hormone sensitive isoform 1 | 87239970 | 1.00 | 0.50 | -0.50 |
| 545 | mannose phosphate isomerase | 91206392 | 1.00 | 0.50 | -0.50 |
| 546 | calpain, small subunit 1 | 110227381 | 1.00 | 0.50 | -0.50 |
| 547 | carbonyl reductase 1 | 113680352 | 1.00 | 0.50 | -0.50 |
| 548 | aldehyde dehydrogenase 9, subfamily A1 | 115334671 | 1.00 | 0.50 | -0.50 |
| 549 | LEM domain containing 3 | 145864461 | 1.00 | 0.50 | -0.50 |
| 550 | kelch repeat and BTB (POZ) domain containing 10 | 124487329 | 1.50 | 1.00 | -0.50 |
| 551 | four and a half LIM domains 3 | 130488506 | 2.00 | 1.50 | -0.50 |
| 552 | FK506 binding protein 1a | 6679803 | 1.00 | 0.38 | -0.63 |
| 553 | poly(rC) binding protein 1 | 6754994 | 1.00 | 0.38 | -0.63 |
| 554 | perilipin | 164698408 | 2.00 | 1.38 | -0.63 |
| 555 | thioredoxin-like 2 | 31981269 | 2.00 | 1.38 | -0.63 |
| 556 | PDZ and LIM domain 3 | 7948997 | 1.00 | 0.25 | -0.75 |
| 557 | liver glycogen phosphorylase | 268836255 | 1.00 | 0.25 | -0.75 |
| 558 | guanosine diphosphate (GDP) dissociation inhibitor 1 | 33859560 | 1.00 | 0.25 | -0.75 |
| 559 | myosin, light polypeptide kinase 2, skeletal muscle | 163644275 | 4.50 | 3.75 | -0.75 |
| 560 | tubulin, alpha 4 | 6678467 | 1.00 | 0.13 | -0.88 |
| 561 | glutamate oxaloacetate transaminase 1, soluble | 160298209 | 1.00 | 0.13 | -0.88 |
| 562 | sporulation protein, meiosis-specific, SPO11 homolog isoform a | 6755624 | 1.00 | 0.13 | -0.88 |
| 563 | glucose-6-phosphate dehydrogenase X-linked | 6996917 | 1.00 | 0.13 | -0.88 |
| 564 | quininoid dihydropteridine reductase | 21312520 | 1.00 | 0.13 | -0.88 |
| 565 | acyl-Coenzyme A dehydrogenase, long-chain | 31982520 | 1.00 | 0.13 | -0.88 |
| 566 | hypothetical protein LOC67078 | 40254507 | 1.00 | 0.13 | -0.88 |
| 567 | oxoglutarate dehydrogenase (lipoamide) | 85861164 | 1.50 | 0.63 | -0.88 |
| 568 | filamin C, gamma | 124487139 | 2.00 | 1.13 | -0.88 |
| 569 | lactate dehydrogenase B | 6678674 | 1.00 | 0.00 | -1.00 |
| 570 | nucleosome assembly protein 1-like 4 | 6679012 | 1.50 | 0.50 | -1.00 |
| 571 | citrate synthase | 13385942 | 1.50 | 0.50 | -1.00 |
| 572 | hypothetical protein LOC67971 | 13385968 | 1.50 | 0.50 | -1.00 |
| 573 | glucan (1,4-alpha-), branching enzyme 1 | 17975508 | 2.00 | 1.00 | -1.00 |
| 574 | protein phosphatase 1, catalytic subunit, beta isoform | 161484668 | 2.00 | 1.00 | -1.00 |
| 575 | DJ-1 protein | 55741460 | 3.00 | 2.00 | -1.00 |
| 576 | heat shock 70kDa protein 1-like | 124339838 | 7.00 | 6.00 | -1.00 |
| 577 | adenylosuccinate synthetase 1 | 6671519 | 1.50 | 0.38 | -1.13 |
| 578 | serine (or cysteine) proteinase inhibitor, clade B, member 6a | 6678097 | 1.50 | 0.38 | -1.13 |
| 579 | SMT3 supressor of mif two 3 homolog 2 | 19111164 | 1.50 | 0.38 | -1.13 |
| 580 | LIM domain binding 3 isoform a | 84872211 | 2.50 | 1.38 | -1.13 |
| 581 | cofilin 2, muscle | 6671746 | 1.50 | 0.25 | -1.25 |
| 582 | aldehyde dehydrogenase 1 family, member L1 | 27532959 | 2.00 | 0.75 | -1.25 |
| 583 | pyrophosphatase | 27754065 | 2.00 | 0.75 | -1.25 |
| 584 | phosphoglycerate kinase 1 | 70778976 | 2.50 | 1.25 | -1.25 |
| 585 | L-3-hydroxyacyl-Coenzyme A dehydrogenase | 111038118 | 19.50 | 18.25 | -1.25 |
| 586 | thyroid hormone-responsive protein | 6678345 | 1.50 | 0.13 | -1.38 |
| 587 | polymerase I and transcript release factor | 6679567 | 1.50 | 0.13 | -1.38 |
| 588 | annexin A11 | 160707921 | 2.00 | 0.63 | -1.38 |
| 589 | brain glycogen phosphorylase | 24418919 | 2.00 | 0.63 | -1.38 |
| 590 | glutaredoxin | 31981458 | 2.00 | 0.63 | -1.38 |
| 591 | acetyl-Coenzyme A carboxylase beta | 157042798 | 2.50 | 1.13 | -1.38 |
| 592 | procollagen, type XIV, alpha 1 | 226423922 | 5.00 | 3.63 | -1.38 |
| 593 | aldehyde dehydrogenase 2, mitochondrial | 6753036 | 2.50 | 1.00 | -1.50 |
| 594 | apolipoprotein B editing complex 2 | 6753098 | 2.00 | 0.38 | -1.63 |
| 595 | glutathione S-transferase, mu 3 | 33468899 | 2.00 | 0.38 | -1.63 |
| 596 | N-myc downstream regulated gene 2 | 7305305 | 2.00 | 0.25 | -1.75 |
| 597 | malate dehydrogenase 1, NAD (soluble) | 254540027 | 9.50 | 7.75 | -1.75 |
| 598 | glutamic pyruvic transaminase 1, soluble | 33413404 | 2.00 | 0.13 | -1.88 |
| 599 | glucose phosphate isomerase 1 | 254553458 | 2.50 | 0.50 | -2.00 |
| 600 | LRP16 protein | 170650601 | 6.00 | 4.00 | -2.00 |
| 601 | aldo-keto reductase family 1, member B3 (aldose reductase) | 160707894 | 6.00 | 4.00 | -2.00 |
| 602 | malate dehydrogenase 2, NAD (mitochondrial) | 31982186 | 6.50 | 4.50 | -2.00 |
| 603 | isocitrate dehydrogenase 1 (NADP+), soluble | 162417975 | 7.00 | 5.00 | -2.00 |
| 604 | ATP synthase, H+ transporting mitochondrial F1 complex, beta subunit | 31980648 | 2.50 | 0.38 | -2.13 |
| 605 | ATPase, Ca++ transporting, fast twitch 1 | 36031132 | 2.50 | 0.38 | -2.13 |
| 606 | glutathione S-transferase, mu 1 | 6754084 | 3.00 | 0.75 | -2.25 |
| 607 | glutamine synthetase | 31982332 | 6.00 | 3.75 | -2.25 |
| 608 | fructose bisphosphatase 2 | 122937183 | 2.50 | 0.13 | -2.38 |
| 609 | diazepam binding inhibitor isoform 2 | 6681137 | 2.50 | 0.00 | -2.50 |
| 610 | 3-hydroxyisobutyryl-Coenzyme A hydrolase | 22122625 | 4.00 | 1.50 | -2.50 |
| 611 | lumican | 160333372 | 3.00 | 0.38 | -2.63 |
| 612 | fast skeletal muscle troponin C | 6678371 | 3.50 | 0.50 | -3.00 |
| 613 | synuclein, gamma | 6755592 | 4.00 | 1.00 | -3.00 |
| 614 | aconitase 2, mitochondrial | 18079339 | 4.50 | 1.13 | -3.38 |
| 615 | adenosine monophosphate deaminase 1 (isoform M) | 114431240 | 6.00 | 2.63 | -3.38 |
| 616 | phosphofructokinase, muscle | 254553344 | 7.00 | 3.50 | -3.50 |
| 617 | murinoglobulin 1 | 31982171 | 12.00 | 8.50 | -3.50 |
| 618 | apolipoprotein A-II | 157951676 | 4.50 | 0.38 | -4.13 |
| 619 | hypothetical protein LOC244595 | 283135142 | 5.00 | 0.88 | -4.13 |
| 620 | glycerol-3-phosphate dehydrogenase 1 (soluble) | 6753966 | 18.00 | 13.50 | -4.50 |
| 621 | UMP-CMP kinase | 165377065 | 6.00 | 1.38 | -4.63 |
| 622 | phosphoglucomutase 2 | 227330633 | 17.00 | 12.13 | -4.88 |
| 623 | acetyl-Coenzyme A carboxylase alpha | 125656173 | 7.50 | 2.50 | -5.00 |
| 624 | myosin, heavy polypeptide 4, skeletal muscle | 67189167 | 7.50 | 2.38 | -5.13 |
| 625 | triosephosphate isomerase 1 | 226958349 | 23.50 | 16.00 | -7.50 |
| 626 | ATP citrate lyase | 29293809 | 20.00 | 12.38 | -7.63 |
| 627 | four and a half LIM domains 1 isoform 3 | 116517334 | 12.00 | 4.25 | -7.75 |
| 628 | fatty acid synthase | 93102409 | 72.00 | 63.63 | -8.38 |
| 629 | adenylate kinase 1 | 10946936 | 21.00 | 11.88 | -9.13 |
| 630 | parvalbumin | 31980767 | 57.00 | 47.50 | -9.50 |
| 631 | pyruvate kinase, muscle | 31981562 | 39.50 | 28.00 | -11.50 |
| 632 | aldolase 1, A isoform | 6671539 | 53.50 | 41.00 | -12.50 |
| 633 | creatine kinase, muscle | 6671762 | 146.00 | 132.13 | -13.88 |
| 634 | enolase 3, beta muscle | 6679651 | 27.50 | 12.25 | -15.25 |
| 635 | muscle glycogen phosphorylase | 6755256 | 35.00 | 19.75 | -15.25 |

*Average from 4 mice

**Average per day for LN samples collected at days 1 to 4 post challenge (4 mice per sample at days 1 to 3, one sample at day 4).
