# Supplementary material for: Whole Proteome Analysis of Mouse Lymph Nodes in Cutaneous Anthrax
Source: PLoS One. 2014 Oct 20;9(10):e110873. doi: 10.1371/journal.pone.0110873 (PMC4203832; doi:10.1371/journal.pone.0110873)
Supplement: Table S3 — Top-Scoring Clusters of the GO Terms Corresponding to the Proteins Upregulated in Infection. (DOCX) [file pone.0110873.s003.docx]

**Table S3. Top-Scoring Clusters of the GO Terms Corresponding to the Proteins Upregulated in Infection**

| **E score*** | **Term** | **Count** | **P Value** | **Fold Enrichment** | **Benjamini** | **Genes (GI numbers)** |
| --- | --- | --- | --- | --- | --- | --- |
| 10.3 | GO:0009611~response to wounding | 41 | 1.73E-15 | 4.5 | 1.63E-12 | 160358825, 110625994, 30578393, 33859809, 19527078, 257471003, 84871986, 6753060, 33563297, 20330802, 6753798, 254281348, 18252782, 148747558, 6679961, 251823822, 109627652, 6678079, 6755398, 6754950, 218156289, 163914390, 110347406, 126518317, 7304875, 6754696, 6681079, 162138926, 33563252, 31543113, 6679383, 13385306, 76881807, 45597447, 12963497, 6755394, 170172553, 6677843, 236465805, 15375312, 118130771, 160707956 |
|  | GO:0007599~hemostasis | 17 | 2.68E-11 | 9.2 | 5.46E-09 |  |
|  | GO:0050817~coagulation | 16 | 2.43E-10 | 8.8 | 2.97E-08 |  |
|  | GO:0050878~regulation of body fluid levels | 17 | 9.67E-10 | 7.3 | 1.11E-07 |  |
| 10 | GO:0006954~inflammatory response | 24 | 2.16E-08 | 4.1 | 1.65E-06 | 148747558, 109627652, 6754950, 6755398, 6678079, 163914390, 218156289, 110347406, 126518317, 7304875, 6754696, 162138926, 20330802, 33563297, 6679383, 76881807, 12963497, 6755394, 170172553, 6677843, 6753798, 254281348, 236465805, 15375312, 15617203, 11225264, 8393739, 31981890, 164519050, 19482160, 110347473, 213418055, 6680359, 118130771 |
|  | GO:0006952~defense response | 34 | 6.98E-08 | 2.9 | 4.56E-06 |  |
| 9.0 | GO:0034622~cellular macromolecular complex assembly | 31 | 5.71E-14 | 5.5 | 3.48E-11 | 119433657, 33859809, 19527078, 6671672, 6680836, 6680161, 254588110, 29789199, 30061387, 30089710, 13430890, 112807207, 7657357, 6754816, 125347376, 30061347, 31981100, 21746161, 78190507, 28916693, 30061405, 33563252, 6678465, 6678469, 30061379, 7106439, 21426893, 12963615, 19745156, 88014720, 27501448, 96975138, 6753060, 6679108 |
|  | GO:0006461~protein complex assembly | 20 | 7.49E-06 | 3.4 | 3.92E-04 |  |
| 6.4 | GO:0032956~regulation of actin cytoskeleton organization | 16 | 2.27E-11 | 10.2 | 5.94E-09 | 112363072, 9790141, 10946578, 6753262, 160837788, 6671672, 115496850, 21312654, 9790219, 110227377, 28916693, 224809382, 6753492, 7304993, 23956222, 31542143, 78190507, 6756037, 6753364, 153792001, 6679108, 7304875, 7106335 |
|  | GO:0033043~regulation of organelle organization | 21 | 3.07E-09 | 5.2 | 2.81E-07 |  |
|  | GO:0043254~regulation of protein complex assembly | 14 | 9.55E-09 | 8.3 | 7.95E-07 |  |
|  | GO:0044087~regulation of cellular component biogenesis | 15 | 6.46E-08 | 6.5 | 4.38E-06 |  |
|  | GO:0032535~regulation of cellular component size | 18 | 9.76E-07 | 4.3 | 5.76E-05 |  |
|  | GO:0030837~negative regulation of actin filament polymerization | 7 | 3.43E-05 | 10.7 | 0.00157 |  |
|  | GO:0051494~negative regulation of cytoskeleton organization | 7 | 0.00144 | 5.6 | 0.038587 |  |
|  | GO:0051129~negative regulation of cellular component organization | 8 | 0.010567 | 3.3 | 0.179931 |  |
| 6.1 | GO:0034622~cellular macromolecular complex assembly | 31 | 5.71E-14 | 5.5 | 3.48E-11 | 119433657, 33859809, 19527078, 6671672, 6680836, 6680161, 254588110, 29789199, 30061387, 30089710, 13430890, 112807207, 7657357, 6754816, 125347376, 30061347, 31981100, 21746161, 78190507, 28916693, 30061405, 33563252, 6678465, 6678469, 30061379, 7106439, 21426893, 12963615, 19745156, 88014720, 27501448, 124286826, 96975138, 6753060, 6679108, 20806109 |
|  | GO:0006334~nucleosome assembly | 12 | 2.62E-06 | 6.3 | 1.50E-04 |  |
|  | GO:0031497~chromatin assembly | 12 | 3.45E-06 | 6.1 | 1.91E-04 |  |
|  | GO:0065004~protein-DNA complex assembly | 12 | 3.94E-06 | 6.1 | 2.12E-04 |  |
|  | GO:0034728~nucleosome organization | 12 | 3.94E-06 | 6.1 | 2.12E-04 |  |
|  | GO:0006323~DNA packaging | 12 | 6.12E-05 | 4.6 | 0.002665 |  |
| 3.5 | GO:0006418~tRNA aminoacylation for protein translation | 9 | 2.11E-05 | 7.5 | 0.001043 | 30578393, 163954948, 34610207, 219275596, 262118273, 211065507, 27229277, 251823891, 33468931 |
|  | GO:0006399~tRNA metabolic process | 9 | 0.009231 | 3.1 | 0.165181 |  |
| 2.7 | GO:0050818~regulation of coagulation | 6 | 1.29E-04 | 11.5 | 0.005021 | 160358825, 251823822, 257471003, 226958456, 6753060, 236465805,6753060, 236465805 |
|  | GO:0050819~negative regulation of coagulation | 4 | 0.0013 | 17.1 | 0.035954 |  |
| 2.7 | GO:0002526~acute inflammatory response | 19 | 1.94E-12 | 9.0 | 7.12E-10 | 148747558, 109627652, 6754950, 6755398, 6678079, 163914390, 218156289, 110347406, 126518317, 7304875, 162138926, 20330802, 33563297, 6679383, 76881807, 6755394, 6753798, 6677843, 15375312, 213418055, 6754696, 110347473, 118130771, 170172553, 161353502, 6680684, 86476056, 158303322, 251823822, 160415217, 31981890, 6755987, 31543113, 6753950, 7304993, 62460366, 7305395, 6754208, 14149635 |
|  | GO:0006957~complement activation, alternative pathway | 5 | 8.31E-05 | 19.2 | 0.003529 |  |
|  | GO:0006956~complement activation | 7 | 2.97E-04 | 7.5 | 0.010595 |  |
|  | GO:0045087~innate immune response | 11 | 4.64E-04 | 3.9 | 0.015037 |  |
|  | GO:0006959~humoral immune response | 7 | 0.00266 | 5.0 | 0.064596 |  |
|  | GO:0051604~protein maturation | 9 | 0.003489 | 3.6 | 0.079711 |  |
| 2.6 | GO:0046164~alcohol catabolic process | 9 | 2.66E-04 | 5.3 | 0.009686 | 124486895, 114326546, 9790051, 18152793, 7305229, 6724311, 13384778, 7305027, 9256624, 254281348, 251823978, 7110683, 227330582 |
|  | GO:0006007~glucose catabolic process | 8 | 3.68E-04 | 5.9 | 0.012175 |  |
|  | GO:0016052~carbohydrate catabolic process | 9 | 0.001183 | 4.3 | 0.033284 |  |
|  | GO:0006096~glycolysis | 6 | 0.005439 | 5.2 | 0.112037 |  |
|  | GO:0005996~monosaccharide metabolic process | 11 | 0.027736 | 2.2 | 0.341954 |  |
| 2.4 | GO:0010033~response to organic substance | 28 | 3.20E-04 | 2.1 | 0.011206 | 86476056, 15617203, 21704144, 34610207, 31981890, 6671672, 96975138, 6753060, 163644329, 6754254, 20330802, 6753492, 148747546, 6724311, 148747558, 226958497, 6754208, 176865892, 6678079, 218156289, 7304875, 6680586, 6680229, 14149635, 76881807, 45597447, 37497112, 114326482 |
|  | GO:0009719~response to endogenous stimulus | 14 | 0.001046 | 2.9 | 0.030911 |  |
|  | GO:0034097~response to cytokine stimulus | 6 | 0.0013 | 7.2 | 0.035427 |  |
|  | GO:0009725~response to hormone stimulus | 12 | 0.003898 | 2.8 | 0.086498 |  |
| 1.7 | GO:0050880~regulation of blood vessel size | 7 | 0.00144 | 5.6 | 0.038587 | 113461998, 6671507, 6671672, 45597447, 12963497, 84871986, 163644329, 261823995 |
|  | GO:0003018~vascular process in circulatory system | 7 | 0.001785 | 5.4 | 0.045006 |  |
|  | GO:0042311~vasodilation | 5 | 0.004898 | 7.1 | 0.106244 |  |
| 1.5 | GO:0042743~hydrogen peroxide metabolic process | 5 | 0.00156 | 9.6 | 0.041148 | 110224442, 45597447, 84871986, 170172553, 148747558, 6671549, 6753136, 84871986, 163644329 |
|  | GO:0000302~response to reactive oxygen species | 5 | 0.013698 | 5.3 | 0.213679 |  |
|  | GO:0006979~response to oxidative stress | 7 | 0.025618 | 3.1 | 0.333634 |  |
| 1.3 | GO:0008104~protein localization | 31 | 0.013128 | 1.6 | 0.207474 | 31543974, 6680718, 29243942, 226874906, 116089273, 6680047, 6756041, 163644329, 51491845, 114326446, 29789199, 6753364, 8567340, 41350312, 6680722, 163310736, 29789080, 148747526, 19526463, 10946578, 125347376, 254540223, 11230802, 78190507, 6756037, 6679108, 225543319, 160333304, 19745156, 227116327, 88014720 |
|  | GO:0070727~cellular macromolecule localization | 15 | 0.025769 | 1.9 | 0.33067 |  |
| 1.1 | GO:0031099~regeneration | 5 | 0.002682 | 8.3 | 0.064247 | 160358825, 6679961, 7304875, 257471003, 84871986 |
|  | GO:0042246~tissue regeneration | 3 | 0.0374 | 9.6 | 0.396775 |  |
| 1.1 | GO:0009113~purine base biosynthetic process | 3 | 0.006389 | 23.0 | 0.124792 | 93102415, 96975138, 118601013, 96975138 |
|  | GO:0046112~nucleobase biosynthetic process | 3 | 0.021472 | 12.8 | 0.298584 |  |
|  | GO:0006144~purine base metabolic process | 3 | 0.031702 | 10.5 | 0.364594 |  |
| 1.1 | GO:0010638~positive regulation of organelle organization | 6 | 0.011004 | 4.4 | 0.183303 | 112363072, 153792001, 6679108, 31542143, 9790219, 78190507, 6756037, 6753364, 6680836, 6671672, 78190507 |
|  | GO:0046605~regulation of centrosome cycle | 3 | 0.012963 | 16.5 | 0.206906 |  |
|  | GO:0010564~regulation of cell cycle process | 6 | 0.041111 | 3.2 | 0.420065 |  |
|  | GO:0070507~regulation of microtubule cytoskeleton organization | 4 | 0.06611 | 4.3 | 0.544884 |  |
| 1.1 | GO:0019725~cellular homeostasis | 20 | 0.001613 | 2.2 | 0.041917 | 148747558, 21313642, 6753136, 110224442, 160707956, 110347487, 31560677, 257471003, 7305285, 42415475, 163644329, 68131562, 10946574, 20330802, 6679108, 6671549, 13386060, 45597447, 6755114, 18017602, 84871986, 6753492, 20806109, 21359820, 77682555, 254540223, 14149635, 145301578 |
|  | GO:0042592~homeostatic process | 28 | 0.00271 | 1.8 | 0.06406 |  |
|  | GO:0006879~cellular iron ion homeostasis | 5 | 0.006365 | 6.6 | 0.125676 |  |
| 0.7 | GO:0006508~proteolysis | 38 | 0.028944 | 1.4 | 0.349484 | 31982686, 110625994, 31981273, 247300942, 30519997, 257471003, 6755152, 71043961, 114326446, 170650724, 7110703, 33563297, 6753798, 261824000, 158303322, 109627652, 84452155, 8850219, 218156289, 163914390, 134031994, 6755863, 110347406, 126518317, 6681079, 116734870, 6755198, 6755196, 9845265, 244791124, 33563282, 225543319, 227116345, 13385306, 47523981, 7242197, 236465805, 15375312 |
| 0.6 | GO:0009116~nucleoside metabolic process | 7 | 0.003203 | 4.8 | 0.074331 | 7305395, 21704144, 21281687, 45544618, 96975138, 37700232, 118601013 |
|  | GO:0046128~purine ribonucleoside metabolic process | 4 | 0.023487 | 6.4 | 0.319488 |  |

* Enrichment score for a clustered of GO terms based on the scores of its members
