# Supplementary material for: Whole Proteome Analysis of Mouse Lymph Nodes in Cutaneous Anthrax
Source: PLoS One. 2014 Oct 20;9(10):e110873. doi: 10.1371/journal.pone.0110873 (PMC4203832; doi:10.1371/journal.pone.0110873)
Supplement: Table S4 — Top-Scoring Clusters of the GO Terms Corresponding to the Proteins Down-regulated in Infection. (DOCX) [file pone.0110873.s004.docx]

**Table S4. Top-Scoring Clusters of the GO Terms Corresponding to the Proteins Down-regulated in Infection**

| **E score*** | **Term** | **Count** | **P Value** | **Fold Enrichment** | **Benjamini** | **Genes** |
| --- | --- | --- | --- | --- | --- | --- |
| 19.2 | GO:0006006~glucose metabolic process | 28 | 1.12E-26 | 18.0 | 1.09E-23 | 31981562, 6996917, 254553344, 254553458, 226958349, 6754524, 254540027, 33859640, 24418919, 33859686, 31982186, 161484668, 70778976, 124486747, 31560022, 70794816, 6678674, 6754450, 122937183, 85861164, 6755256, 268836255, 17975508, 6679261, 6671539, 227330633, 6753966, 6679651, 91206392, 31981458, 255958286, 31982856, 227500281, 157951741, 162417975, 31980648, 18079339, 160298209, 13385942, 18250284, 6755911 |
|  | GO:0005996~monosaccharide metabolic process | 29 | 3.48E-24 | 14.0 | 8.45E-22 |  |
|  | GO:0044275~cellular carbohydrate catabolic process | 17 | 1.33E-18 | 26.2 | 2.58E-16 |  |
|  | GO:0046164~alcohol catabolic process | 17 | 5.48E-18 | 24.2 | 8.88E-16 |  |
|  | GO:0006096~glycolysis | 14 | 5.65E-16 | 29.4 | 5.40E-14 |  |
| 8.3 | GO:0015980~energy derivation by oxidation of organic compounds | 17 | 6.16E-15 | 16.0 | 5.49E-13 | 162417975, 157951741, 6755256, 268836255, 18079339, 254540027, 24418919, 17975508, 160298209, 13385942, 31982186, 18250284, 255958286, 161484668, 124486747, 31982856, 31560022, 162417975, 113680352, 124487331, 10092608, 6996917, 226958349, 29293809, 27532959, 33859640, 34328485 |
| 8.3 | GO:0009060~aerobic respiration | 8 | 1.06E-08 | 27.3 | 6.88E-07 | 162417975, 157951741, 6755256, 268836255, 18079339, 254540027, 24418919, 17975508, 160298209, 13385942, 31982186, 18250284, 255958286, 161484668, 124486747, 31982856, 31560022, 162417975, 113680352, 124487331, 10092608, 6996917, 226958349, 29293809, 27532959, 33859640, 34328485 |
|  | GO:0006099~tricarboxylic acid cycle | 7 | 1.21E-07 | 28.1 | 6.17E-06 |  |
|  | GO:0045333~cellular respiration | 9 | 2.05E-07 | 14.1 | 9.04E-06 |  |
| 2.4 | GO:0046395~carboxylic acid catabolic process | 6 | 0.001779 | 6.8 | 0.045706 | 31981810, 160298209, 22122625, 6680618, 240120112, 6754092 |
| 2.4 | GO:0016054~organic acid catabolic process | 6 | 0.001779 | 6.8 | 0.045706 | 31981810, 160298209, 22122625, 6680618, 240120112, 6754092 |
|  | GO:0009310~amine catabolic process | 5 | 0.005214 | 7.1 | 0.111454 | 160298209, 22122625, 161484634, 240120112, 6754092 |
|  |  |  |  |  |  |  |
| 1.6 | GO:0030029~actin filament-based process | 8 | 0.002913 | 4.2 | 0.06843 | 130488506, 55742711, 29336026, 6680924, 18875380, 7948997, 6681069, 124487139, 13385968, 6680924, 7948997, 130488506 |
| 1.6 | GO:0030036~actin cytoskeleton organization | 6 | 0.032693 | 3.4 | 0.401202 | 130488506, 55742711, 29336026, 6680924, 18875380, 7948997, 6681069, 124487139, 13385968, 6680924, 7948997, 130488506 |
|  | GO:0007010~cytoskeleton organization | 7 | 0.139245 | 2.0 | 0.798429 |  |
| 1.2 | GO:0046486~glycerolipid metabolic process | 6 | 0.012708 | 4.3 | 0.21264 | 157951741, 111378397, 87239970, 6754450, 15421856, 6753810, 157951676, |
| 1.2 | GO:0016125~sterol metabolic process | 4 | 0.049986 | 4.8 | 0.519526 | 157951741, 111378397, 87239970, 6754450, 15421856, 6753810, 157951676, |
|  | GO:0006638~neutral lipid metabolic process | 3 | 0.084146 | 6.2 | 0.670302 |  |
| 1.1 | GO:0006749~glutathione metabolic process | 3 | 0.027114 | 11.5 | 0.35938 | 162417975, 10092608, 6996917, 160298209, 6671519, 34328485, 114431240, 165377065, 31982332, 36031132, 21312520,165377065, 31980648, 19527306 |
| 1.1 | GO:0044271~nitrogen compound biosynthetic process | 11 | 0.001567 | 3.4 | 0.04263 | 162417975, 10092608, 6996917, 160298209, 6671519, 34328485, 114431240, 165377065, 31982332, 36031132, 21312520,165377065, 31980648, 19527306 |
|  | GO:0009165~nucleotide biosynthetic process | 7 | 0.011243 | 3.7 | 0.193849 |  |
|  | GO:0009142~nucleoside triphosphate biosynthetic process | 3 | 0.271925 | 2.9 | 0.947016 |  |
| 0.9 | GO:0045777~positive regulation of blood pressure | 3 | 0.019194 | 13.9 | 0.281425 | 31982393, 6996917, 31982511, 157951676, 55741460, 124339838, 254553344, 6755256, 6679803, 31981458, 112293264, 45592930, 160298209, 125656173, 6755911, 31981269, 31982856, 7304987 |
| 0.9 | GO:0042592~homeostatic process | 16 | 0.00154 | 2.5 | 0.043111 | 31982393, 6996917, 31982511, 157951676, 55741460, 124339838, 254553344, 6755256, 6679803, 31981458, 112293264, 45592930, 160298209, 125656173, 6755911, 31981269, 31982856, 7304987 |
|  | GO:0019725~cellular homeostasis | 9 | 0.031842 | 2.4 | 0.39789 |  |
|  | GO:0048878~chemical homeostasis | 9 | 0.043369 | 2.2 | 0.474407 |  |

* Enrichment score for a cluster of GO terms based on the scores of its members
