# Supplementary material for: Whole Proteome Analysis of Mouse Lymph Nodes in Cutaneous Anthrax
Source: PLoS One. 2014 Oct 20;9(10):e110873. doi: 10.1371/journal.pone.0110873 (PMC4203832; doi:10.1371/journal.pone.0110873)
Supplement: Table S5 — KEGG processes identified for lymph proteins up-regulated by infection. (DOCX) [file pone.0110873.s005.docx]

**Table S5. KEGG processes identified for lymph proteins up-regulated by infection**

|  | **Term** | **Count** | **P Value** | **Fold Enrich-ment** | **Bonferroni** | **GI Numbers** |
| --- | --- | --- | --- | --- | --- | --- |
| 1 | mmu04810:Regulation of actin cytoskeleton | 23 | 6.1E-06 | 3.0 | 7.70E-04 | 112363072, 9790141, 10946578, 160837788, 71037403, 31543942, 21312654, 11230802, 28916693, 114326446, 224809382, 83921618, 6753364, 51772073, 61097906, 6671509, 70778915, 242332572, 7304855, 31542143, 6753798, 6679601, 6681283 |
| 2 | mmu04610:Complement and coagulation cascades | 21 | 4.9E-13 | 7.9 | 6.19E-11 | 110625994, 30578393, 19527078, 33859809, 109627652, 6678079, 218156289, 163914390, 110347406, 126518317, 257471003, 33563252, 33563297, 6679383, 41235784, 76881807, 12963497, 6753798, 18252782, 236465805, 15375312 |
| 3 | mmu03050:Proteasome | 17 | 1.8E-12 | 10.2 | 2.23E-10 | 6755212, 261824000, 33859604, 158303322, 74315975, 247300942, 134031994, 27754103, 6755198, 20137004, 7110703, 6755196, 33563282, 228008337, 227116345, 6754724, 7242197 |
| 4 | mmu03010:Ribosome | 16 | 3.6E-07 | 5.1 | 4.53E-05 | 226958653, 31981515, 33504483, 6677813, 12584986, 31981100, 160333553, 9256519, 83699424, 30794450, 83745120, 9845265, 254675270, 254553321, 6677775, 6755372 |
| 5 | mmu05322:Systemic lupus erythematosus | 15 | 1.2E-05 | 4.1 | 0.00156 | 119433657, 126518317, 30061347, 6680161, 11230802, 6678143, 30061405, 13385598, 30061387, 33563297, 61097906, 30089710, 30061379, 7304855, 15375312 |
| 6 | mmu04510:Focal adhesion | 14 | 2.2E-02 | 2.0 | 0.94 | 125347376, 71037403, 31543942, 6755987, 11230802, 6753364, 61097906, 6671509, 7304855, 31542143, 163310736, 6679601, 6681283, 227116327 |
| 7 | mmu04520:Adherens junction | 12 | 6.0E-05 | 4.5 | 0.0077 | 6753364, 61097906, 6671509, 31542070, 242332572, 31542143, 7304855, 31543942, 11230802, 118130771, 6679601, 6681283 |
| 8 | mmu04670:Leukocyte transendothelial migration | 12 | 3.0E-03 | 2.9 | 0.31 | 83921618, 6753364, 61097906, 6671509, 70778915, 71037403, 31542143, 7304855, 31543942, 170172553, 11230802, 6679601 |
| 9 | mmu04666:Fc gamma R-mediated phagocytosis | 10 | 7.3E-03 | 2.9 | 0.60 | 224809382, 112363072, 9790141, 6753364, 51772073, 160837788, 170172553, 21312654, 28916693, 6679601 |
| 10 | mmu04612:Antigen processing and presentation | 9 | 1.4E-02 | 2.8 | 0.83 | 6755212, 254540166, 20137004, 6754254, 31981890, 31981690, 6680836, 40556608, 6681079 |
| 11 | mmu00010:Glycolysis / Gluconeogenesis | 8 | 9.5E-03 | 3.3 | 0.70 | 114326546, 9790051, 7110683, 18152793, 7305229, 6724311, 7305027, 9256624 |
| 12 | mmu00970:Aminoacyl-tRNA biosynthesis | 7 | 3.2E-03 | 4.7 | 0.33 | 34610207, 219275596, 262118273, 211065507, 27229277, 251823891, 33468931 |
| 13 | mmu00620:Pyruvate metabolism | 6 | 1.4E-02 | 4.1 | 0.83 | 251823978, 21450129, 7110683, 165932331, 18152793, 7305229 |
| 14 | mmu00071:Fatty acid metabolism | 6 | 2.0E-02 | 3.8 | 0.92 | 31560705, 21450129, 23956084, 82886628, 33859811, 6724311 |
| 15 | mmu00030:Pentose phosphate pathway | 5 | 1.2E-02 | 5.4 | 0.79 | 124486895, 9790051, 27532955, 227330582, 13384778 |
