# Supplementary material for: Whole Proteome Analysis of Mouse Lymph Nodes in Cutaneous Anthrax
Source: PLoS One. 2014 Oct 20;9(10):e110873. doi: 10.1371/journal.pone.0110873 (PMC4203832; doi:10.1371/journal.pone.0110873)
Supplement: Table S6 — KEGG processes identified for lymph proteins down-regulated by infection. (DOCX) [file pone.0110873.s006.docx]

**Table S6. KEGG processes identified for lymph proteins down-regulated by infection**

|  | **Term** | **Count** | **P Value** | **Fold Enrichment** | **Bonferroni** | **Genes** |
| --- | --- | --- | --- | --- | --- | --- |
| 1 | mmu00010:Glycolysis / Gluconeogenesis | 18 | 2.87E-14 | 12.1 | 3.09E-12 | 6678674, 122937183, 31981562, 254553344, 254553458, 115334671, 226958349, 31982511, 6754524, 6753036, 33859686, 6679261, 70778976, 6671539, 227330633, 31982856, 6679651, 70794816 |
| 2 | mmu00620:Pyruvate metabolism | 13 | 2.82E-11 | 14.5 | 3.05E-09 | 6678674, 157042798, 31981562, 115334671, 160707894, 6754524, 254540027, 6753036, 31982186, 125656173, 162139827, 6679261, 31982856 |
| 3 | mmu00640:Propanoate metabolism | 11 | 3.22E-10 | 16.8 | 3.47E-08 | 6678674, 157042798, 6753036, 29789289, 19527258, 125656173, 22122625, 255958286, 115334671, 6680618, 6754524 |
| 4 | mmu00020:Citrate cycle (TCA cycle) | 11 | 4.66E-10 | 16.2 | 5.04E-08 | 162417975, 254540027, 18079339, 13385942, 31982186, 85861164, 18250284, 6679261, 255958286, 31982856, 29293809 |
| 5 | mmu00030:Pentose phosphate pathway | 9 | 4.28E-08 | 15.8 | 4.62E-06 | 33859640, 122937183, 33859686, 6996917, 254553344, 254553458, 6678359, 6671539, 227330633 |
| 6 | mmu00500:Starch and sucrose metabolism | 10 | 4.42E-08 | 12.7 | 4.78E-06 | 24418919, 17975508, 33859686, 254553458, 21314832, 6755256, 268836255, 124486747, 227330633, 31560022 |
| 7 | mmu00071:Fatty acid metabolism | 9 | 4.18E-06 | 9.1 | 4.52E-04 | 31981810, 31982520, 6753036, 29789289, 111038118, 29126205, 115334671, 6680618, 31982511 |
| 8 | mmu00280:Valine, leucine and isoleucine degradation | 9 | 4.97E-06 | 8.9 | 5.37E-04 | 6753036, 29789289, 19527258, 111038118, 22122625, 29126205, 115334671, 6680618, 31982856 |
| 9 | mmu00051:Fructose and mannose metabolism | 7 | 1.21E-04 | 8.6 | 0.013 | 58037409, 122937183, 254553344, 91206392, 160707894, 6671539, 226958349 |
| 10 | mmu00380:Tryptophan metabolism | 7 | 1.90E-04 | 8.0 | 0.020282 | 157951741, 6753036, 29789289, 111038118, 6678281, 85861164, 115334671 |
| 11 | mmu00480:Glutathione metabolism | 7 | 8.14E-04 | 6.1 | 0.084231 | 162417975, 33468899, 255069715, 10092608, 6996917, 6754084, 6754092 |
| 12 | mmu00330:Arginine and proline metabolism | 6 | 0.005446 | 5.1 | 0.445522 | 160298209, 255069715, 6753036, 115334671, 31982332, 6671762 |
| 13 | mmu00980:Metabolism of xenobiotics by cytochrome P450 | 6 | 0.013586 | 4.1 | 0.771768 | 33468899, 10092608, 27229131, 6754084, 31982511, 6754092 |
| 14 | mmu00230:Purine metabolism | 9 | 0.019844 | 2.6 | 0.88522 | 7657031, 86198335, 6671519, 31981562, 114431240, 10946936, 19527306, 17975500, 77020262 |
| 15 | mmu04910:Insulin signaling pathway | 13 | 3.69E-05 | 4.3 | 0.00398 | 157042798, 122937183, 6755256, 268836255, 87239970, 24418919, 125656173, 31559995, 6755076, 161484668, 31560022, 45598396, 93102409 |
| 16 | mmu03320:PPAR signaling pathway | 9 | 2.74E-04 | 5.2 | 0.029156 | 157951676, 164698408, 31982520, 6681137, 6754450, 15421856, 6753810, 162139827, 6680618 |
