# Supplementary material for: Whole Proteome Analysis of Mouse Lymph Nodes in Cutaneous Anthrax
Source: PLoS One. 2014 Oct 20;9(10):e110873. doi: 10.1371/journal.pone.0110873 (PMC4203832; doi:10.1371/journal.pone.0110873)
Supplement: Table S7 — Proteins from naïve mice serum. (DOCX) [file pone.0110873.s007.docx]

**Table S7. Proteins from naïve mice serum**

|  | **Protein** | **GI number** | **Spectral hits*** |
| --- | --- | --- | --- |
| 1 | albumin 1 [Mus musculus] | 148673375 | 457.5 |
| 2 | hemoglobin, beta adult major chain [Mus musculus] | 31982300 | 311 |
| 3 | hemoglobin alpha 1 chain [Mus musculus] | 145301578 | 215 |
| 4 | transthyretin [Mus musculus] | 7305599 | 200 |
| 5 | hemoglobin, beta adult minor chain [Mus musculus] | 17647499 | 177.5 |
| 6 | complement component 3 [Mus musculus] | 28175786 | 166 |
| 7 | transferrin [Mus musculus] | 20330802 | 118 |
| 8 | retinol binding protein 4, plasma [Mus musculus] | 33859612 | 82 |
| 9 | pregnancy zone protein [Mus musculus] | 34785996 | 51 |
| 10 | apolipoprotein C-III [Mus musculus] | 15421856 | 34 |
| 11 | apolipoprotein A-I [Mus musculus] | 2145141 | 31.5 |
| 12 | serine (or cysteine) proteinase inhibitor, clade A, member 3K [Mus musculus] | 15079234 | 30 |
| 13 | serine (or cysteine) proteinase inhibitor, clade A, member 1c [Mus musculus] | 6678083 | 28 |
| 14 | major urinary protein 2 [Mus musculus] | 37748448 | 28 |
| 15 | PREDICTED: similar to odorant binding protein Ia [Mus musculus] | 1835143 | 27 |
| 16 | peroxiredoxin 2 [Mus musculus] | 148747558 | 24 |
| 17 | Similar to Serum amyloid A-4 protein precursor (Amyloid A-5 protein) [Mus musculus] | 6755398 | 22.5 |
| 18 | Similar to Complement C4 precursor isoform 5 [Mus musculus] | 94409900 | 19 |
| 19 | alpha-2-HS-glycoprotein [Mus musculus] | 7304875 | 17.5 |
| 20 | hemopexin [Mus musculus] | 18044757 | 17 |
| 21 | vitamin D-binding protein [Mus musculus] | 51172612 | 16.5 |
| 22 | glutathione peroxidase 3 [Mus musculus] | 15011841 | 16.5 |
| 23 | serine (or cysteine) proteinase inhibitor, clade C (antithrombin), member 1 [Mus musculus] | 18252782 | 16.5 |
| 24 | apolipoprotein E [Mus musculus] | 20381029 | 16 |
| 25 | serine (or cysteine) proteinase inhibitor, clade A, member 1d [Mus musculus] | 6678085 | 15.5 |
| 26 | complement component 8, gamma polypeptide [Mus musculus] | 58037159 | 15.5 |
| 27 | ceruloplasmin [Mus musculus] | 1224108 | 14 |
| 28 | cofilin 1, non-muscle [Mus musculus] | 6680924 | 14 |
| 29 | Similar to transgelin 2 [Mus musculus] | 6755714 | 13.5 |
| 30 | apolipoprotein C-IV [Mus musculus] | 6671501 | 13.5 |
| 31 | stromal antigen 3 [Mus musculus] | 148687246 | 11.5 |
| 32 | serine (or cysteine) proteinase inhibitor, clade A, member 1b [Mus musculus] | 76881807 | 10 |
| 33 | odorant binding protein Ia [Mus musculus] | 27151739 | 8.5 |
| 34 | esterase 1 [Mus musculus] | 553910 | 8 |
| 35 | paraoxonase 1 [Mus musculus] | 15215219 | 8 |
| 36 | coagulation factor II [Mus musculus] | 6753798 | 8 |
| 37 | apolipoprotein A-II [Mus musculus] | 157951676 | 8 |
| 38 | kininogen 1 [Mus musculus] | 12963497 | 7.5 |
| 39 | murinoglobulin 1 [Mus musculus] | 31982171 | 7.5 |
| 40 | apolipoprotein C-II [Mus musculus] | 148691224 | 7.5 |
| 41 | protease, serine, 1 [Mus musculus] | 16716569 | 7 |
| 42 | myoglobin [Mus musculus] | 21359820 | 7 |
| 43 | murinoglobulin 2 [Mus musculus] | 153945747 | 7 |
| 44 | histidine-rich glycoprotein [Mus musculus] | 11066003 | 6.5 |
| 45 | gelsolin [Mus musculus] | 28916693 | 6.5 |
| 46 | CAP, adenylate cyclase-associated protein 1 [Mus musculus] | 157951604 | 5 |
| 47 | inter alpha-trypsin inhibitor, heavy chain 4 [Mus musculus] | 226531047 | 5 |
| 48 | ADP-ribosylation factor 2 [Mus musculus] | 6671571 | 5 |
| 49 | kidney predominant protein NCU-G1 [Mus musculus] | 9910458 | 4.5 |
| 50 | hemoglobin Y, beta-like embryonic chain [Mus musculus] | 21450321 | 4 |
| 51 | nucleoside-diphosphate kinase 2 [Mus musculus] | 6679078 | 3.5 |
| 52 | serine (or cysteine) proteinase inhibitor, clade A, member 6 [Mus musculus] | 6680856 | 3.5 |
| 53 | apolipoprotein A-IV [Mus musculus] | 387102 | 3.5 |
| 54 | chemokine (C-X-C motif) ligand 4 [Mus musculus] | 9910486 | 3 |
| 55 | vitronectin [Mus musculus] | 6755987 | 3 |
| 56 | Similar to thrombospondin 1 [Mus musculus] | 29748078 | 2.5 |
| 57 | Similar to RAS related protein 1b [Mus musculus] | 223462165 | 2.5 |
| 58 | peptidylprolyl isomerase C [Mus musculus] | 6679441 | 2.5 |
| 59 | profilin 1 [Mus musculus] | 6755040 | 2.5 |
| 60 | Similar to WD repeat domain 76 [Mus musculus] | 133777670 | 2 |
| 61 | coagulation factor V [Mus musculus] | 6679731 | 2 |
| 62 | serine (or cysteine) proteinase inhibitor, clade A, member 3G [Mus musculus] | 86476056 | 2 |
| 63 | tissue inhibitor of metalloproteinase 3 [Mus musculus] | 6755793 | 2 |
| 64 | apolipoprotein N [Mus musculus] | 19527214 | 2 |
| 65 | histocompatibility 2, complement component factor B [Mus musculus] | 218156291 | 2 |
| 66 | haptoglobin [Mus musculus] | 8850219 | 1.5 |
| 67 | plasminogen [Mus musculus] | 200403 | 1.5 |
| 68 | carboxypeptidase N, polypeptide 2 homolog [Mus musculus] | 147904569 | 1.5 |
| 69 | histocompatibility 2, Q region locus 10 [Mus musculus] | 6754132 | 1.5 |
| 70 | alpha-2-glycoprotein 1, zinc [Mus musculus] | 148687277 | 1.5 |
| 71 | hypothetical protein LOC71775 [Mus musculus] | 21313642 | 1.5 |
| 72 | glutathione peroxidase 1 [Mus musculus] | 84871986 | 1.5 |
| 73 | complement component 9 [Mus musculus] | 15375312 | 1.5 |
| 74 | beta-2-microglobulin [Mus musculus] | 31981890 | 1.5 |
| 75 | fibrinogen, alpha polypeptide [Mus musculus] | 33563252 | 1.5 |
| 76 | serum amyloid P-component [Mus musculus] | 226958497 | 1 |
| 77 | nucleoside-diphosphate kinase 1 [Mus musculus] | 37700232 | 1.5 |
| 78 | apolipoprotein M [Mus musculus] | 9055162 | 1.5 |
| 79 | keratin complex 2, basic, gene 1 [Mus musculus] | 126116585 | 1 |
| 80 | mannose binding lectin (C) [Mus musculus] | 6754656 | 1 |
| 81 | actin related protein 2/3 complex, subunit 4 [Mus musculus] | 13386054 | 1 |
| 82 | histocompatibility 2, Q region locus 6 [Mus musculus] | 46559400 | 1 |
| 83 | PREDICTED: similar to murinoglobulin 1 [Mus musculus] | 29835160 | 0.5 |
| 84 | peroxiredoxin 5 precursor [Mus musculus] | 6755114 | 212 |
| 85 | destrin [Mus musculus] | 9790219 | 144 |
| 86 | kininogen 2 [Mus musculus] | 41235784 | 124 |
| 87 | glycosylphosphatidylinositol specific phospholipase D1 [Mus musculus] | 17512357 | 44 |
| 88 | eukaryotic translation initiation factor 5A [Mus musculus] | 31712036 | 41.5 |
| 89 | calcyclin binding protein [Mus musculus] | 33468885 | 37 |
| 90 | PREDICTED: similar to hemoglobin, beta adult major chain [Mus musculus] | 171854971 | 30.5 |
| 91 | proteasome (prosome, macropain) subunit, beta type 2 [Mus musculus] | 148698332 | 27.5 |
| 92 | carboxypeptidase N, polypeptide 1 [Mus musculus] | 13507644 | 24 |
| 93 | oral-facial-digital syndrome 1 gene homolog [Mus musculus] | 148708785 | 23.5 |
| 94 | peptidylprolyl isomerase B [Mus musculus] | 71774133 | 23 |
| 95 | actin related protein 2/3 complex, subunit 3 [Mus musculus] | 9790141 | 22 |
| 96 | serine (or cysteine) proteinase inhibitor, clade A, member 3M [Mus musculus] | 31418246 | 20 |
| 97 | ubiquitin-conjugating enzyme E2N [Mus musculus] | 18017605 | 20 |
| 98 | solute carrier family 24 (sodium/potassium/calcium exchanger), member 2 [Mus musculus] | 27369563 | 18 |
| 99 | PREDICTED: myosin regulatory light polypeptide 9 isoform 1 [Mus musculus] | 198278553 | 16 |
| 100 | PREDICTED: similar to myosin, light polypeptide 6, alkali, smooth muscle and non-muscle [Mus musculus] | 33620739 | 15 |
| 101 | tetranectin precursor [Mus musculus] | 171184425 | 13.5 |
| 102 | calponin 2 [Mus musculus] | 6680952 | 13.5 |
| 103 | proteosome (prosome, macropain) subunit, beta type 8 (large multifunctional protease 7) [Mus musculus] | 158303322 | 13 |
| 104 | thioredoxin 1 [Mus musculus] | 6755911 | 12 |
| 105 | inter-alpha trypsin inhibitor, heavy chain 1 [Mus musculus] | 15488640 | 11 |
| 106 | apolipoprotein C-I [Mus musculus] | 6680704 | 10.5 |
| 107 | ADP-ribosylation factor 4 [Mus musculus] | 6680720 | 10 |
| 108 | cytotoxic T lymphocyte-associated protein 2 alpha [Mus musculus] | 20306704 | 10 |
| 109 | proprotein convertase subtilisin/kexin type 9 [Mus musculus] | 23956352 | 9.5 |
| 110 | RAS-related C3 botulinum substrate 1 [Mus musculus] | 45592934 | 9.5 |
| 111 | androgen binding protein epsilon [Mus musculus] | 34452155 | 9.5 |
| 112 | serine (or cysteine) proteinase inhibitor, clade D, member 1 [Mus musculus] | 21961582 | 9 |
| 113 | fatty acid binding protein 5, epidermal [Mus musculus] | 6754450 | 8.5 |
| 114 | fatty acid binding protein 4, adipocyte [Mus musculus] | 14149635 | 8.5 |
| 115 | ubiquitin-conjugating enzyme E2L 3 [Mus musculus] | 6678481 | 8 |
| 116 | pro-platelet basic protein [Mus musculus] | 12963823 | 6.5 |
| 117 | major urinary protein 1 [Mus musculus] | 244792753 | 6.5 |
| 118 | thioredoxin domain containing 8 [Mus musculus] | 27229039 | 6 |
| 119 | torsin family 1, member B [Mus musculus] | 31559990 | 5.5 |
| 120 | ring finger protein 20 [Mus musculus] | 33859829 | 5.5 |
| 121 | histocompatibility 2, Q region locus 2 [Mus musculus] | 72535146 | 5.5 |
| 122 | coiled-coil domain containing 141 [Mus musculus] | 299829227 | 5 |
| 123 | Similar to Superoxide dismutase [Mus musculus] | 695629 | 5 |
| 124 | peptidylprolyl isomerase A [Mus musculus] | 6679439 | 5 |
| 125 | ADP-ribosylation factor 3 [Mus musculus] | 6680718 | 5 |
| 126 | adenylate kinase 1 [Mus musculus] | 10946936 | 4.5 |
| 127 | myosin light chain, regulatory B-like [Mus musculus] | 71037403 | 4.5 |
| 128 | Mediterranean fever [Mus musculus] | 9506893 | 4.5 |
| 129 | prostaglandin E synthase 3 (cytosolic) [Mus musculus] | 9790017 | 4 |
| 130 | ubiquitin C [Mus musculus] | 157671923 | 4 |
| 131 | cystatin E/M [Mus musculus] | 21312060 | 4 |
| 132 | ribonuclease, RNase A family 4 [Mus musculus] | 47679082 | 4 |
| 133 | apolipoprotein B-100 precursor [Mus musculus] | 161702988 | 4 |
| 134 | major urinary protein 3 [Mus musculus] | 88196796 | 4 |
| 135 | Similar to Complement factor I precursor (C3B/C4B inactivator) [Mus musculus] | 110347406 | 3.5 |
| 136 | Similar to carboxylesterase 1 [Mus musculus] | 192854 | 3.5 |
| 137 | Similar to Clusterin precursor (Sulfated glycoprotein 2) (SGP-2) (Clustrin) (Apolipoprotein J) (Apo-J) [Mus musculus] | 214010170 | 3.5 |
| 138 | serum amyloid A 1 [Mus musculus] | 6677843 | 3.5 |
| 139 | macrophage migration inhibitory factor [Mus musculus] | 6754696 | 3.5 |
| 140 | coactosin-like 1 [Mus musculus] | 19482160 | 3.5 |
| 141 | Rho, GDP dissociation inhibitor (GDI) beta [Mus musculus] | 33563236 | 3.5 |
| 142 | superoxide dismutase 1, soluble [Mus musculus] | 45597447 | 3.5 |
| 143 | phosphatidylethanolamine binding protein 1 [Mus musculus] | 84794552 | 3 |
| 144 | inter-alpha trypsin inhibitor, heavy chain 3 [Mus musculus] | 159110717 | 3 |
| 145 | actin, gamma, cytoplasmic 1 [Mus musculus] | 6752954 | 3 |
| 146 | cathelicidin antimicrobial peptide [Mus musculus] | 148677086 | 3 |
| 147 | proteasome (prosome, macropain) subunit, beta type 5 [Mus musculus] | 6755204 | 3 |
| 148 | G kinase anchoring protein 1 [Mus musculus] | 9789999 | 3 |
| 149 | androgen-binding protein eta [Mus musculus] | 10181188 | 3 |
| 150 | Lipocalin 11 [Mus musculus] | 34761569 | 3 |
| 151 | RAB8B, member RAS oncogene family [Mus musculus] | 27734154 | 3 |
| 152 | carbonic anhydrase 2 [Mus musculus] | 157951596 | 2.5 |
| 153 | Similar to calcyclin binding protein [Mus musculus] | 3142331 | 2.5 |
| 154 | leucine-rich repeat and WD repeat-containing protein KIAA1239 [Mus musculus] | 222418589 | 2.5 |
| 155 | PREDICTED: F-box and leucine-rich repeat protein 20 isoform 2 [Mus musculus] | 39930339 | 2.5 |
| 156 | Similar to Glyceraldehyde-3-phosphate dehydrogenase (GAPDH) [Mus musculus] | 6679937 | 2.5 |
| 157 | keratinocyte differentiation associated protein [Mus musculus] | 84781694 | 2.5 |
| 158 | Similar to Microtubule-associated protein RP/EB family member 1 (APC-binding protein EB1) (End-binding protein 1) (EB1) [Mus musculus] | 7106301 | 2.5 |
| 159 | S100 calcium binding protein A8 (calgranulin A) [Mus musculus] | 7305453 | 2.5 |
| 160 | PREDICTED: similar to cell division cycle 42 [Mus musculus] | 6753364 | 2.5 |
| 161 | crystallin, alpha B [Mus musculus] | 6753530 | 2.5 |
| 162 | inter-alpha trypsin inhibitor, heavy chain 2 [Mus musculus] | 226874935 | 2 |
| 163 | peroxiredoxin 1 [Mus musculus] | 6754976 | 2 |
| 164 | complement factor D precursor [Mus musculus] | 7304867 | 2 |
| 165 | lysozyme [Mus musculus] | 8393739 | 2 |
| 166 | SH3 domain binding glutamic acid-rich protein-like 3 [Mus musculus] | 18017602 | 2 |
| 167 | acylphosphatase 2, muscle type [Mus musculus] | 27229219 | 2 |
| 168 | glyoxalase 1 [Mus musculus] | 148676704 | 2 |
| 169 | selenoprotein P, plasma, 1 [Mus musculus] | 74271806 | 2 |
| 170 | parvalbumin [Mus musculus] | 31980767 | 2 |
| 171 | alpha-1-antitrypsin 1-1 isoform 1 precursor [Mus musculus] | 6678079 | 2 |
| 172 | complement component 1, q subcomponent, A chain [Mus musculus] | 6671650 | 2 |
| 173 | regenerating islet-derived 1 [Mus musculus] | 6677703 | 2 |
| 174 | nucleobindin 1 [Mus musculus] | 6679158 | 2 |
| 175 | carbonic anhydrase 1 [Mus musculus] | 145301561 | 2 |
| 176 | mannose binding lectin (A) [Mus musculus] | 6754654 | 2 |
| 177 | serum amyloid A 3 [Mus musculus] | 6755396 | 2 |
| 178 | apolipoprotein H [Mus musculus] | 18044699 | 1.5 |
| 179 | proteosome (prosome, macropain) subunit, beta type 9 (large multifunctional protease 2) [Mus musculus] | 8567394 | 1.5 |
| 180 | glutathione peroxidase 4 isoform 2 [Mus musculus] | 90903235 | 1.5 |
| 181 | secreted phosphoprotein 24 [Mus musculus] | 15082218 | 1.5 |
| 182 | apolipoprotein F [Mus musculus] | 19527216 | 1.5 |
| 183 | retinoic acid receptor responder (tazarotene induced) 2 [Mus musculus] | 21313658 | 1.5 |
| 184 | supervillin isoform 1 [Mus musculus] | 23346601 | 1.5 |
| 185 | hypothetical protein LOC103268 [Mus musculus] | 172072632 | 1.5 |
| 186 | complement component C1SB [Mus musculus] | 27805393 | 1.5 |
| 187 | cytochrome P450, family 3, subfamily a, polypeptide 41 [Mus musculus] | 28893549 | 1.5 |
| 188 | zinc finger protein 219 [Mus musculus] | 225543502 | 1.5 |
| 189 | necdin [Mus musculus] | 31981598 | 1.5 |
| 190 | cystatin C [Mus musculus] | 31981822 | 1.5 |
| 191 | G protein-coupled receptor 142 [Mus musculus] | 32306534 | 1.5 |
| 192 | insulin-like growth factor 1 isoform 2 [Mus musculus] | 34576545 | 1.5 |
| 193 | cutaneous T-cell lymphoma tumor antigen se70-2 [Mus musculus] | 123701991 | 1.5 |
| 194 | PREDICTED: nucleoporin 205 isoform 1 [Mus musculus] | 226437676 | 1.5 |
| 195 | kinesin family member 5B [Mus musculus] | 61657921 | 1.5 |
| 196 | protective protein for beta-galactosidase isoform b [Mus musculus] | 84042523 | 1.5 |
| 197 | Similar to Zinc finger protein 384 (Nuclear matrix transcription factor 4) (Cas-associated zinc finger protein) isoform 4 [Mus musculus] | 126352391 | 1.5 |
| 198 | PREDICTED: similar to prefoldin 5 isoform 1 [Mus musculus] | 237757295 | 1.5 |
| 199 | parvalbumin [Mus musculus] | 31980767 | 1.5 |
| 200 | alpha-1-antitrypsin 1-1 isoform 1 precursor [Mus musculus] | 6678079 | 1.5 |
| 201 | ADP-ribosylation factor 5 [Mus musculus] | 6680722 | 1.5 |
| 202 | cysteine and glycine-rich protein 1 [Mus musculus] | 6681069 | 1.5 |
| 203 | magnesium-dependent phosphatase-1 [Mus musculus] | 12963663 | 1.5 |
| 204 | acid phosphatase 1, soluble [Mus musculus] | 31542070 | 1.5 |
| 205 | UMP-CMP kinase [Mus musculus] | 165377065 | 1.5 |
| 206 | prolactin induced protein [Mus musculus] | 22135642 | 1.5 |
| 207 | RAB11B, member RAS oncogene family [Mus musculus] | 6679583 | 1 |
| 208 | RAB1, member RAS oncogene family [Mus musculus] | 6679587 | 1 |
| 209 | tudor domain containing 1 isoform 4 [Mus musculus] | 50251154 | 1 |
| 210 | complement component factor H [Mus musculus] | 41946979 | 1 |
| 211 | Tnf receptor-associated factor 7 [Mus musculus] | 14250337 | 1 |
| 212 | pancreatic polypeptide receptor 1 [Mus musculus] | 31543504 | 1 |
| 213 | coagulation factor X [Mus musculus] | 2664220 | 1 |
| 214 | transporter 2, ATP-binding cassette, sub-family B [Mus musculus] | 148678316 | 1 |
| 215 | Similar to proteoglycan 4 [Mus musculus] | 161086926 | 1 |
| 216 | Similar to Ubiquitin cross-reactive protein precursor (Interferon-stimulated protein 15) (IP17) [Mus musculus] | 1381586 | 1 |
| 217 | Similar to Ig kappa chain V-II region 26-10 [Mus musculus] | 125794 | 1 |
| 218 | alpha 1 microglobulin/bikunin [Mus musculus] | 6680684 | 1 |
| 219 | parotid secretory protein [Mus musculus] | 6679509 | 0.5 |
| 220 | methylenetetrahydrofolate dehydrogenase 1 [Mus musculus] | 148666659 | 0.5 |
| 221 | proteasome (prosome, macropain) subunit, beta type 6 [Mus musculus] | 31982099 | 0.5 |
| 222 | gene model 1553, [Mus musculus] | 148689411 | 0.5 |
| 223 | triggering receptor expressed on myeloid cells-like 1 [Mus musculus] | 58037247 | 0.5 |
| 224 | Similar to Ferritin light chain 1 (Ferritin L subunit 1) [Mus musculus] | 114326466 | 0.5 |

*Average from 3 mice
